# EST analysis on pig mitochondria reveal novel expression differences between developmental and adult tissues

**DOI:** 10.1186/1471-2164-8-367

**Published:** 2007-10-11

**Authors:** Karsten Scheibye-Alsing, Susanna Cirera, Michael J Gilchrist, Merete Fredholm, Jan Gorodkin

**Affiliations:** 1 Division of Genetics and Bioinformatics, IBHV, University of Copenhagen, Grønnegårdsvej 3, DK-1870 Frederiksberg, Denmark; 2 The Wellcome Trust/Cancer Research UK Gurdon Institute, Cambridge, CB2 1QN, UK

## Abstract

**Background:**

The mitochondria are involved in many basic functions in cells of vertebrates, and can be considered the power generator of the cell. Though the mitochondria have been extensively studied there appear to be only few expression studies of mitochondrial genes involving a large number of tissues and developmental stages. Here, we conduct an analysis using the PigEST resource [1] which contains expression information from 35 tissues distributed on one normalized and 97 non-normalized cDNA libraries of which 24 are from developmental stages. The mitochondrial PigEST resource contains 41,499 mitochondrial sequences.

**Results:**

The mitochondrial EST (Expressed Sequence Tag) sequences were assembled into contigs which covers more than 94 percent of the porcine mitochondrial genome, with an average of 976 EST sequences per nucleotide. This data was converted into expression values for the individual genes in each cDNA library revealing differential expression between genes expressed in cDNA libraries from developmental and adult stages. For the 13 protein coding genes (and several RNA genes), we find one set of six genes, containing all cytochrome oxidases, that are upregulated in developmental tissues, whereas the remaining set of seven genes, containing all ATPases, that are upregulated in adult muscle and brain tissues. Further, the *COX I *(Cytochrome oxidase subunit one) expression profile differs from that of the remaining genes, which could be explained by a tissue specific cleavage event or degradation pattern, and is especially pronounced in developmental tissues. Finally, as expected cDNA libraries from muscle tissues contain by far the largest amount (up to 20%) of expressed mitochondrial genes.

**Conclusion:**

Our results present novel insight into differences in mitochondrial gene expression, emphasizing differences between adult and developmental tissues. Our work indicates that there are presently unknown mechanisms which work to customize mitochondrial processes to the specific needs of the cell, illustrated by the different patterns between adult and developmental tissues. Furthermore, our results also provide novel insight into how in-depth sequencing can provide significant information about expression patterns.

## Background

The mammalian mitochondrion is a system of only few components. It consist of 13 protein coding genes, 22 tRNA, two rRNAs and possibly a few non-coding RNAs [2]. In spite of this, the mitochondrion is of great importance to the organism, and higher animals would likely not exist without functional mitochondria. Thus, the mitochondria are an essential part of many metabolic pathways, most notably generation of ATP through the oxidative phosphorylation system, and is unique among the cellular organelles, because it contains a genome of its own [3,4] (and references therein). The circular mitochondrial genome also deviates from the nuclear genome by being extremely compact in nature with almost no inter-spaced non-coding DNA between genes, furthermore, it has a special codon usage using only the 22 tRNAs to encode the amino acids. The compact nature of the mitochondrion is directly reflected in the transcription mechanism, as all genes are transcribed in polycistronic transcripts which are then processed to give the mature RNAs [3,4] (and references therein).

Since, the genes encoded in the mitochondrial DNA are used in pathways central to living organism, the patterns of expression can potentially provide considerable insights into the metabolic and biochemical mechanisms in different tissues. The massive amount of research on mitochondria (eg. a PubMed search with keywords 'mitochondrion' or 'mitochondrial' yields almost 150,000 hits) have to some extent uncovered the mechanisms responsible for regulation of mitochondrial genes: It has been found that the D-loop is the origin for transcription of both strands of the genome, and contains promoter and binding regions for transcription factors [5], that can serve to up or down regulate transcription. It has, for example, been shown that there is a significant regulation of transcription in response to external stimuli [6-8]. However, due to the nature of the polycistronic transcription, such genes are expected to be expressed at equal levels and be jointly up- and down-regulated *eg*. [7]. Furthermore, degradational mechanisms related to adenylation, stability, and translation have been linked to post-transcriptional regulation [9-11], which is expected to lead to uniform relative expression levels between mitochondrial genes. This coordinated expression of mitochondrial genes has been verified experimentally, by *eg*. [8,12]. Furthermore, the mitochondrial system is still being investigated for novel insights into disease mechanics, where a large scale expression analysis (as presented here) provide useful insights. To conduct these expression studies, good animal models are desirable, and the pig is an obvious candidate. It is increasingly being used as a model animal [13], since it is relatively close to humans, both genetically and physiologically, and thus a better model animal candidate than, for instance rodents. It has, for example, been shown that, in sequence space, the pig is closer to human than mouse [14] and the similar observation can readily be made for the mitochondrial genomes (data not shown). Additionally, pig (in contrast to human) provides easy access to tissues from various organs at different development stages. Also the pig is one of the worlds foremost production animals and any insights gained could have a large economic impact. Due to the extreme read-coverage of the mitochondrion, this study also provides predictions of the outcome from the "next"- generation sequencing methods (*ie*. 454 sequencing etc.) when applied to cDNA sequencing (see *eg*. [15]). These new methods are characterized by very high throughput and can therefore generate huge amounts of data, which can, for example, be used in digital expression studies, in the manner presented herein.

Here, we use the PigEST resource comprising 41,499 mitochondrial sequences [1,16] to present the first large-scale mitochondrial expression study comparing different tissues and different developmental stages.

## Results

### EST assembly

The Sino-Danish pig-genome project generated around 685,000 EST (Expressed Sequence Tags) sequences as described in [1,16]. The sequences were generated from one normalized and 97 non-normalized cDNA libraries covering in total 35 tissues. Details are available online [1]. The library names and corresponding tissues are listed in Table 1. The resource contains 41,499 EST sequences (reads) annotated as mitochondrial sequences. We assembled this set of mitochondrial sequences using the Distiller pipeline [17], and generated 35 contigs (gene clusters) and 23 single reads. The largest contig contains 11699 reads while the smallest were aggregates of only two ESTs. This also demonstrate the strength of the Distiller pipeline, as it is able to handle such large (and deep) clusters. A complete list of contigs along with their gene match in the mitochondrial genome is provided in Table 2.

**Table 1:** Overview of Libraries

**Lib name**	**Tissue **(Animals)	**Description**	**Reads**
amn	Amnion (S)	--	126
aor	Aorta (M)	--	99
bla	Bladder (M)	--	244
nbm*	Bone marrow (S)	115 days, bone marrow	135
cbe	Brain (M)	Cerebellum	325
cbr^•^	Brain (B)	Brain (cortex)	1067
fco	Brain (M)	Frontal cortex	410
hyp	Brain (S)	Hypothalamus	1153
pgl	Brain (M)	Pituitary gland	242
ecc*	Brain (S)	F 50 days, cortex	384
ece*	Brain (S)	F 50 days, cerebellum	206
fce*	Brain (S)	F 100, cerebellum	192
fcc*	Brain (S)	F 107, cortex cerebri	408
fhi*	Brain (S)	F 107 Hippocampus	662
cbl^•^	Haemopoetic (B)	Blood	200
jca	Cartillage (S)	Joint capsule	327
nca*	Cartillage (S)	115 days, cartilage	389
pan^•^	Endocrine glands (M)	Pancreas	60
ret	Eye (M)	Retina	901
eye*	Eye (S)	F 50, eye	174
fat	Fat (M)	Fat	742
che	Heart (B)	--	40
hea	Heart (M)	--	974
hlv	Heart (S)	Left ventricle	2103
cje	Intestine (B)	Jejunum	0
col	Intestine (S)	Large intest, colon asc.	342
duo	Intestine (S)	Small intest, duodenum	289
ill	Intestine (S)	Small intest, illeum	147
jej	Intestine (S)	Small intest, jejunum	629
lin	Intestine (M)	Large intestine	1109
sin	Intestine (M)	Small intestine	313
eje*	Intestine (S)	F 50, Jejunum	521
nco*	Intestine (S)	115 days, colon	548
nje*	Intestine (S)	115 days, jejunum	460
cki	Kidney (B)	--	10
kid	Kidney (M)	--	405
cli	Liver (B)	--	223
liv	Liver (M)	--	100
eli*	Liver (S)	F 50, liver	113
fli*	Liver (S)	F 100, liver	95
clu	Lung (B)	--	313
lun^‡^	Lung (M)	--	176
elu*	Lung (S)	F 50 days, lung	48
nlu*	Lung (S)	115 days, lung	239
cly	Lymphatic gland (B)	--	287
lyg	Lymphatic gland (M)	--	418
lnt	Lymphatic gland (S)	--	315
cga	Mammary gland (B)	--	69
mcp	Mammary gland (S)	Mammae, collostrum prod	257
mga	Mammary gland (M)	7 days after weaning	112
mgm^•^	Mammary gland (M)	14 days after birth	81
mgp	Mammary gland (M)	7 days pre birth	81
med	Mediastinum (S)	--	480
bfe	Muscles (M)	M. biceps femoris	1738
ctl^•^	Muscles (B)	Tenderloin	420
isp	Muscles (M)	M. infraspinatus	1530
ldo	Muscles (M)	M. longissimus dorsi	832
mas	Muscles (S)	M. masseter	1214
sme	Muscles (M)	M. semimembranosus	137
ssp	Muscles (M)	M. supraspinatus	806
ste	Muscles (M)	M. semitendinosus	711
tbr	Muscles (M)	M. triceps brachii	1314
vin	Muscles (M)	M. vastus intermedius	255
ese*	Muscles (S)	F 50, M. semitendinosus	279
nms*	Muscles (S)	115 days, M. semitendinosus	745
gul	Oesophagus (M)	--	291
ova	Ovary (M)	--	178
cov	Ovary (S)	--	363
pla^†^	Placenta (M)	--	15
pro	Prostata (M)	--	128
rec	Rectum (M)	--	393
cmu	Rhinal mucosal membrane (B)	--	5
nmm*	Rhinal mucosal membrane (S)	115 days, mucosal memb.	505
sag	Salivary gland (M)	--	628
csk	Skin (B)	--	19
ski	Skin (M)	--	312
ton	Skin (S)	Tip of tongue, mucosa	659
eep*	Skin (S)	F 50, epidermis	53
eru*	Skin (S)	F 50, regium bilicalis	229
nep*	Skin (S)	115 days, epidermis	143
spc	Spinal cord (M)	Spinal cord	570
ebs*	Spinal cord (S)	F 50 days, brain stem	408
fbs*	Spinal cord (S)	F 107 Brain stem	780
spl	Spleen (M)	--	126
csp	Spleen (B)	--	185
cst	Stomach (B)	--	3
sto	Stomach (M)	--	7
sug	Suprarenal glands (M)	--	424
cag	Suprarenal glands (B)	Adrenal gland	316
cte	Testicle (B)	--	2
tes	Testicle (M)	--	70
cty	Thyroid glands (B)	--	462
thg	Thyroid glands (M)	--	439
pty	Thyroid glands (S)	Piglet 2 days, thymus	288
fry*	Thyroid glands (S)	F 100, thymus	216
tra	Trachea (M)	--	336
ute	Uterus (S)	--	380
cut	Uterus (B)	--	6

*: Developmental tissue.

†: Is a normalized library.

•: Ignored in expression analysis (see materials and methods).

‡: Likely to be heavily contaminated by liver ESTs.

The generated cDNA libraries, representing 35 tissues. They are here shown as two (overlapping) sets, a physiological and a developmental set. The column "Libname" gives three letter code for the library, "Tissue" indicates the overall tissue the library has been generated from, where "(Animals)" indicates whether the library has been generated from a single (S or B) or multiple (M) animals. Libraries listed with (M) and (S) represent the pig breeds (mostly cross breeds) used in the Danish breeding (*ie*. Landrace, Yorkshire, Duroc and Hampshire), whereas the libraries listed with (B) present Chinese pig breeds. "Description" provides a short description. The column "Reads" show the number of mitochondrial reads that went into that library after cleaning. The F NN label refers to fetal tissue, NN days old, *eg*. F 50 libraries are tissues from 50 days old fetuses.

**Table 2:** Contig mapping

**Contig name**	**Genes**	**Expression Annotation**
Mto.1-rpt br0137_p9.5	ATP6, ATP8, tRNA-lys	ATP6, ATP8, tRNA-lys
Mto.1-rcbr0_003330.5	ATP6	
Mto.1-Pig4-TMW8011D08.5	CoIII	CoIII
Mto.1-rlin28_c19.5	CoIII	
Mto.1-ruio09_m24.5	COII, tRNA-asp	COII, tRNA-asp
Mto.1-rfce02c_c7.5	COII, tRNA-asp	
Mto.1-Pig3-SRG8019L17.3	COII	
Mto.1-rmcp16c_h16.5	tRNA-ala, -asn, -cys, -tyr	tRNA-ala, -asn, -cys, -tyr
Mto.1-rlnt16c_c19.5.5	NADH2, tRNA-met	NADH2, tRNA-met
Mto.1-raor035_i5.5	NADH2	
Mto.1-Pig1-12J20.5	NADH2	
Mto.1-rpigcf0_021006.5.5	12s rRNA	12s rRNA
Mto.1-rfhi4018b_b15.5	12s rRNA	
Mto.1-rfhi4034b_l17.5.5	NADH3	NADH3
Mto.1-rmas913b_n9.5	NADH3	
Mto.1-rpigca0_009382.5.264	NADH5, NADH6, tRNA-leu	NADH5, NADH6, tRNA-leu
Mto.1-rdbla0134_l12.5.265	NADH5, NADH6	
Mto.1-rcbr0_002402.5.88	NADH1, tRNA-leu	NADH1, tRNA-leu
Mto.1-risp19_019.5.109	NADH1, tRNA-leu	
Mto.1-rmed14c_h17.5	16s rRNA, tRNA-val	16s rRNA, tRNA-val
Mto.1-rcbr0_008583.5	16s rRNA	
Mto.1-reje01b_b4.5.221	CYTB	CYTB
Mto.1-rnje02c_k15.5.223	CYTB	
Mto.1-rjej10b_b21.5.172	NADH4, NADH4L	NADH4, NADH4L
Mto.1-rcbr0_013551.5.173	NADH4, NADH4L	
Mto.1-Pig3-SRG8014F12.3	NADH4	
Mto.1-Pig4-TMW8023L12.3	NADH4	
Mto.1-rece10_n9.5.336	COI, tRNA-ser	COI, tRNA-ser
Mto.1-rhlv24b_m17.5.334	COI, tRNA-ser	
Mto.1-rhyp06c_f16.5	COI	
Mto.1-Pig3-SRG8017A07.5	COI	

Table showing which genes the different contigs maps to. Found by comparing the matched (by BLAST) position to the annotations in genbank entry AF486866. The expression annotation column marks which clusters in the expression clustering the contig has gone into.

To ensure that the ESTs were unique for the porcine mitochondrial genome, the ESTs were matched against the nr database [18] using BLAST [19]. Except for 232 ESTs (which are probably still mitochondrial), all 41,499 mitochondrial ESTs matched with smaller E-values to mitochondrial proteins than all other proteins, where the vast majority (95%) matched the with E-values smaller than 1*e *- 65 (see Methods for details).

Furthermore, our assembly yield an overall high coverage of the mitochondrial genome as the contigs covers 15148 positions of 15978 possible, corresponding to 94% of the mitochondrial genome, and has an average coverage of 976. Interestingly, in spite of this extreme coverage, we observed that there are almost no ESTs originating in regions outside annotated genes, and we essentially did not pick up any ESTs originating from a completely unprocessed polycistronic transcript, *ie*. there is an abrupt change in coverage from genes to the small gaps between genes.

The assembly program, Distiller [17], was also able to conduct a phylogenetic decomposition of a number of clusters. That is, clusters originally composed of ESTs from both European (*eg*. Yorkshire) and Asian (*eg*. Taihu) pig breeds were divided into clusters with either European breed ESTs or Asian breed ESTs based on SNP information. A result of this is that there are five pairs of highly similar consensus sequences where one sequence match published Asian strain sequence while the other match published European strain sequences.

Another intricacy is overlapping genes, *eg*. ATP6 and ATP8. These were clustered together in the assembly due to the existence of EST sequences which overlap both genes, and therefore the clusters essentially represent both genes.

The phylogenetic decomposed pairs along with the gene(s) covered are indicated in Table 2.

### cDNA library content of mitochondrial genes

**Figure 1:**
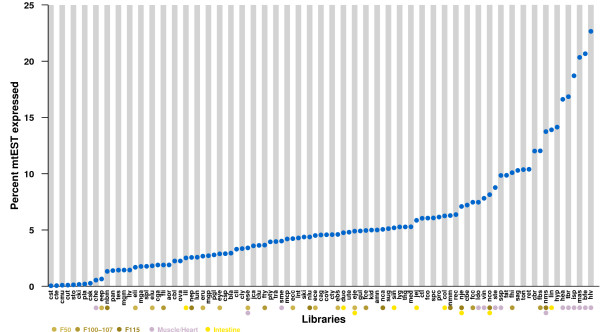
**Mitochondrial fraction of Expression**. The libraries are ordered according their fraction of EST originating from mitochondria. Selected tissues and their developmental stage are marked by colored bullets.

An investigation of the proportion of mitochondrial gene expression in the different cDNA libraries was performed. We found that cDNA libraries from tissues associated with high metabolism (*eg*. muscles) have a larger fraction of mitochondrial ESTs compared to the total number of reads from that library as shown in Figure 1. We observed that the fraction of total reads that originates from the mitochondrion ranges from roughly 0% up to 22%, with the majority falling between 2% to 10%. However, the libraries (8 in total) with the smallest fraction seem to have unusual low mitochondrial-EST counts, and were excluded in the following clustering. Furthermore, we attempted to see if there were any correlations between the number of counts from a given tissue and diversity of expression, however we did not find any such patterns.

### Differential gene expression

**Figure 2:**
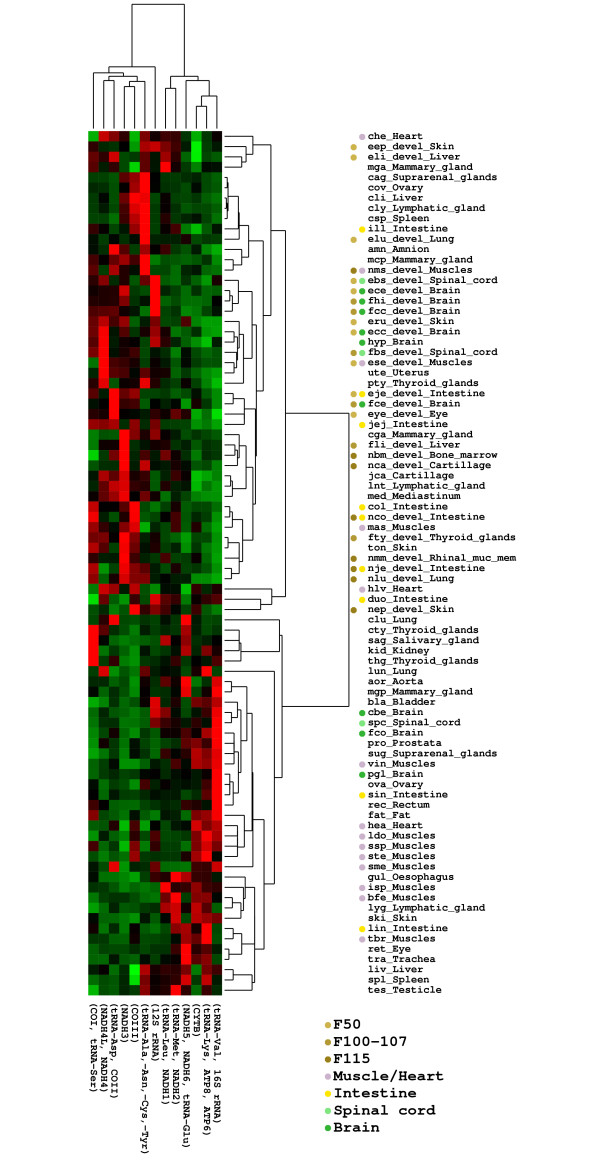
**Expression patterns**. Clustering of the genes based on their differential expression profiles. The expression level of a gene within a library is calculated from the number of EST from the clusters covering that gene, normalized by the total number of ESTs in the library. Phylogenetically decomposed clusters covering the same gene are merged into one entry (see Table 2). The different tissues and developmental stages are marked with color to emphasize the clustering.

To measure the digital expression of specific genes (contigs) in a given cDNA library, we considered the number of ESTs relative to the library size (see Methods), which in general, for high confident annotations (as here), has been shown to be in agreement with experimental qPCR results [16]. Using the digital expression values and merging clusters representing the same genes (*ie*. those that had been phylogenetically decomposed), we conducted a hierarchical clustering using the gene-cluster package [20] (with centroid linkage and correlation as similarity metric). The result is shown in Figure 2. We observed that the tissues fall in two different main groups: one enriched and one deprived for cDNA libraries established from tissues sampled at different developmental stages.

The confidence of the clustering was assessed using other clustering schemes and metrics (*eg*. complete linkage with euclidean similarity metric). Applying this range of schemes leads to essentially the same results. However, the exact relation between the individual libraries were to some extend shuffled from scheme to scheme, *ie*. the order of the libraries change slightly.

Though it is difficult to make any clear distinction between the genes that are expressed in the tissue clusters, it appears that 16S RNA and ATPase subunits six and eight have a higher expression in libraries from muscles and brain tissues, while there is no systematic pattern in the remaining libraries.

**Figure 3:**
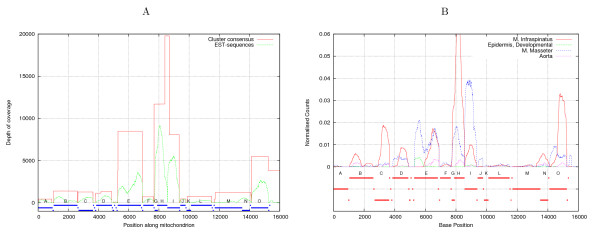
**Coverage of the mitochondrion**. The EST coverage of the mitochondrial genome. (A) The red line show the "depth" of the consensus sequences, while the green are the "true" coverage of the EST sequences. The lines at the bottom mark the position of known mitochondrial genes recorded in the genbank entry (Genbank: AF486866). (B) This shows the normalized coverage (expression) for four representative libraries, for which notable differences are observed. The individual genes are indicated by the lines at the bottom, and are: (A) 12s rRNA, (B) 16s rRNA, (C) NADH1, (D) NADH2, (E) COX I, (F) COX II, (G) ATP8, (H) ATP6, (I) COX III, (J) NADH3, (K) NADH4L, (L) NADH4, (M) NADH5, (N) NADH6, (O) CYTB.

Furthermore, there is a distinct difference between the total expression of the different mitochondrial genes. This is readily apparent from Figure 3(A), where the depth of the EST coverage is shown. Comparing the depth to the genes it appears that the depth varies considerable, from just below ten thousand to zero at gene boundaries. The differences in expression can be seen in more detail in Figure 3(B), where the normalized depth of coverage is shown from four representative libraries (see [additional file 1] for all libraries). We see that the expression pattern varies greatly for different tissues. Interestingly, we also find ESTs which map to the D-loop region (15271–15978), where no genes have been annotated. This is particularly intriguing considering the recent discovery of non-coding RNA (ncRNA) in the mouse D-loop region [2], however we were unable to find good matches (using BLAST [19] or FOLDALIGN [21] for local structural RNA alignment) between the putative mouse ncRNAs, and the porcine D-loop region (from Genbank: AF486866). Therefore, it has not been possible to conclude whether the porcine D-loop ESTs correspond to any of the reported mouse D-loop ncRNAs.

### Processing of mitochondrial transcripts

The EST data also contains information about post-transcriptional cleavage of the mitochondrial polycistronic transcript. At any given position on the mitochondrial genome, the coverage of an EST indicates the existence of a transcript from that position. Conversely, not finding any EST reads in a particular region means that the transcript is likely to be rapidly degraded. This is exemplified by the lack of coverage of the mitochondrial genome between annotated structures (genes/ncRNAs) as shown in Figure 3(A).

#### The tRNA genes

Due to the EST read coverage patterns of the tRNA genes it appears that there are two different mechanisms responsible for cleaving of tRNA genes within a polycistronic transcript, each associated either with the 5' or 3' end of the tRNA transcript. For the 5' end of the tRNAs there is no read coverage, whereas, there is some read coverage of the 3' end (which continues into the downstream protein coding region). This is in agreement with previous discovery where it was found that 5' cleavage precedes 3' end cleavage [22]. The fact that we only observe reads that overlap with the 3' end suggest that cleaving of the 3' end takes place at a longer timescale, or with a considerable weaker enzymatic strength (or smaller enzyme concentration) than the 5' cleavage.

#### The COX I transcript

Inspecting the position-wise EST coverage for the individual genes, we observe that the cytochrome oxidase I (COX I), (position 5270–6814 in Figure 3) gene differs from the remaining genes as its central region (position 5800–6000) has a profound dip in coverage with a low number of ESTs covering this region, see Figure 3(A). This is even more obvious when looking at the coverage of COX I in specific libraries (see [additional file 3]).

**Figure 4:**
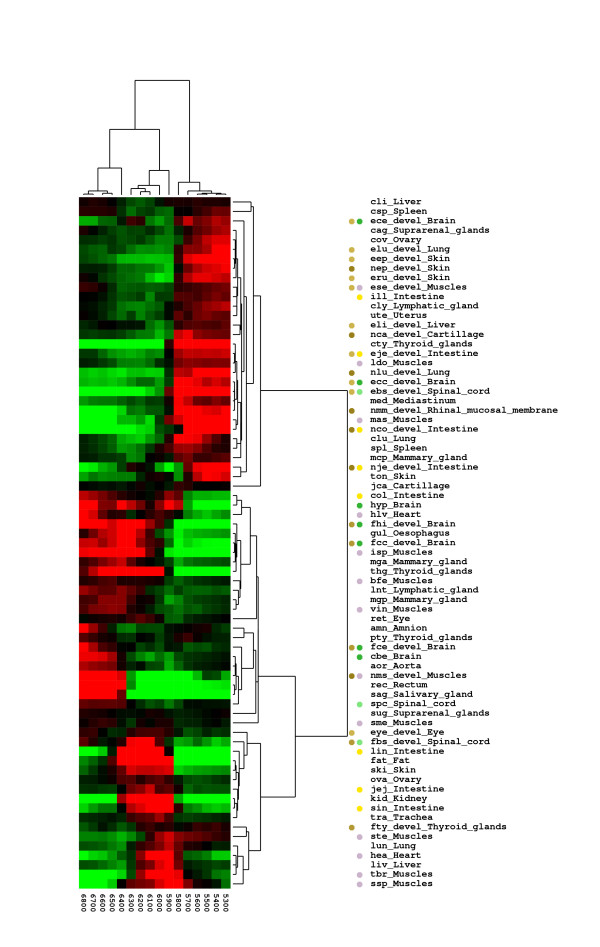
**Clustering of specific COX I gene coverage**. Clustering based specific coverage of the COX I gene. Three main group are found. Group A, with an elevated relative expression of the first part of the gene, and a drop off at position 5900, Group B with an elevated relative expression of the last part of the gene, and group C, with an elevated relative expression of the central part of the gene. Groups B and C are somewhat similar, but group A has a clear pattern.

Quantifying this we have created expression profiles for the individual libraries, based on the EST coverage along the gene. The expression profiles were created by calculating the average coverage for each window of 100 basepairs (from position 5300–6900), thus, each library has 16 numbers linked to coverage of each part of the COX I gene. These expression profiles were clustered using gene-cluster [20] resulting in three groups of libraries (see Figure 4).

**Figure 5:**
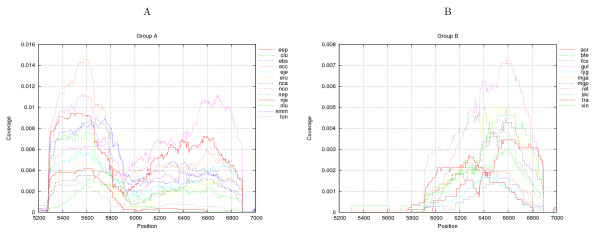
**Coverage of the COX I gene**. The coverage of the COX I gene (position 5270 to 6824). The division into groups, A, B, and C (not shown) of different libraries were performed by clustering on coverage of the different parts of the gene. (A) A subset of libraries from group A having the characteristic dip in expression level in the range approximately from position 5800 to 6000. (B) A subset of group B libraries having a general lack of expression within the region ranging from position 5270 to approximately 5900. The full plots for all groups are shown in [additional file 1], and plots for individual libraries can be found in [additional file 3].

For the first group (A), we observe the drop in EST coverage (Figure 5(A)), splitting the expression profile in two, with elevated coverage of the first part of the region, whereas for the second group (B) (Figure 5(B)) we observe EST coverage mainly in the region corresponding to the second part of the region. For the final group (C) a more uniform coverage is present, (see in [additional file 2], Figure 1). Furthermore, we observe that group A has an over-representation of developmental libraries, whereas groups B and C contains an over-representation of muscle libraries (see Figure 4). Furthermore, group B contains an over-representation of brain related libraries. These observations, of developmental and brain/muscle specific patterns, are in accordance with the similar type of observations made for the mitochondrial genome expression presented above.

We specifically investigated cDNA libraries from brain and spinal cord for COX I patterns. The sub-clustering of these libraries (see [additional file 2], Figure 2), shows that libraries established from early developmental stages (F 50: Ece, Ecc, Ebs) have a distinct group A expression pattern, while the remaining libraries have groups B and C pattens. This grouping fits well with the clustering of expressed non-mitochondrial brain related genes recently reported [16].

The known mitochondrial processing mechanisms work on RNA structures. We investigated whether any structural RNA signals were present in the gene by aligning the gene from different organisms (worm, fly, human, pig, among others), and look for compensating mutations. We found weak signals, but were not able to draw any conclusions from those.

## Discussion

Here, we investigated expression of porcine mitochondrial genes based on in depth EST sequencing. The ESTs were generated from one normalized and 97 non-normalized cDNA libraries representing in total 35 tissues of which 24 were from developmental stages [16]. We investigated 41,499 ESTs which yielded an average coverage of 976 EST per mitochondrial nucleotide position. The main observation was that there was great variability in expression between genes within different tissues and developmental stages. Interestingly, the differences in expression are particularly profound between (early) developmental and adult tissues, which in itself might explain why this previously has been overlooked, since apparently there are only a limited number of mitochondrial studies involving many developmental and adult tissues. The observations of differences in the expression pattern between genes and between tissues seem also to challenge the current knowledge of mitochondrial gene expression [3,4]. Since, mitochondrial genes are transcribed as long polycistronic transcripts [3,4] and the steady state level of the mRNAs is governed by degradation [11], the expectation is a fairly regular homogeneous expression pattern where the relative level of two genes (on the same transcript) would be tissue independent. However, our observations suggest that there exist mechanisms whereby the individual mRNAs are degraded in a tissue-specific fashion. The mitochondrial genes belonging to respiratory complex IV are clearly expressed at a higher level in tissues representing early developmental and new born stages. Conversely, the genes belonging to complex I are differential expressed in the two groups of tissues (*ie*. early developmental and adult). This might imply that there is a mutual dependence between full expression of the mitochondrial genome and adjustment to the adult cardio vascular circulation, *ie*. the differences are related to maturation of the energy metabolism. However, as all the mitochondrial genes are part of the same metabolic pathway, the direct biological interpretation is unclear, though the differences may suggest that the mitochondria plays a role in developmental stages beyond simple energy metabolism.

The differences between gene expression in developmental and adult tissues were also present in COX I. For this gene we observed (for several cDNA libraries) a sudden drop of EST coverage within the gene itself, indicating post-transcriptional processing. Based on the different expression patterns within the cDNA libraries we conducted a clustering that led to three main patterns of expression. One main group is dominated by cDNA libraries from developmental stages, whereas the others are dominated by brain and muscle libraries. Furthermore, for brain libraries we found COX I expression patterns in agreement with expression patterns from non-mitochondrial genes previously found [10], lending further credibility to our observation.

One could speculate that these patterns represent a cleavage event of the COX I gene. The putative cleavage event does not appear to be ubiquitous, and seems to be followed by specific degradation of one of the cleave-forms in some cases. The cleaving might represent a new mode of regulation of the mitochondrial processes where the COX I gene is involved. A previous study [23] suggested regulation of mitochondrial COX genes, emphasizing differences in developmental stages (in agreement with our study), and suggested a link to the different isoforms of COX VIa and COX VIIa.

We do not have any biological interpretation of how this mechanism works. Conventional mitochondrial processing would require the mRNA to be spliced due to secondary RNA structures [22], and though we have found weak RNA structure signals in the COX I gene (by comparing to other organisms), we can not conclusively say what mediates this processing. Extensive laboratory work could hopefully shed some light on the possible biological mechanism(s), but is beyond the scope of the work presented here.

## Conclusion

The discovery of large expression differences between individual mitochondrial genes is surprising. In particular the observation that there are large differences between mitochondrial gene expression in developmental and adult tissue is of potential great biological interest. Regulation of the mitochondrial genes is expected to some degree. However, the known mechanisms regulate the transcription of the full polycistronic transcript, and degrade the mitochondrial encoded RNA, dependent on the features of the RNA specie [8,10,24], leading to an expectation of uniform patterns across tissues. Our work indicates that there must be some mechanism(s) that selectively degrades specific gene transcripts in specific tissues. These mechanisms could partly be related to metabolic differences in adult and developmental tissues. Our study also demonstrates the usefulness of in-depth sequencing for expression studies, supporting that expression studies can be carried out by raw sequencing, such as the recent 454 and solexa parallel sequencing methods [25,26].

## Methods

### Data

The source of the EST sequences is described in [16]. The EST sequences from the sources described therein were matched against a known pig mitochondrial sequence extracted from GenBank (acc: AF486866, gi: 33320837) using blastn [19], and labeled as mitochondrial by using the build in tool of the Distiller assembler. In total 41,499 ESTs were assigned such label. These ESTs were then assembled with the Distiller EST assembly pipeline [17], which uses a backbone Microsoft SQL server. Further, based on SNP patterns, the contigs were phylogenetically split into European and Asian strains.

The generated contigs were then matched using blastn [19]) against a known pig mitochondrial sequence (GenBank: AF486866), thereby locating each sequence at a specific position on the mitochondrial genome, and allowing detailed comparison with known mitochondrial features. Extraction and analysis of the sequence information was performed using a number of SQL and Perl scripts created for the specific analysis.

The coverage depths were calculated by mapping the EST sequences to the mitochondrion in a similar fashion to the contigs, and simply adding the number of ESTs covering that particular position, *ie*. if two ESTs were mapped to cover a base on the mitochondrial genome, then the coverage at that positions is two. The counts were normalized using the total number of library specific reads.

To ensure that the porcine ESTs matched against protein databases are correct matches to mitochondrial proteins, we performed some validating checks. We (in lack of a complete porcine genome) matched human mitochondrial genes to known human genes. We found no matches with a high similarity, thus indicating that there are no genes paralogous to mitochondrial genes in mammals.

Furthermore, when matching the porcine ESTs (using BLAST [19]) against the known nr database [18], all but 232 ESTs hit nr sequences that were clearly identifiable as mitochondrial with a higher score (lower E-value respectively) than non-mitochondrial sequences. Also 95% of the ESTs match with E-values smaller than 1*e *- 65 to the mitochondrial sequences. For the remaining 232 most often a mitochondrial sequence matched with comparable E-value as the match with the lowest E-value. Further, for these ESTs the nr sequence that matched with the lowest E-value had unclear annotations, *ie*. annotations which could not conclusively be identified as belonging to a specific genome. We therefore believe that the 232 sequences represent mitochondrial ESTs, and included them in the analysis.

Additionally, it is worth noticing that the phylogenetic decomposition of genes into European and Asian strains, increase the confidence that all ESTs are mitochondrial, as others would have been likely to merge into a separate cluster and be decomposed along with the already decomposed gene-clusters.

### Digital Expression Profiles

The expression of gene *i *(contig) in library *j *is computed as the fraction of ESTs of library *j *that is assembled into gene *i*, that is the expression is #ESTlibj,geneiTotal #ESTlibj
 MathType@MTEF@5@5@+=feaafiart1ev1aaatCvAUfKttLearuWrP9MDH5MBPbIqV92AaeXatLxBI9gBaebbnrfifHhDYfgasaacH8akY=wiFfYdH8Gipec8Eeeu0xXdbba9frFj0=OqFfea0dXdd9vqai=hGuQ8kuc9pgc9s8qqaq=dirpe0xb9q8qiLsFr0=vr0=vr0dc8meaabaqaciaacaGaaeqabaqabeGadaaakeaadaWcaaqaaiabcocaJiabbweafjabbofatjabbsfaunaaBaaaleaacqWGSbaBcqWGPbqAcqWGIbGydaWgaaadbaGaemOAaOgabeaaliabcYcaSiabdEgaNjabdwgaLjabd6gaUjabdwgaLnaaBaaameaacqWGPbqAaeqaaaWcbeaaaOqaaiabbsfaujabb+gaVjabbsha0jabbggaHjabbYgaSjabbccaGiabcocaJiabbweafjabbofatjabbsfaunaaBaaaleaacqWGSbaBcqWGPbqAcqWGIbGydaWgaaadbaGaemOAaOgabeaaaSqabaaaaaaa@5017@. The calculated expression patterns were then clustered using gene-cluster [20] (normalizing and centering data, centroid clustering based on correlations (uncentered)), and visualized with Java Treeview [27,28]. A few libraries were excluded in the analysis as they seemed to have artificially low read count, the cut-off was set at an absolute count of 20 ESTs (excluding the libraries: cst, cfe, cmu, cut, sto, cki, pla, csk). Since 'pla' is the normalized library it was expected to have a low read count. Furthermore, clusters that had been phylogenetically split were merged, so that the expression of one gene represented by two clusters were taken as the sum of the expression in the clusters.

## Availability and requirements

All data for the project including the assembled sequences (contigs), expression and BLAST data is available at . From this webpage it is also possible to access the assembly for each contig with its corresponding reads.

## Authors' contributions

MJG, MF and JG formulated the project. KSA conducted the assembly and expression analysis. All authors contributed to and approved the final manuscript.

## Supplementary Material

Additional file 1 Normalized coverage from libraries. The expression profile of the mitochondria for each cDNA library. Click here for file

Additional file 3 Normalized COXI coverage from libraries. The expression profile of the COX I gene for each cDN A library. Click here for file

Additional file 2 COXI expression and clustering. Supplementary figures for COX I expression and cluster analysis. Click here for file
