# Supplementary material for: EST analysis on pig mitochondria reveal novel expression differences between developmental and adult tissues
Source: BMC Genomics. 2007 Oct 11;8:367. doi: 10.1186/1471-2164-8-367 (PMC2194790; doi:10.1186/1471-2164-8-367)
Supplement: Additional file 1 — Normalized coverage from libraries. The expression profile of the mitochondria for each cDNA library. [file 1471-2164-8-367-S1.pdf]

Additional Data File 1: Normalized coverage from libraries

EST analysis on pig mitochondria reveal novel expression differences between developmental and adult tissues

Karsten Scheibye-Knudsen[1], Susanna Cirera[1], Michael J. Gilchrist[2], Merete Fredholm[1], Jan Gorodkin*[1]

[1]Division of Genetics and Bioinformatics, IBHV, University of Copenhagen, Grnnegrdsvej 3, DK-1870 Frederiksberg, Denmark

[2]The Wellcome Trust/Cancer Research UK Gurdon Institute, Cambridge, CB2 1QN, UK

Email: Karsten Scheibye-Knudsen - scheibye@genome.ku.dk; Susanna Cirera - scs@life.ku.dk; Michael J. Gilchrist - m.gilchrist@gurdon.cam.ac.uk; Merete Fredholm - mf@life.ku.dk; Jan Gorodkin*- gorodkin@genome.ku.dk;

*Corresponding author

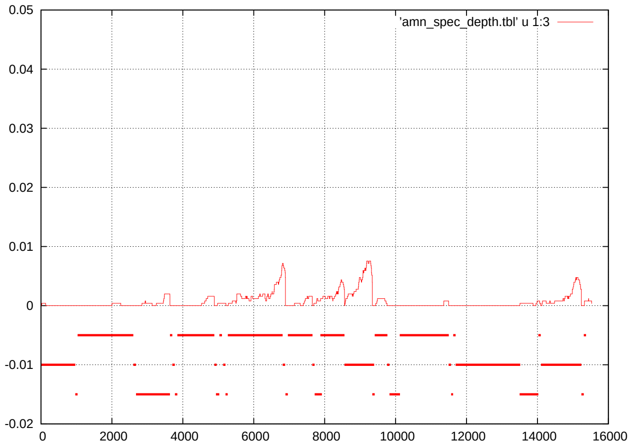
'amn_spec_depth.tbl' u 1:3

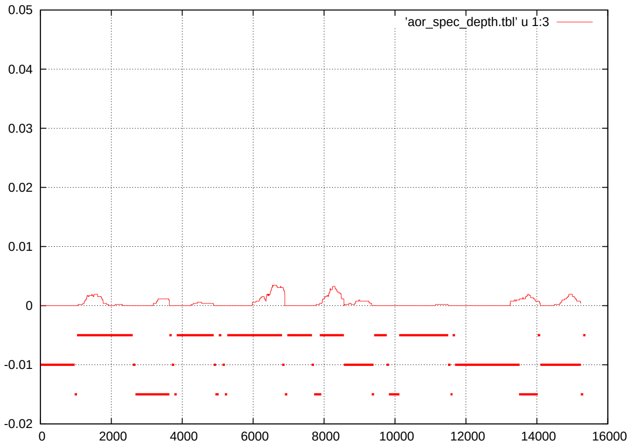
'aor_spec_depth.tbl' u 1:3

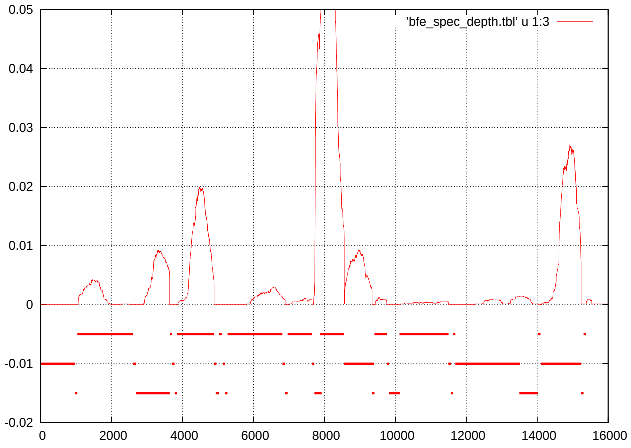
'bfe_spec_depth.tbl' u 1:3

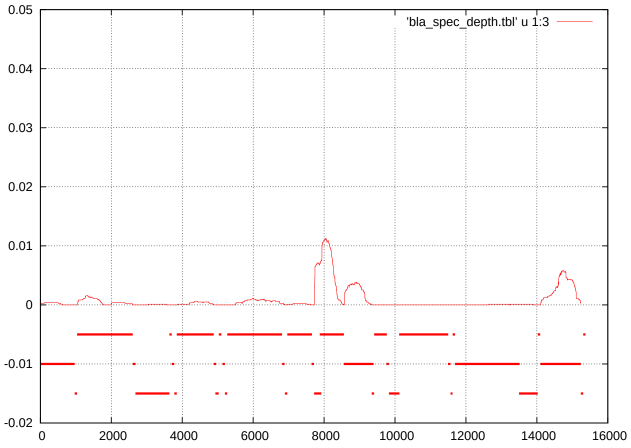
'bla_spec_depth.tbl' u 1:3

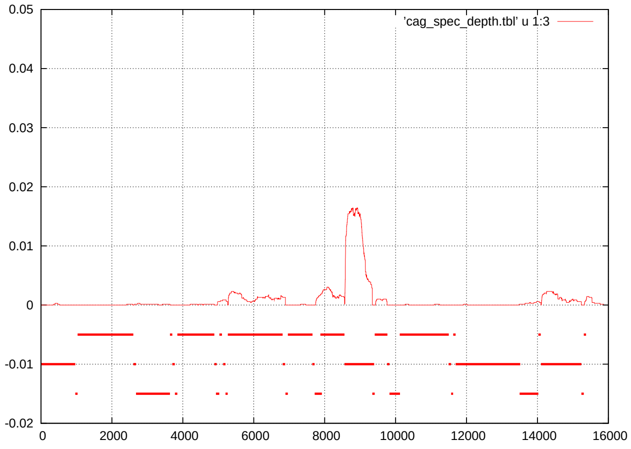
'cag_spec_depth.tbl' u 1:3

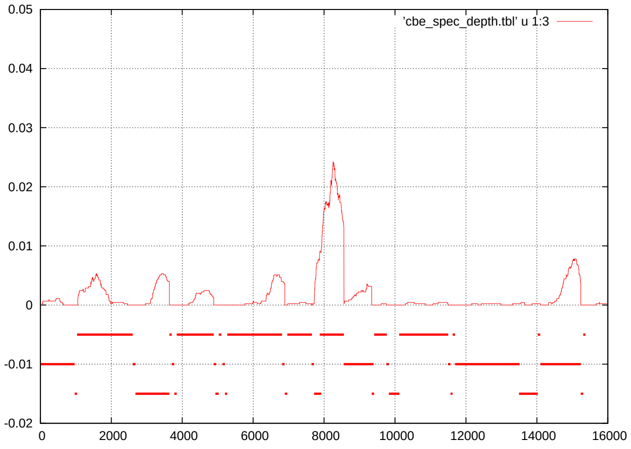
'cbe_spec_depth.tbl' u 1:3

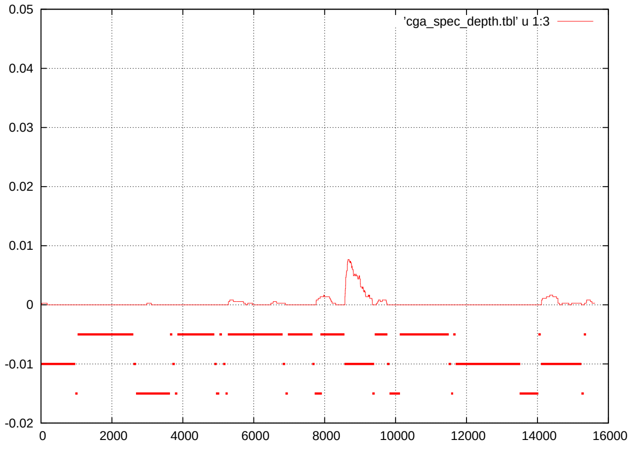
'cga_spec_depth.tbl' u 1:3

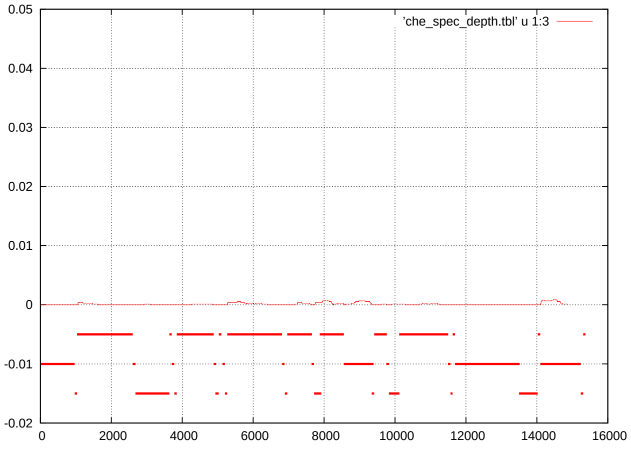
'che_spec_depth.tbl' u 1:3

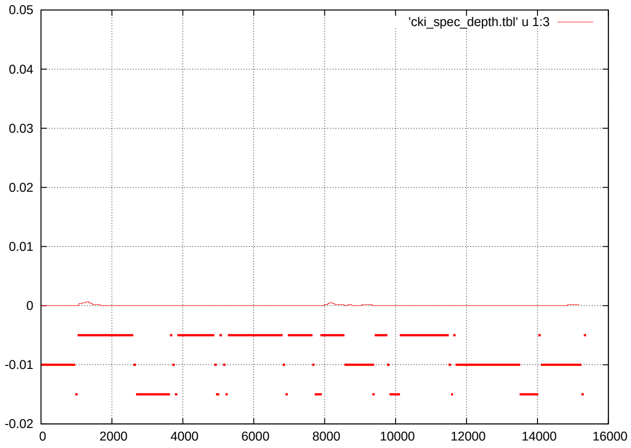
'cki_spec_depth.tbl' u 1:3
0.05
0.04
0.03
0.02
0.01
0
-0.01
-0.02
0
2000
4000
6000
8000
10000
12000
14000
16000

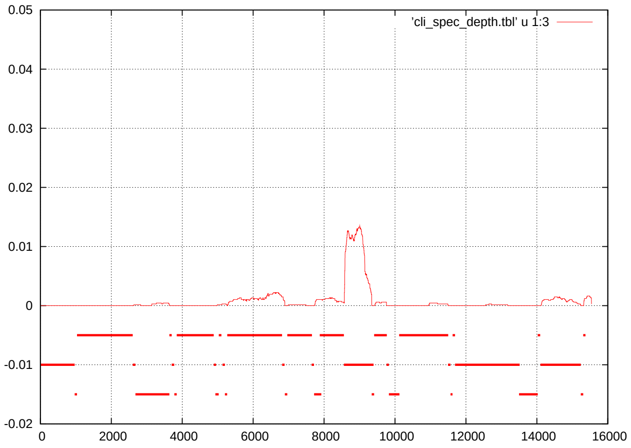
'cli_spec_depth.tbl' u 1:3
0.05
0.04
0.03
0.02
0.01
0
-0.01
-0.02
0
2000
4000
6000
8000
10000
12000
14000
16000

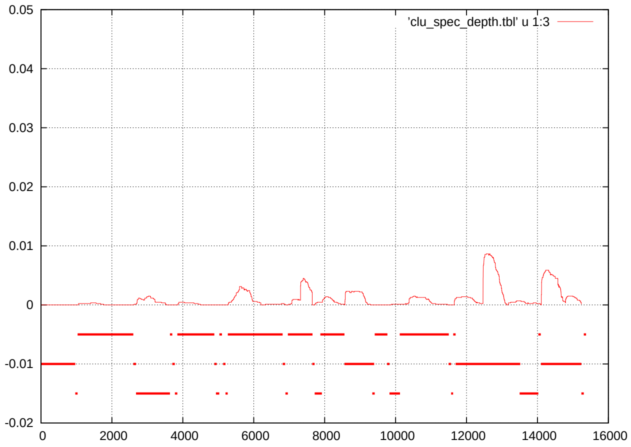
'clu_spec_depth.tbl' u 1:3
0.05
0.04
0.03
0.02
0.01
0
-0.01
-0.02
0
2000
4000
6000
8000
10000
12000
14000
16000

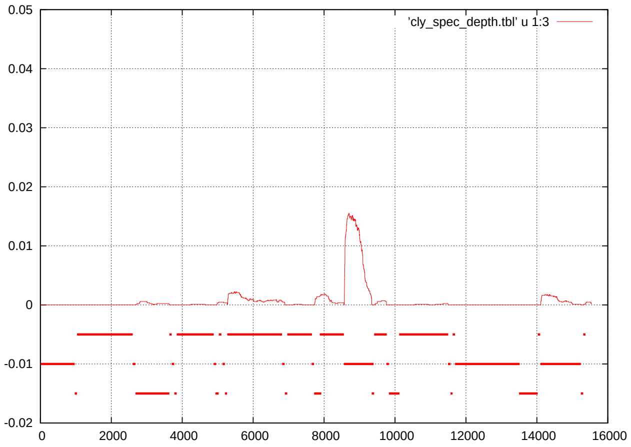
'cly_spec_depth.tbl' u 1:3
0.05
0.04
0.03
0.02
0.01
0
-0.01
-0.02
0
2000
4000
6000
8000
10000
12000
14000
16000

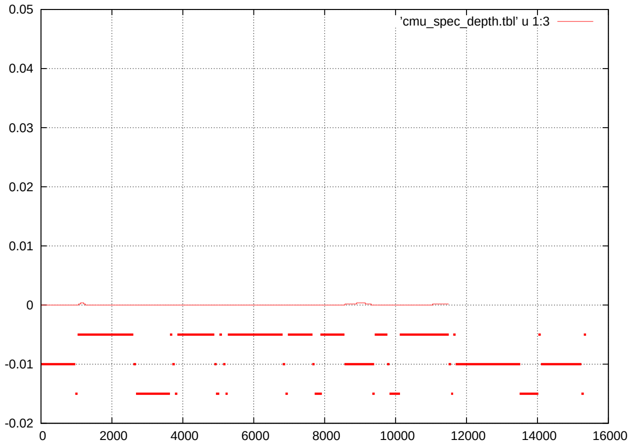
'cmu_spec_depth.tbl' u 1:3
0.05
0.04
0.03
0.02
0.01
0
-0.01
-0.02
0
2000
4000
6000
8000
10000
12000
14000
16000

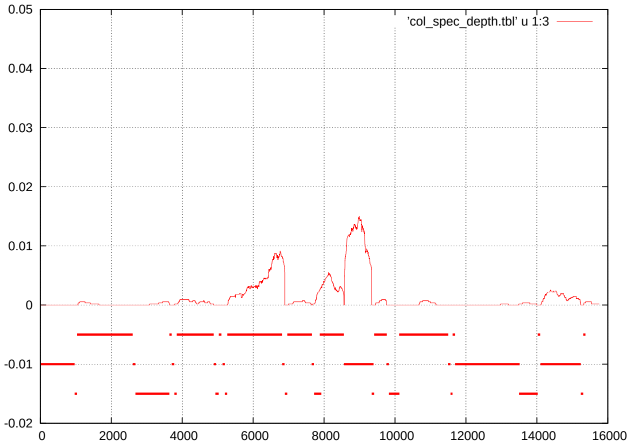
'col_spec_depth.tbl' u 1:3
0.05
0.04
0.03
0.02
0.01
0
-0.01
-0.02
0
2000
4000
6000
8000
10000
12000
14000
16000

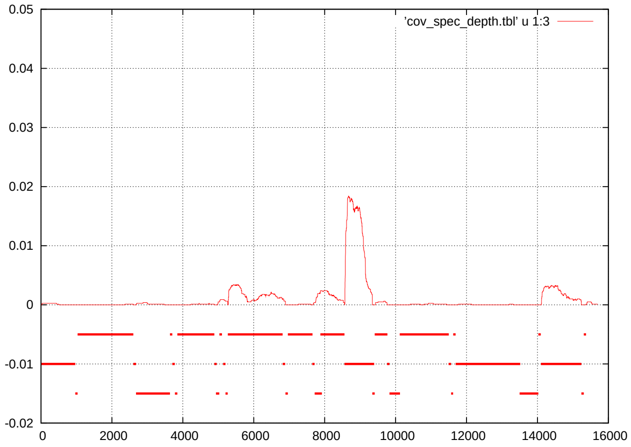
'cov_spec_depth.tbl' u 1:3
0.05
0.04
0.03
0.02
0.01
0
-0.01
-0.02
0
2000
4000
6000
8000
10000
12000
14000
16000

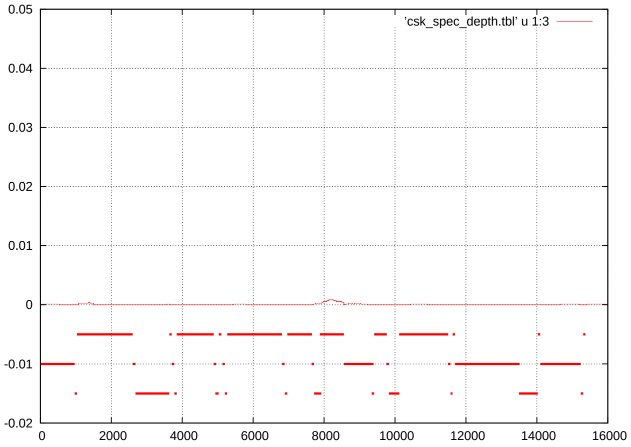
'csk_spec_depth.tbl' u 1:3
0.05
0.04
0.03
0.02
0.01
0
-0.01
-0.02
0
2000
4000
6000
8000
10000
12000
14000
16000

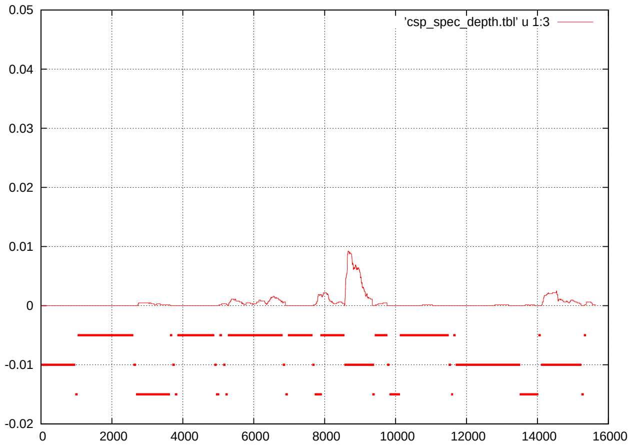
'csp_spec_depth.tbl' u 1:3
0.05
0.04
0.03
0.02
0.01
0
-0.01
-0.02
0 2000 4000 6000 8000 10000 12000 14000 16000

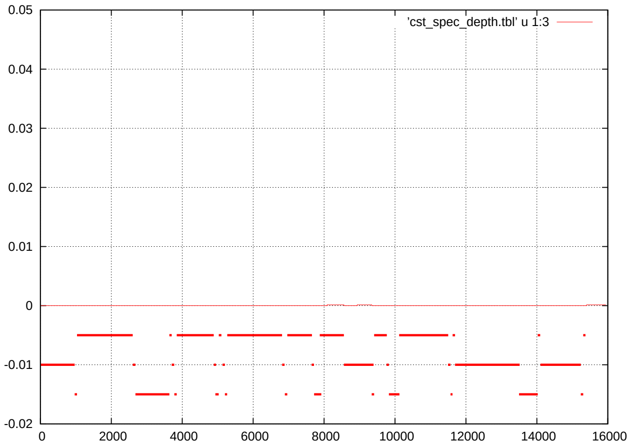
'cst_spec_depth.tbl' u 1:3
0.05
0.04
0.03
0.02
0.01
0
-0.01
-0.02
0 2000 4000 6000 8000 10000 12000 14000 16000

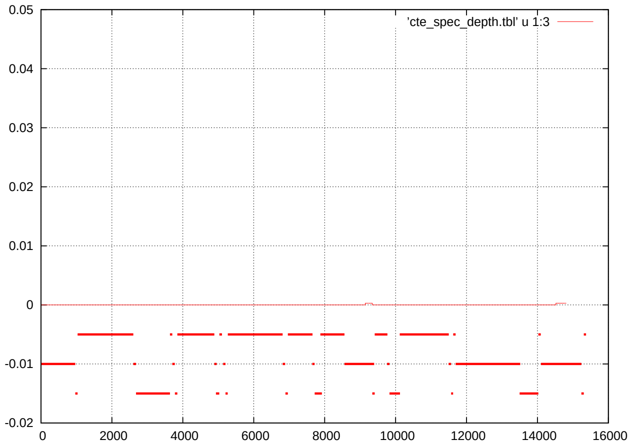
'cte_spec_depth.tbl' u 1:3
0.05
0.04
0.03
0.02
0.01
0
-0.01
-0.02
0 2000 4000 6000 8000 10000 12000 14000 16000

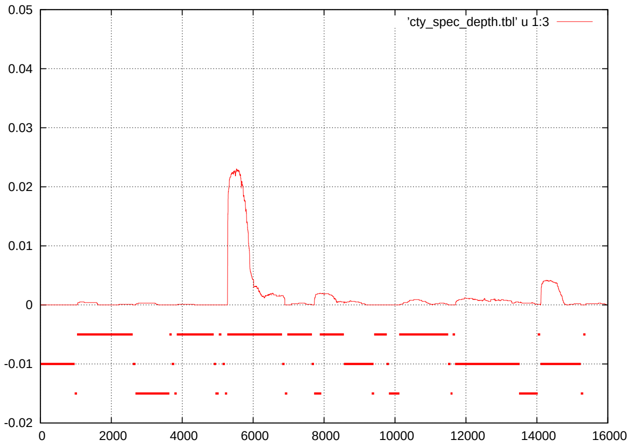
'cty_spec_depth.tbl' u 1:3
0.05
0.04
0.03
0.02
0.01
0
-0.01
-0.02
0 2000 4000 6000 8000 10000 12000 14000 16000

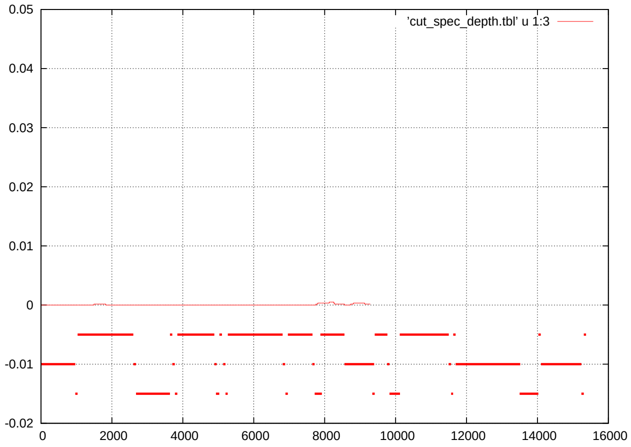
'cut_spec_depth.tbl' u 1:3
0.05
0.04
0.03
0.02
0.01
0
-0.01
-0.02
0 2000 4000 6000 8000 10000 12000 14000 16000

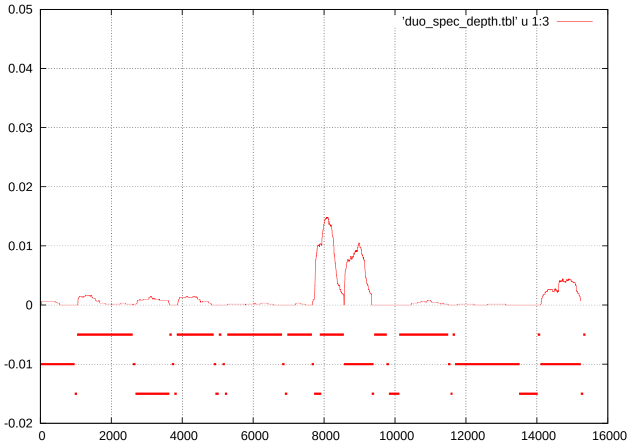
'duo_spec_depth.tbl' u 1:3
0.05
0.04
0.03
0.02
0.01
0
-0.01
-0.02
0 2000 4000 6000 8000 10000 12000 14000 16000

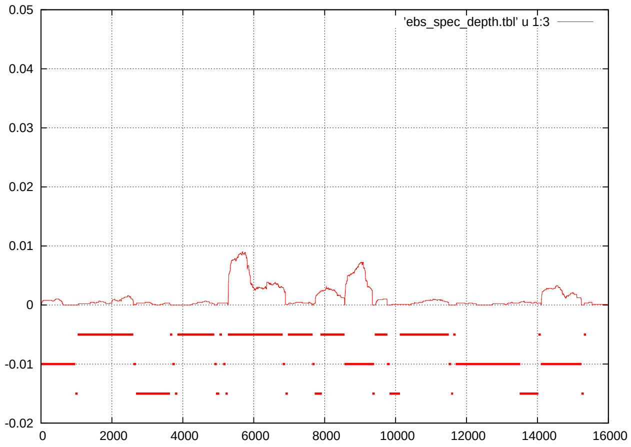
'ebs_spec_depth.tbl' u 1:3
0.05
0.04
0.03
0.02
0.01
0
-0.01
-0.02
0 2000 4000 6000 8000 10000 12000 14000 16000

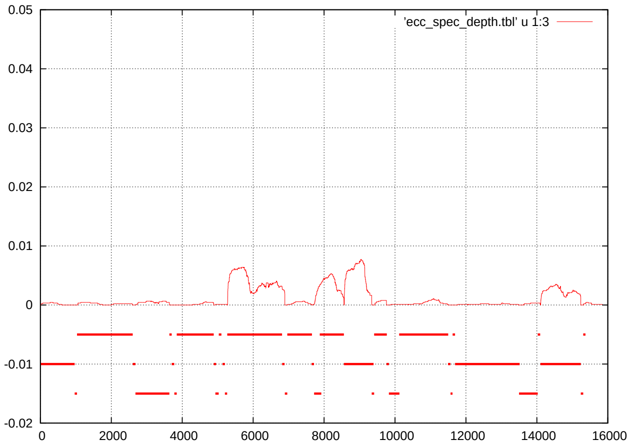
'ecc_spec_depth.tbl' u 1:3
0.05
0.04
0.03
0.02
0.01
0
-0.01
-0.02
0 2000 4000 6000 8000 10000 12000 14000 16000

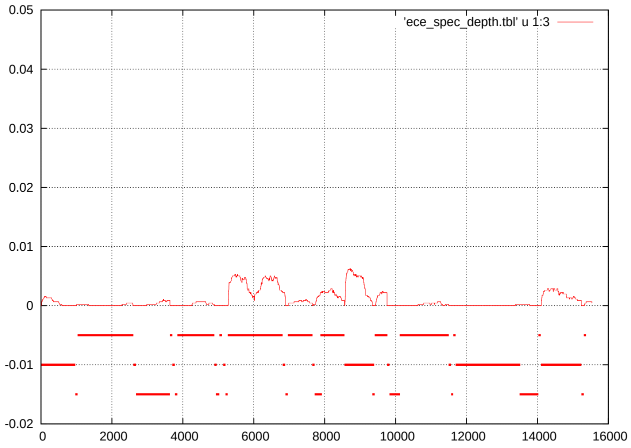
'ece_spec_depth.tbl' u 1:3
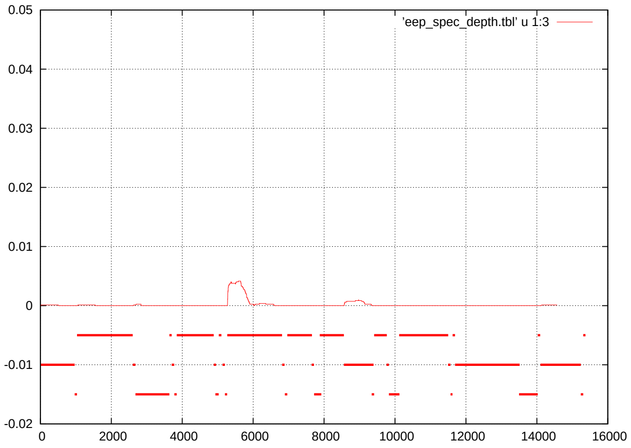
'eep_spec_depth.tbl' u 1:3
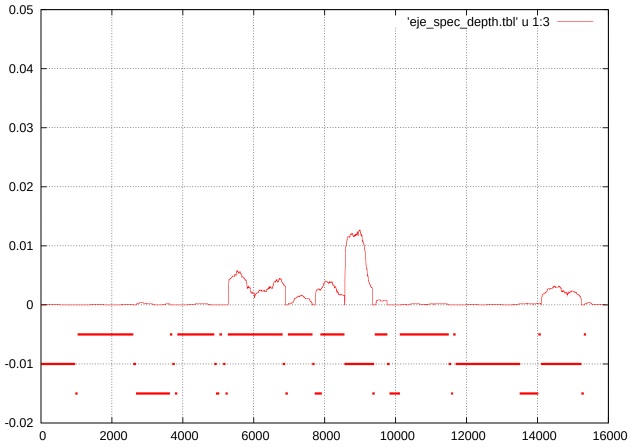
'eje_spec_depth.tbl' u 1:3
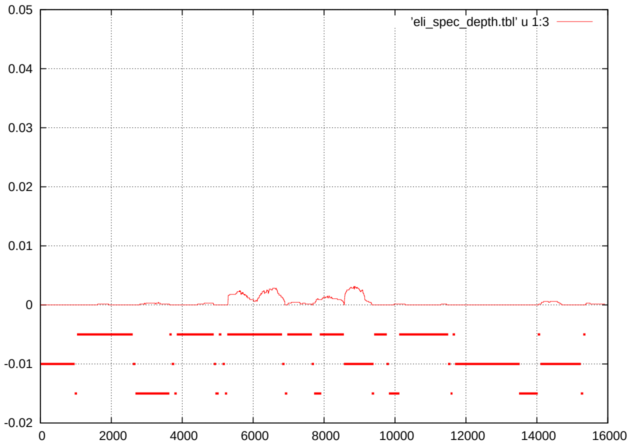
'eli_spec_depth.tbl' u 1:3
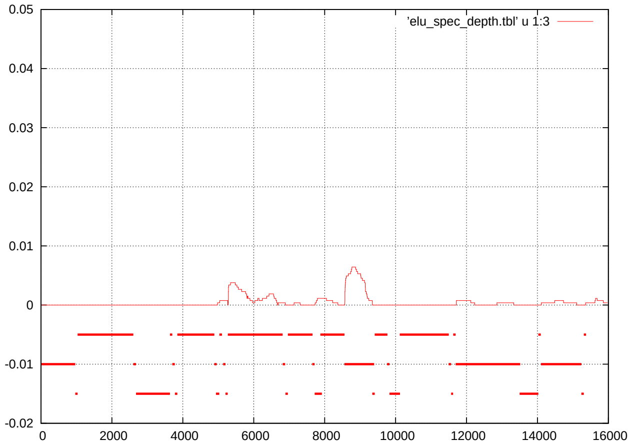
'elu_spec_depth.tbl' u 1:3
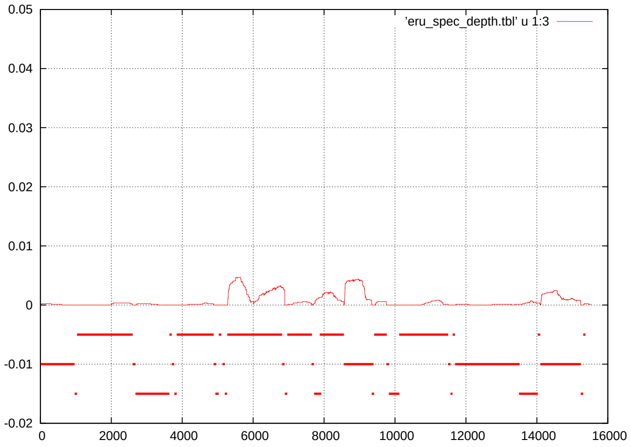
'eru_spec_depth.tbl' u 1:3
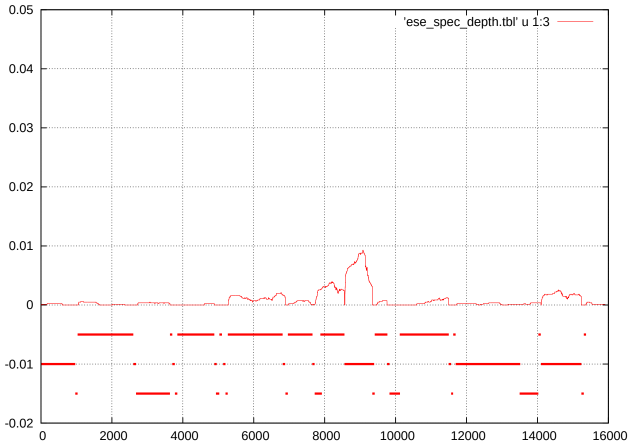
'ese_spec_depth.tbl' u 1:3
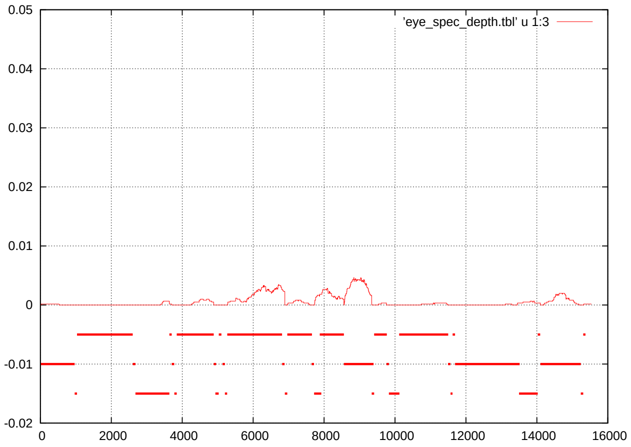
'eye_spec_depth.tbl' u 1:3

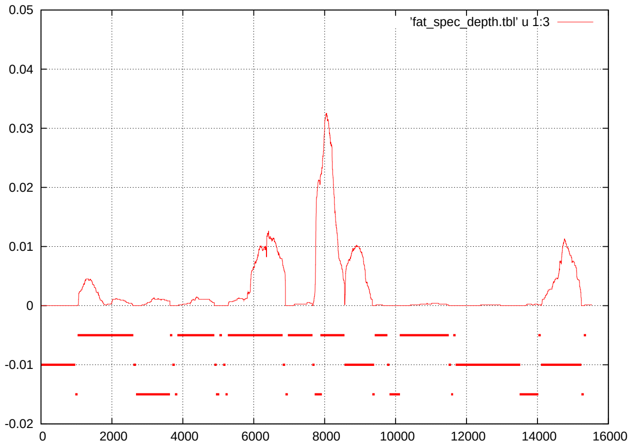
'fat_spec_depth.tbl' u 1:3
0.05
0.04
0.03
0.02
0.01
0
-0.01
-0.02
0 2000 4000 6000 8000 10000 12000 14000 16000

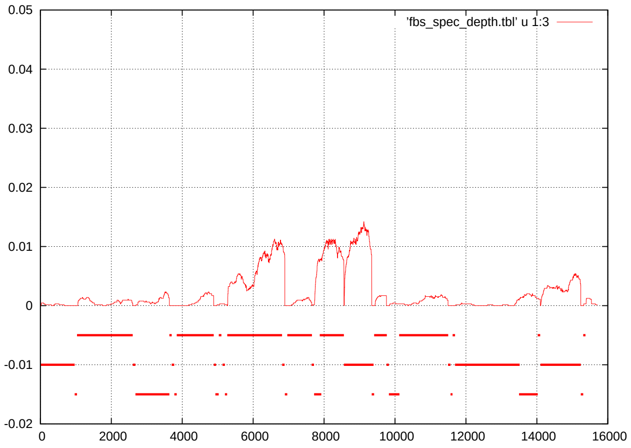
'fbs_spec_depth.tbl' u 1:3
0.05
0.04
0.03
0.02
0.01
0
-0.01
-0.02
0 2000 4000 6000 8000 10000 12000 14000 16000

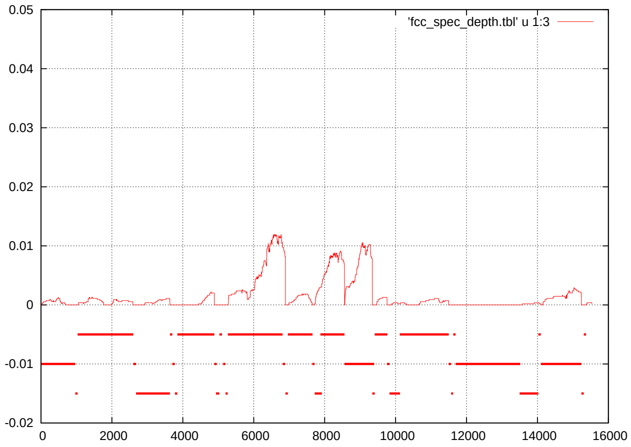
'fcc_spec_depth.tbl' u 1:3
0.05
0.04
0.03
0.02
0.01
0
-0.01
-0.02
0 2000 4000 6000 8000 10000 12000 14000 16000

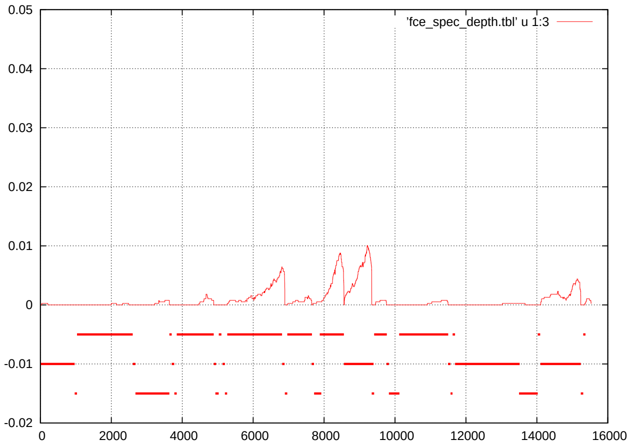
'fce_spec_depth.tbl' u 1:3
0.05
0.04
0.03
0.02
0.01
0
-0.01
-0.02
0 2000 4000 6000 8000 10000 12000 14000 16000

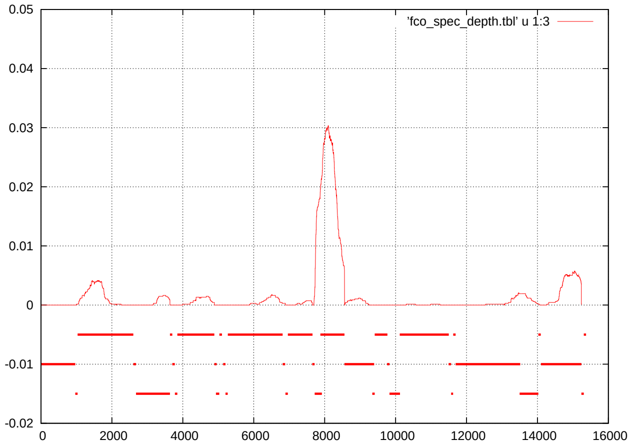
'fco_spec_depth.tbl' u 1:3
0.05
0.04
0.03
0.02
0.01
0
-0.01
-0.02
0 2000 4000 6000 8000 10000 12000 14000 16000

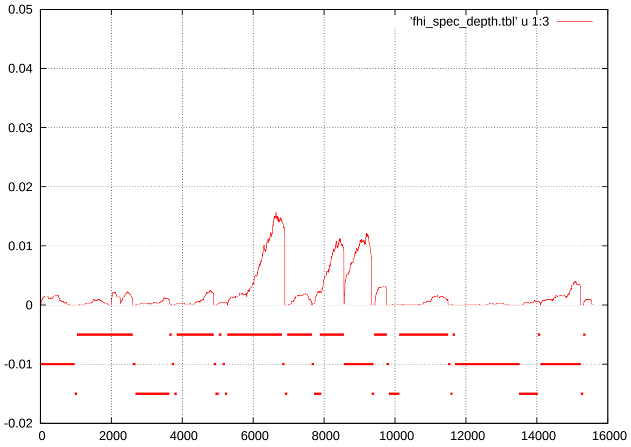
'fhi_spec_depth.tbl' u 1:3
0.05
0.04
0.03
0.02
0.01
0
-0.01
-0.02
0 2000 4000 6000 8000 10000 12000 14000 16000

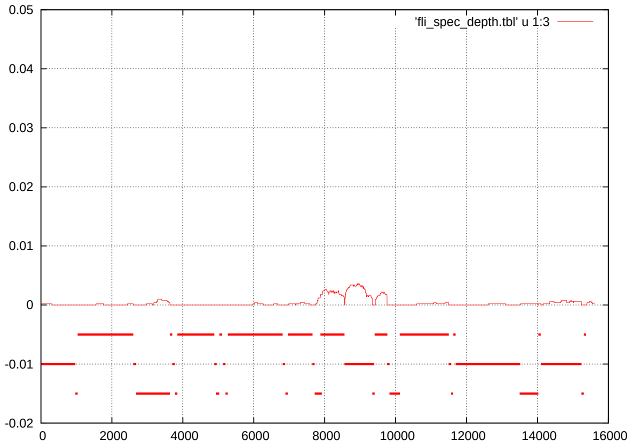
'fli_spec_depth.tbl' u 1:3
0.05
0.04
0.03
0.02
0.01
0
-0.01
-0.02
0 2000 4000 6000 8000 10000 12000 14000 16000

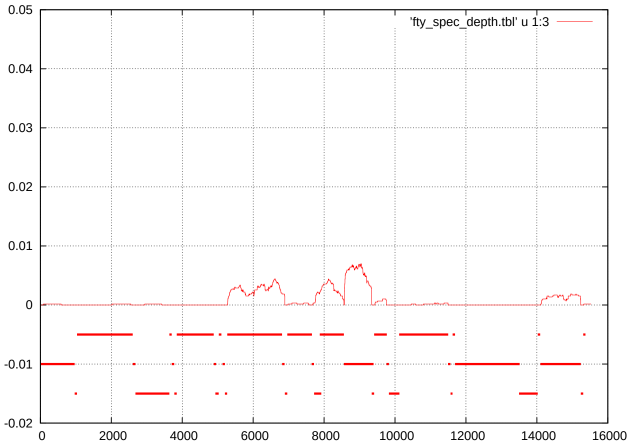
'fty_spec_depth.tbl' u 1:3
0.05
0.04
0.03
0.02
0.01
0
-0.01
-0.02
0 2000 4000 6000 8000 10000 12000 14000 16000

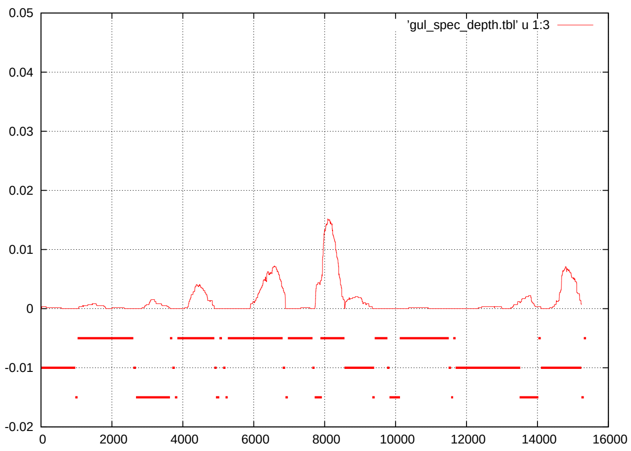
'gul_spec_depth.tbl' u 1:3

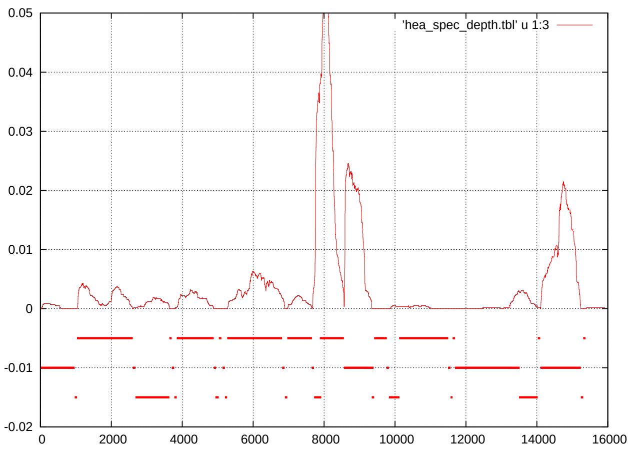
'hea_spec_depth.tbl' u 1:3

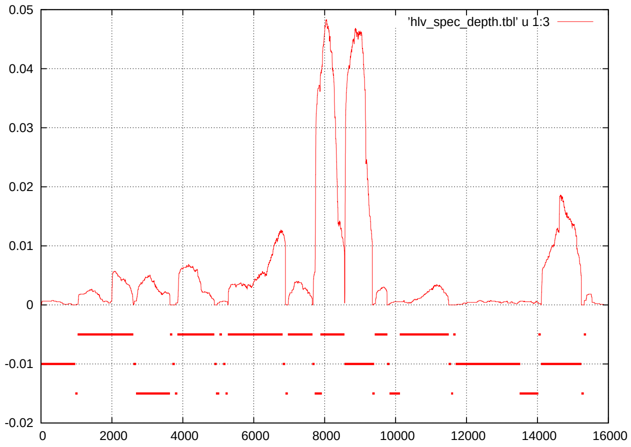
'hlv_spec_depth.tbl' u 1:3

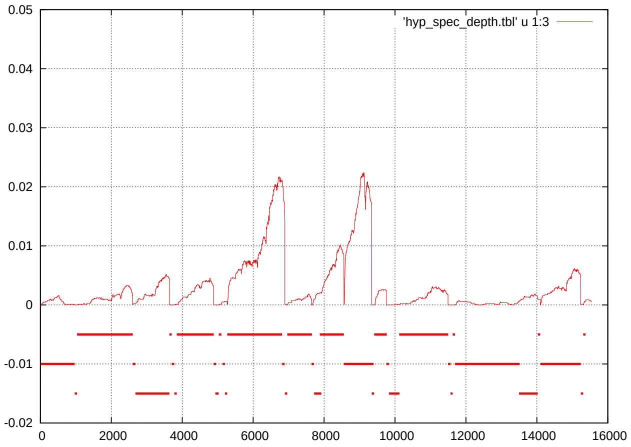
'hyp_spec_depth.tbl' u 1:3

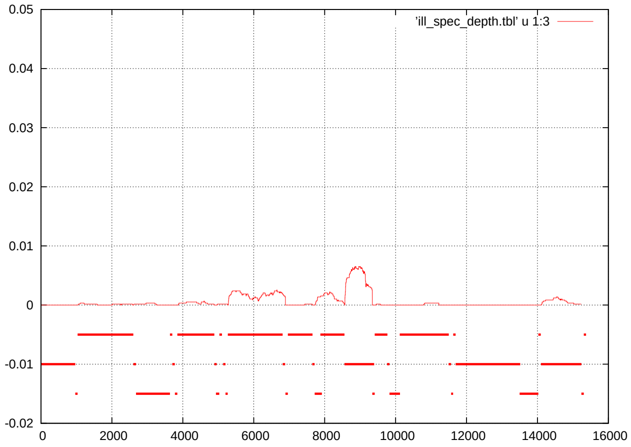
'ill_spec_depth.tbl' u 1:3

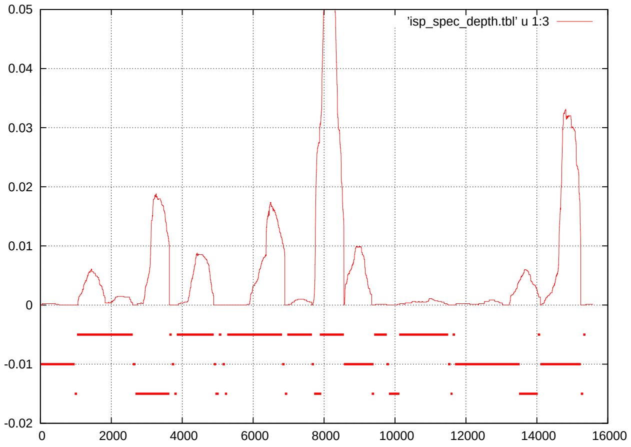
'isp_spec_depth.tbl' u 1:3

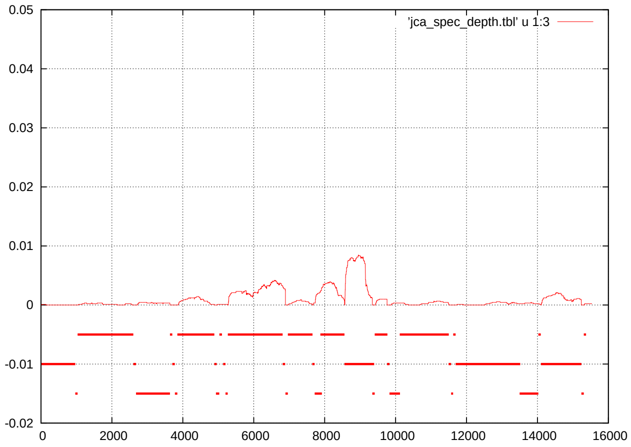
'jca_spec_depth.tbl' u 1:3

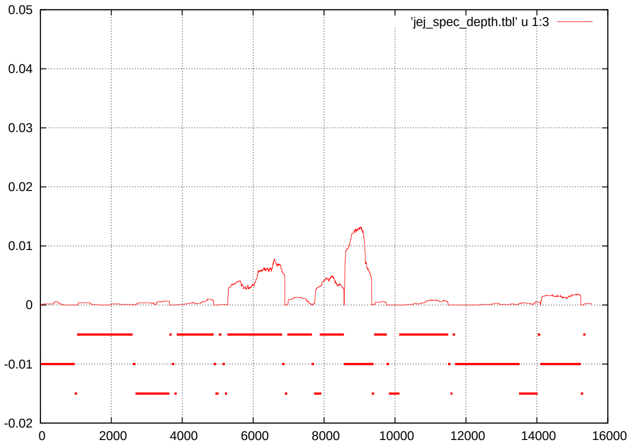
'jej_spec_depth.tbl' u 1:3

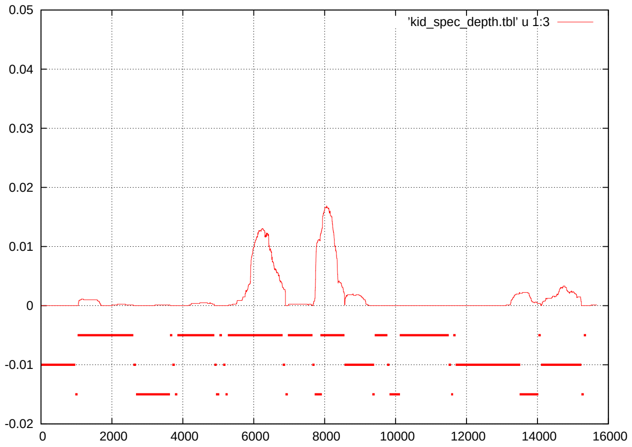
'kid_spec_depth.tbl' u 1:3
0.05
0.04
0.03
0.02
0.01
0
-0.01
-0.02
0
2000
4000
6000
8000
10000
12000
14000
16000

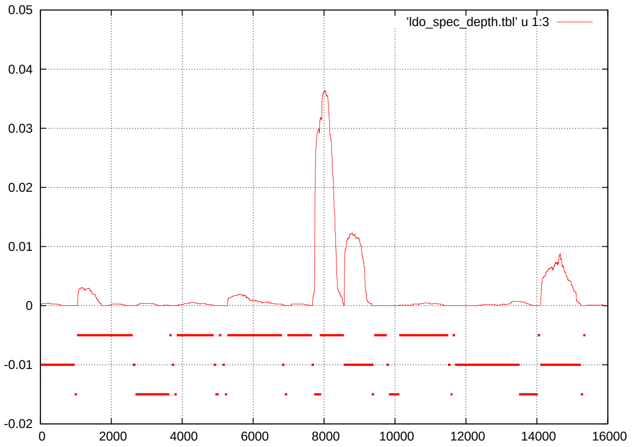
'ldo_spec_depth.tbl' u 1:3
0.05
0.04
0.03
0.02
0.01
0
-0.01
-0.02
0
2000
4000
6000
8000
10000
12000
14000
16000

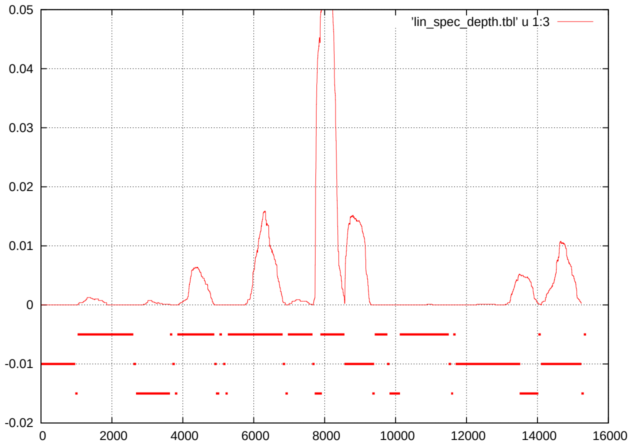
'lin_spec_depth.tbl' u 1:3
0.05
0.04
0.03
0.02
0.01
0
-0.01
-0.02
0
2000
4000
6000
8000
10000
12000
14000
16000

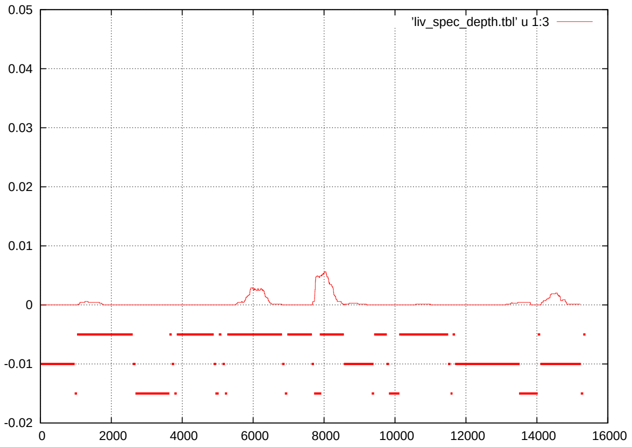
'liv_spec_depth.tbl' u 1:3
0.05
0.04
0.03
0.02
0.01
0
-0.01
-0.02
0
2000
4000
6000
8000
10000
12000
14000
16000

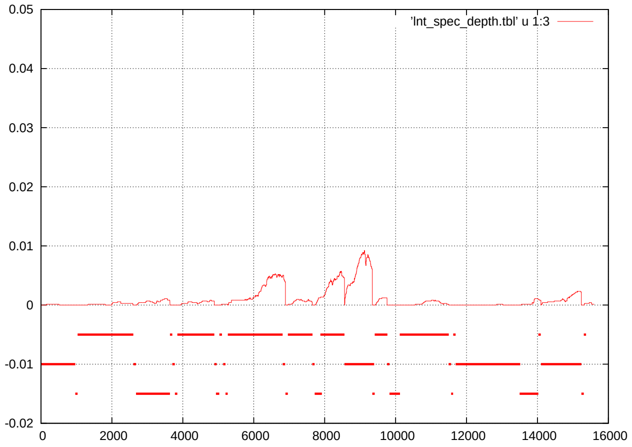
'lnt_spec_depth.tbl' u 1:3
0.05
0.04
0.03
0.02
0.01
0
-0.01
-0.02
0
2000
4000
6000
8000
10000
12000
14000
16000

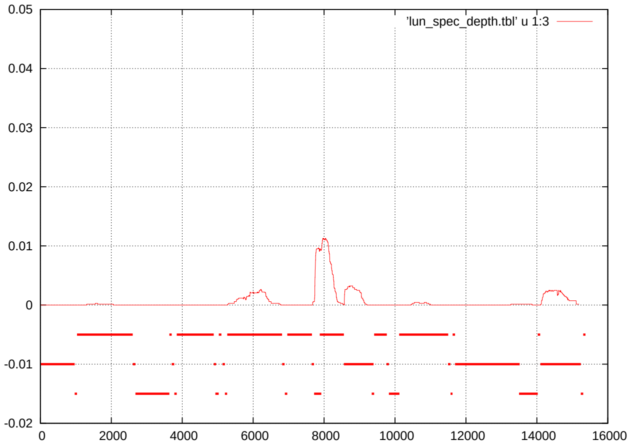
'lun_spec_depth.tbl' u 1:3
0.05
0.04
0.03
0.02
0.01
0
-0.01
-0.02
0
2000
4000
6000
8000
10000
12000
14000
16000

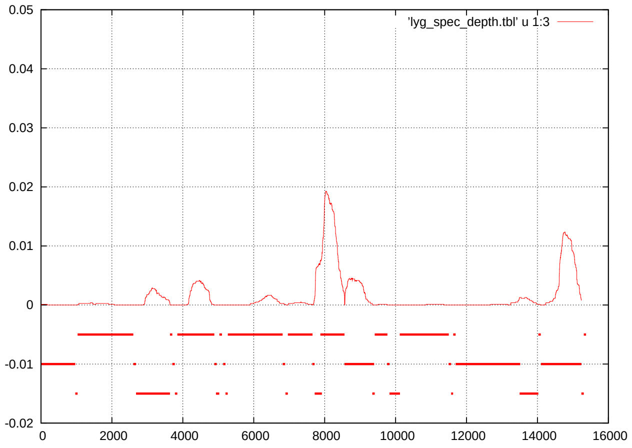
'lyg_spec_depth.tbl' u 1:3
0.05
0.04
0.03
0.02
0.01
0
-0.01
-0.02
0
2000
4000
6000
8000
10000
12000
14000
16000

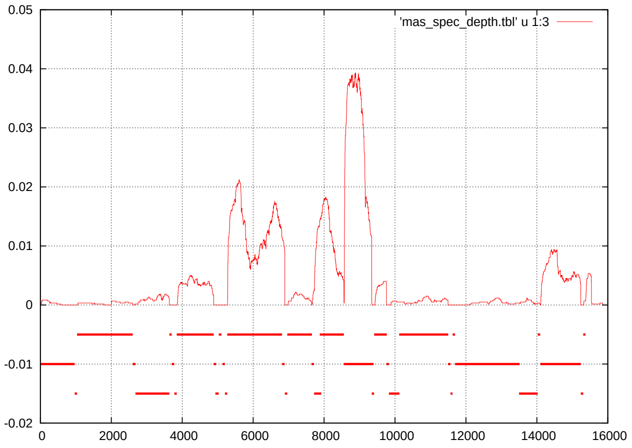
'mas_spec_depth.tbl' u 1:3
0.05
0.04
0.03
0.02
0.01
0
-0.01
-0.02
0
2000
4000
6000
8000
10000
12000
14000
16000

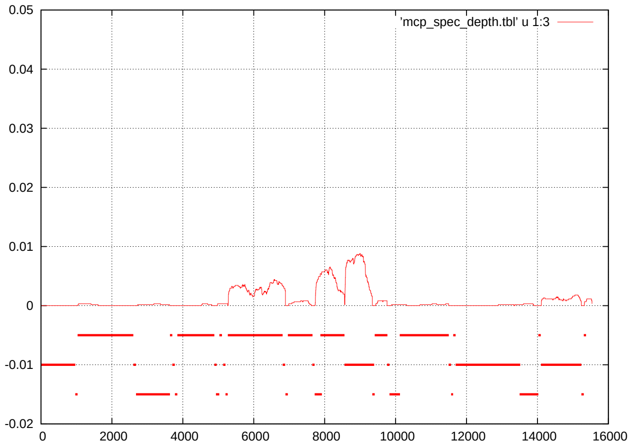
'mcp_spec_depth.tbl' u 1:3

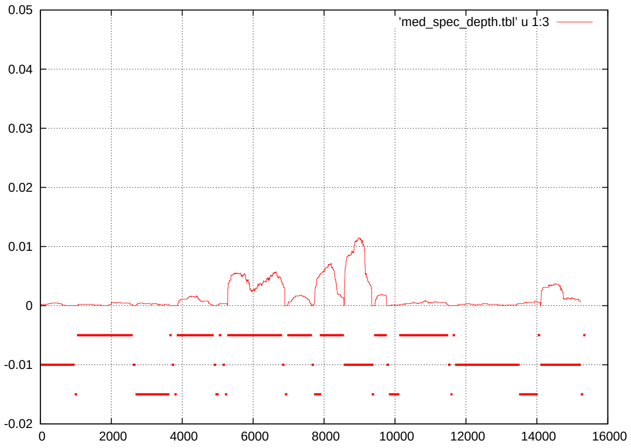
'med_spec_depth.tbl' u 1:3

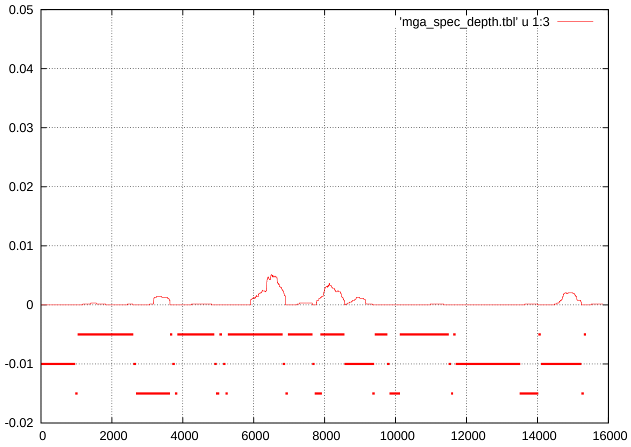
'mga_spec_depth.tbl' u 1:3

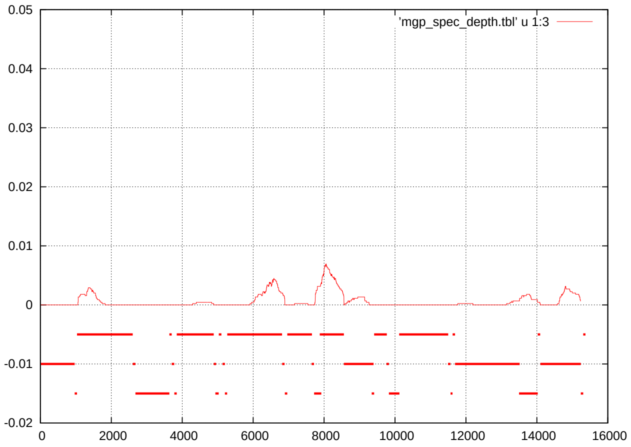
'mgp_spec_depth.tbl' u 1:3

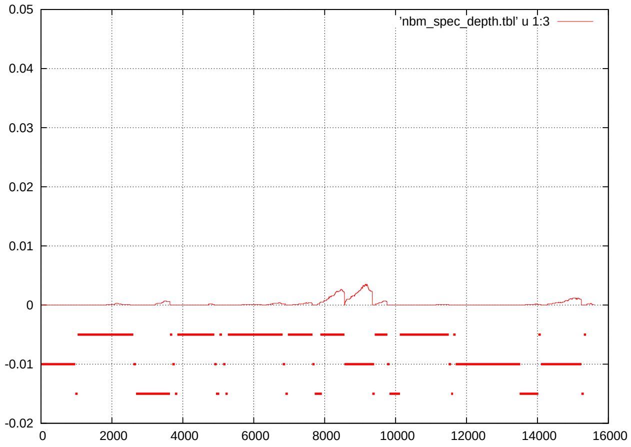
'nbm_spec_depth.tbl' u 1:3

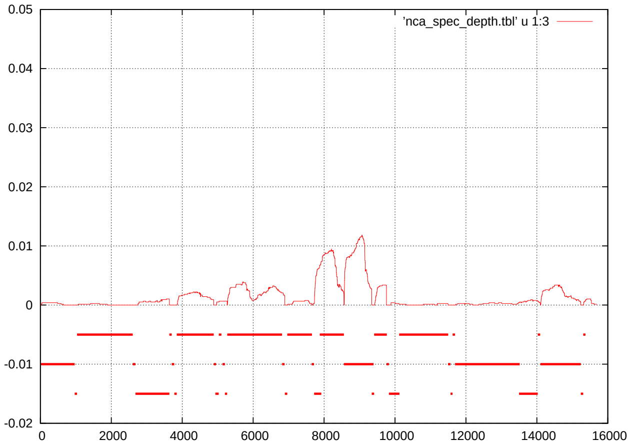
'nca_spec_depth.tbl' u 1:3

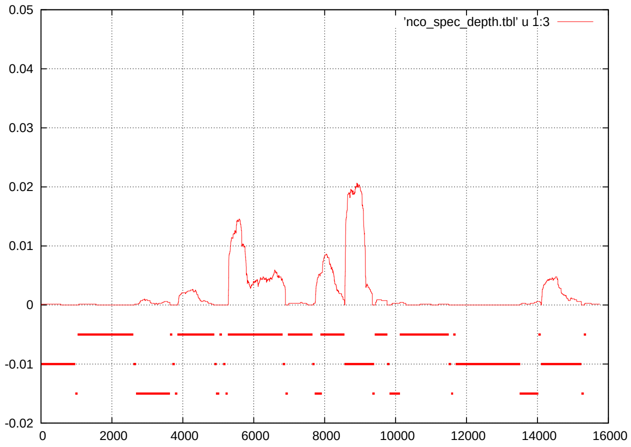
'nco_spec_depth.tbl' u 1:3

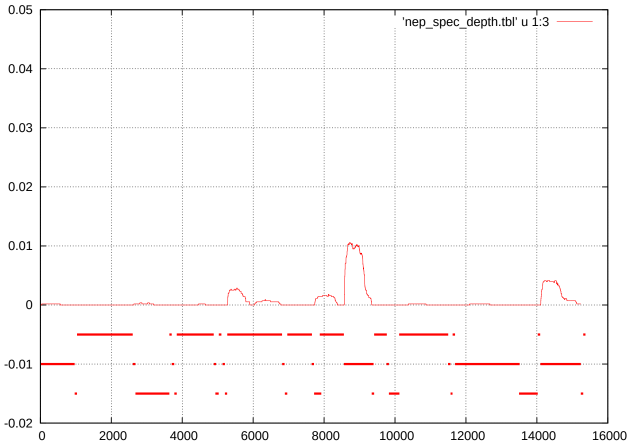
'nep_spec_depth.tbl' u 1:3

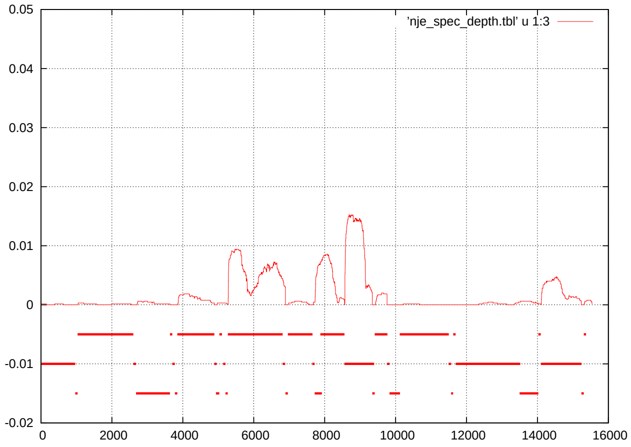
'nje_spec_depth.tbl' u 1:3

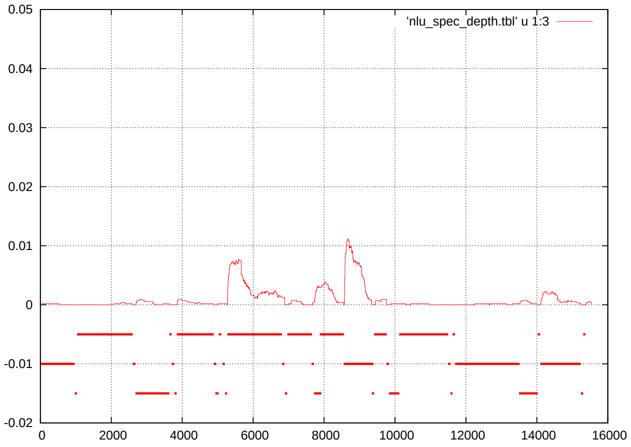
'nlu_spec_depth.tbl' u 1:3

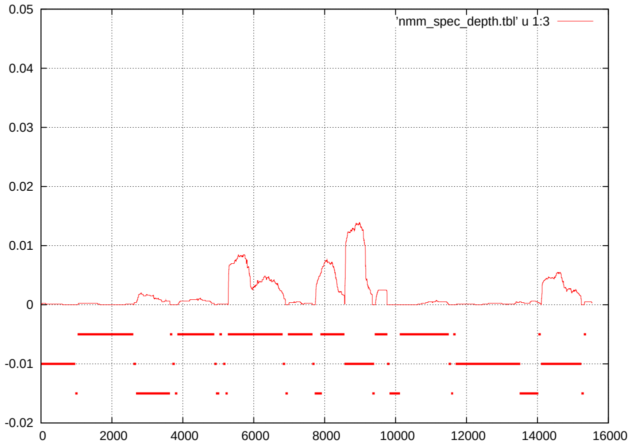
'nmm_spec_depth.tbl' u 1:3

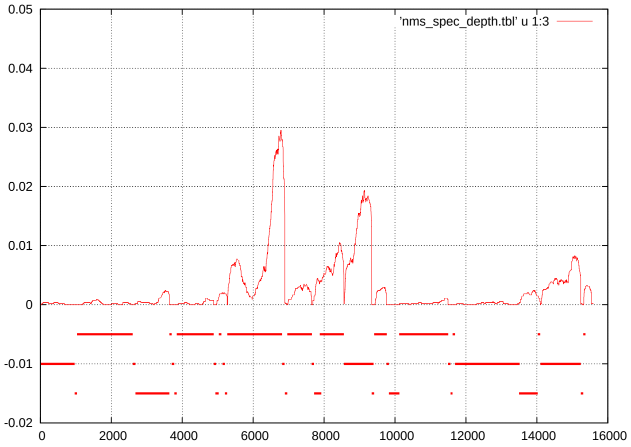
'nms_spec_depth.tbl' u 1:3

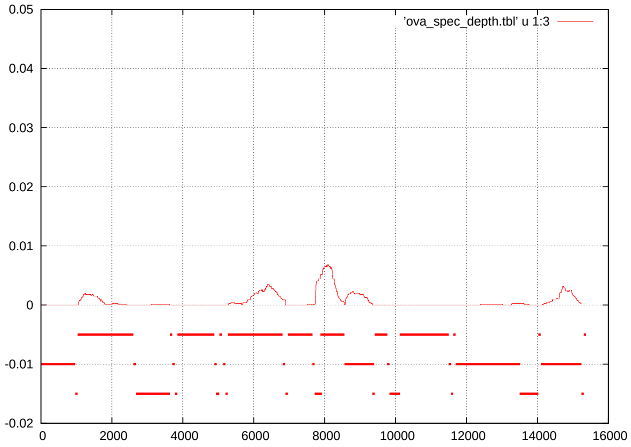
'ova_spec_depth.tbl' u 1:3

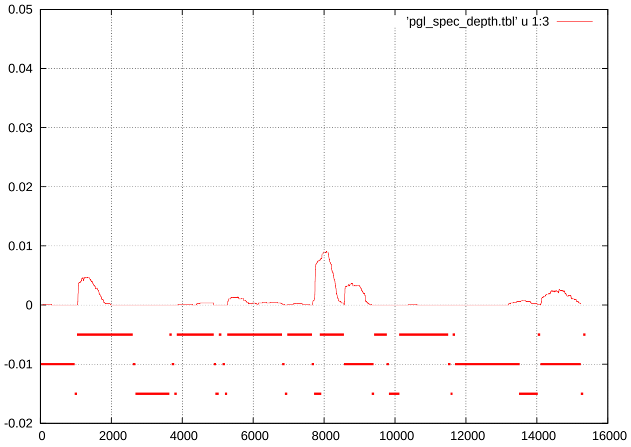
'pgl_spec_depth.tbl' u 1:3

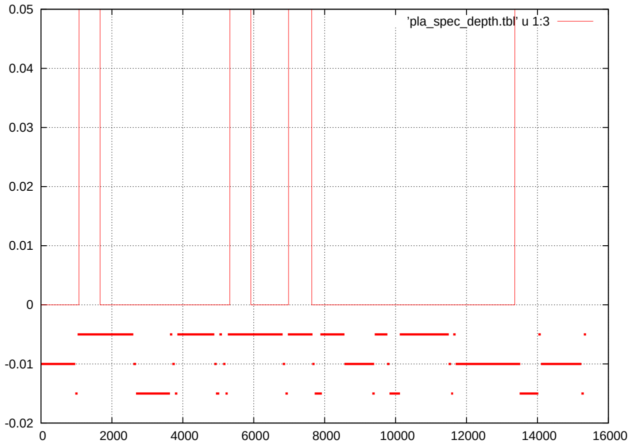
'pla_spec_depth.tbl' u 1:3

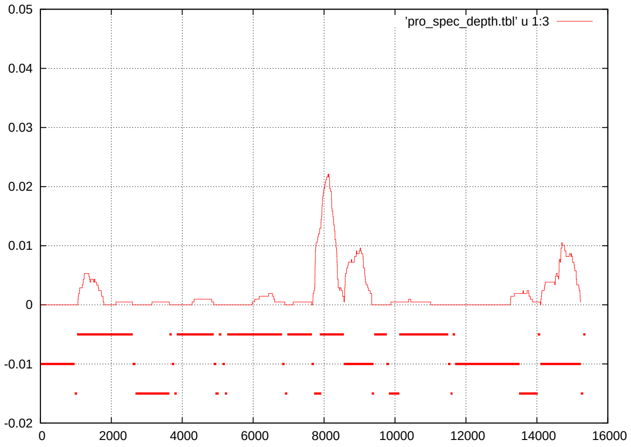
'pro_spec_depth.tbl' u 1:3

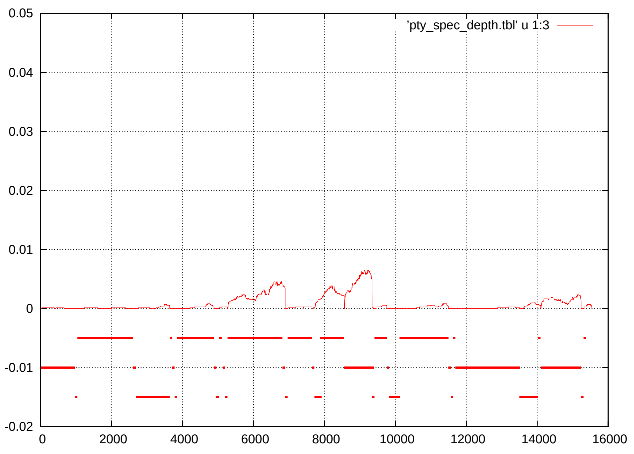
'pty_spec_depth.tbl' u 1:3
0.05 0.04 0.03 0.02 0.01 0 -0.01 -0.02
0 2000 4000 6000 8000 10000 12000 14000 16000

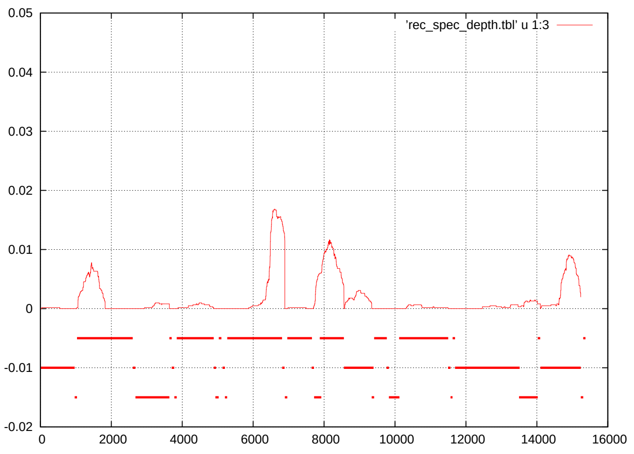
'rec_spec_depth.tbl' u 1:3
0.05 0.04 0.03 0.02 0.01 0 -0.01 -0.02
0 2000 4000 6000 8000 10000 12000 14000 16000

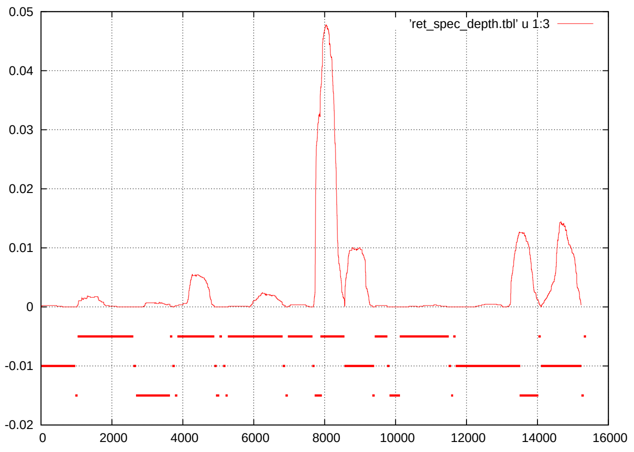
'ret_spec_depth.tbl' u 1:3
0.05 0.04 0.03 0.02 0.01 0 -0.01 -0.02
0 2000 4000 6000 8000 10000 12000 14000 16000

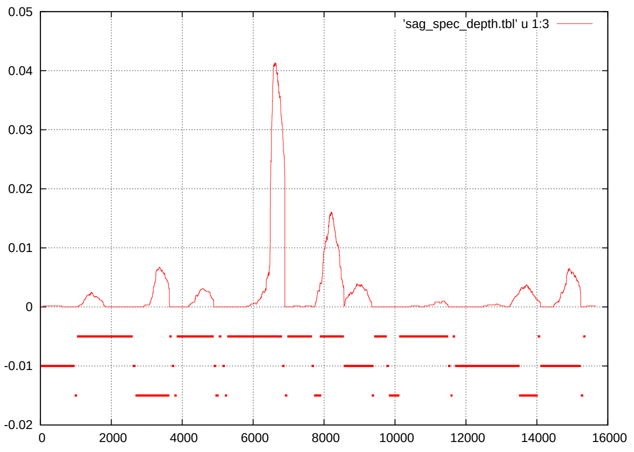
'sag_spec_depth.tbl' u 1:3
0.05 0.04 0.03 0.02 0.01 0 -0.01 -0.02
0 2000 4000 6000 8000 10000 12000 14000 16000

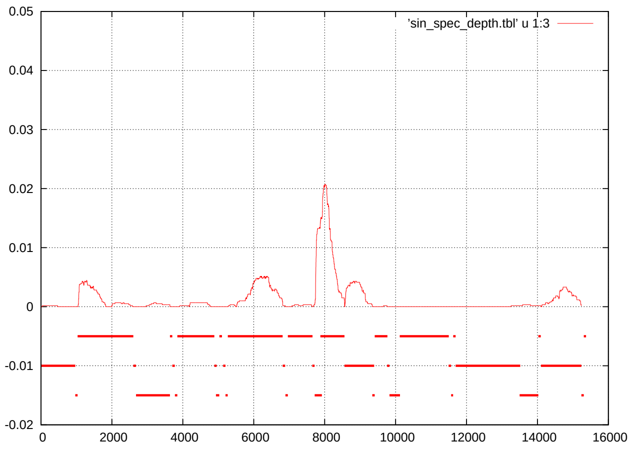
'sin_spec_depth.tbl' u 1:3
0.05 0.04 0.03 0.02 0.01 0 -0.01 -0.02
0 2000 4000 6000 8000 10000 12000 14000 16000

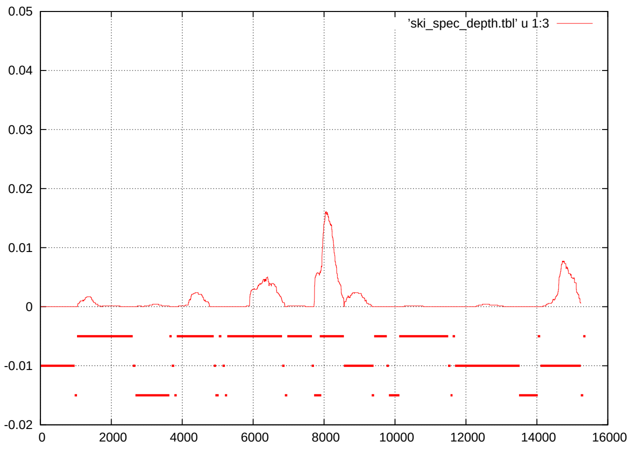
'ski_spec_depth.tbl' u 1:3
0.05 0.04 0.03 0.02 0.01 0 -0.01 -0.02
0 2000 4000 6000 8000 10000 12000 14000 16000

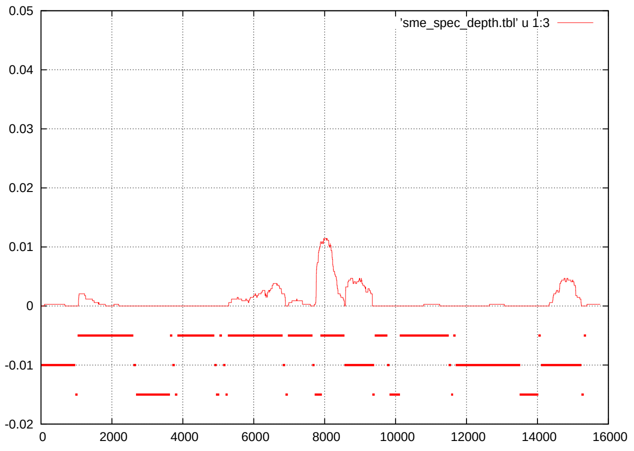
'sme_spec_depth.tbl' u 1:3
0.05 0.04 0.03 0.02 0.01 0 -0.01 -0.02
0 2000 4000 6000 8000 10000 12000 14000 16000

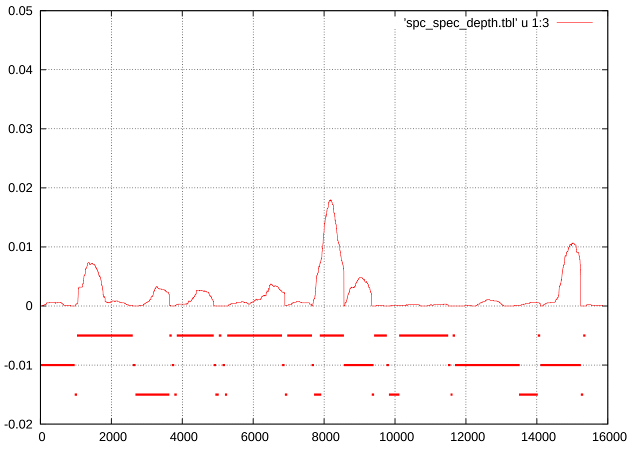
'spc_spec_depth.tbl' u 1:3
0.05 0.04 0.03 0.02 0.01 0 -0.01 -0.02
0 2000 4000 6000 8000 10000 12000 14000 16000

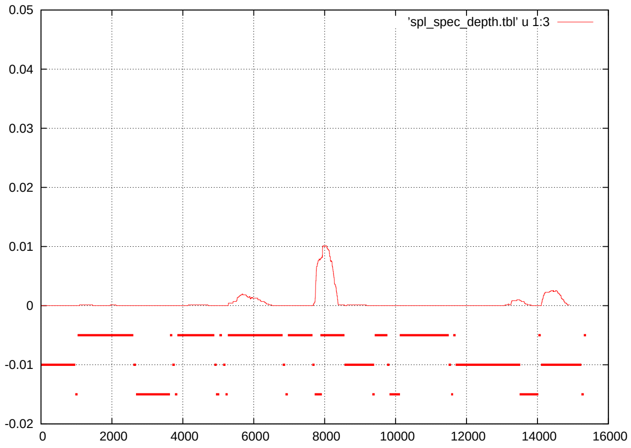
'spl_spec_depth.tbl' u 1:3
0.05
0.04
0.03
0.02
0.01
0
-0.01
-0.02
0 2000 4000 6000 8000 10000 12000 14000 16000

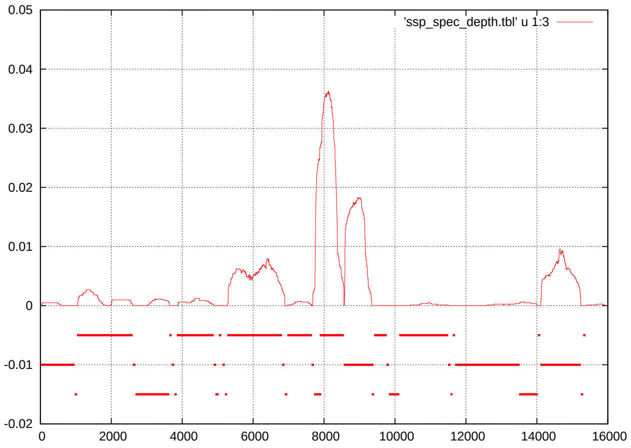
'ssp_spec_depth.tbl' u 1:3
0.05
0.04
0.03
0.02
0.01
0
-0.01
-0.02
0 2000 4000 6000 8000 10000 12000 14000 16000

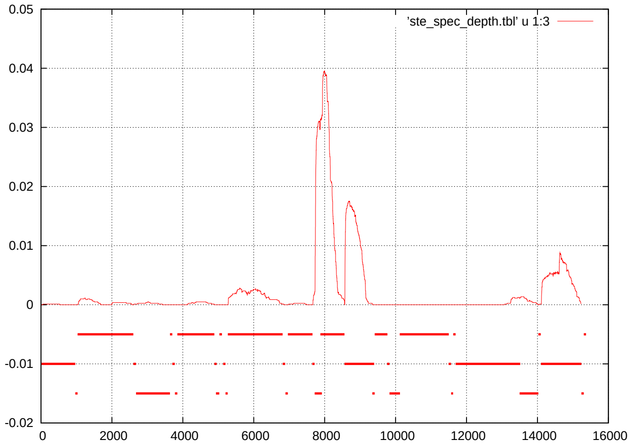
'ste_spec_depth.tbl' u 1:3
0.05
0.04
0.03
0.02
0.01
0
-0.01
-0.02
0 2000 4000 6000 8000 10000 12000 14000 16000

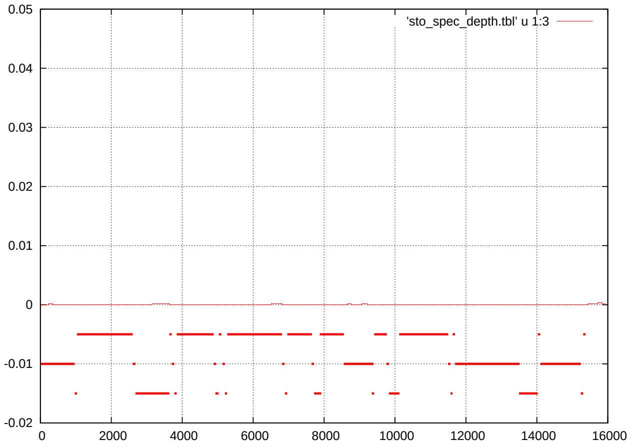
'sto_spec_depth.tbl' u 1:3
0.05
0.04
0.03
0.02
0.01
0
-0.01
-0.02
0 2000 4000 6000 8000 10000 12000 14000 16000

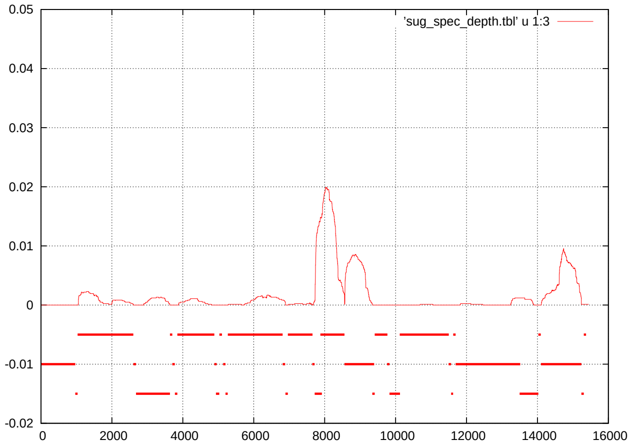
'sug_spec_depth.tbl' u 1:3
0.05
0.04
0.03
0.02
0.01
0
-0.01
-0.02
0 2000 4000 6000 8000 10000 12000 14000 16000

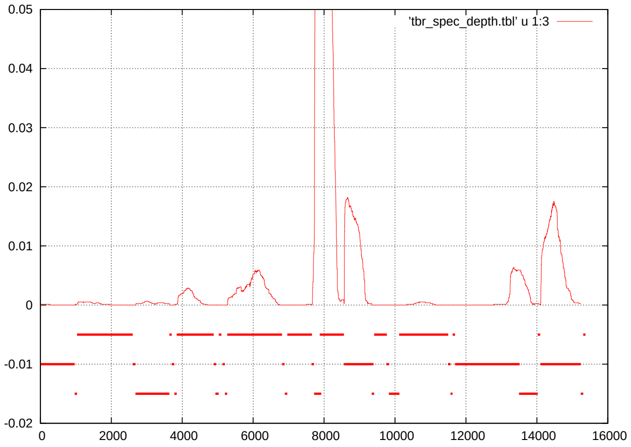
'tbr_spec_depth.tbl' u 1:3
0.05
0.04
0.03
0.02
0.01
0
-0.01
-0.02
0 2000 4000 6000 8000 10000 12000 14000 16000

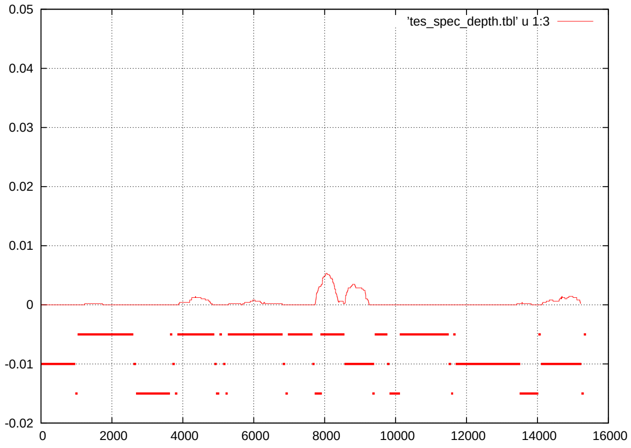
'tes_spec_depth.tbl' u 1:3
0.05
0.04
0.03
0.02
0.01
0
-0.01
-0.02
0 2000 4000 6000 8000 10000 12000 14000 16000

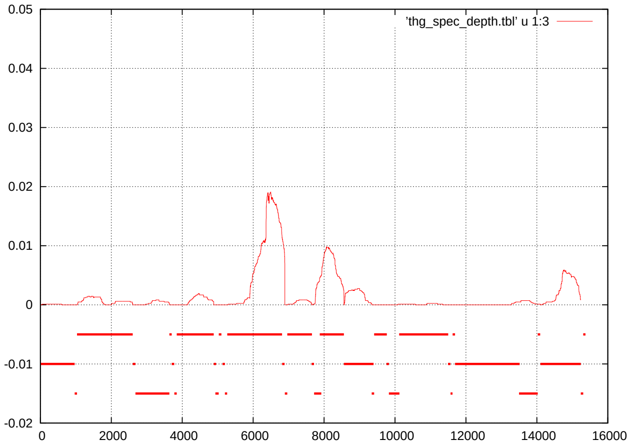
'thg_spec_depth.tbl' u 1:3
0.05
0.04
0.03
0.02
0.01
0
-0.01
-0.02
0 2000 4000 6000 8000 10000 12000 14000 16000

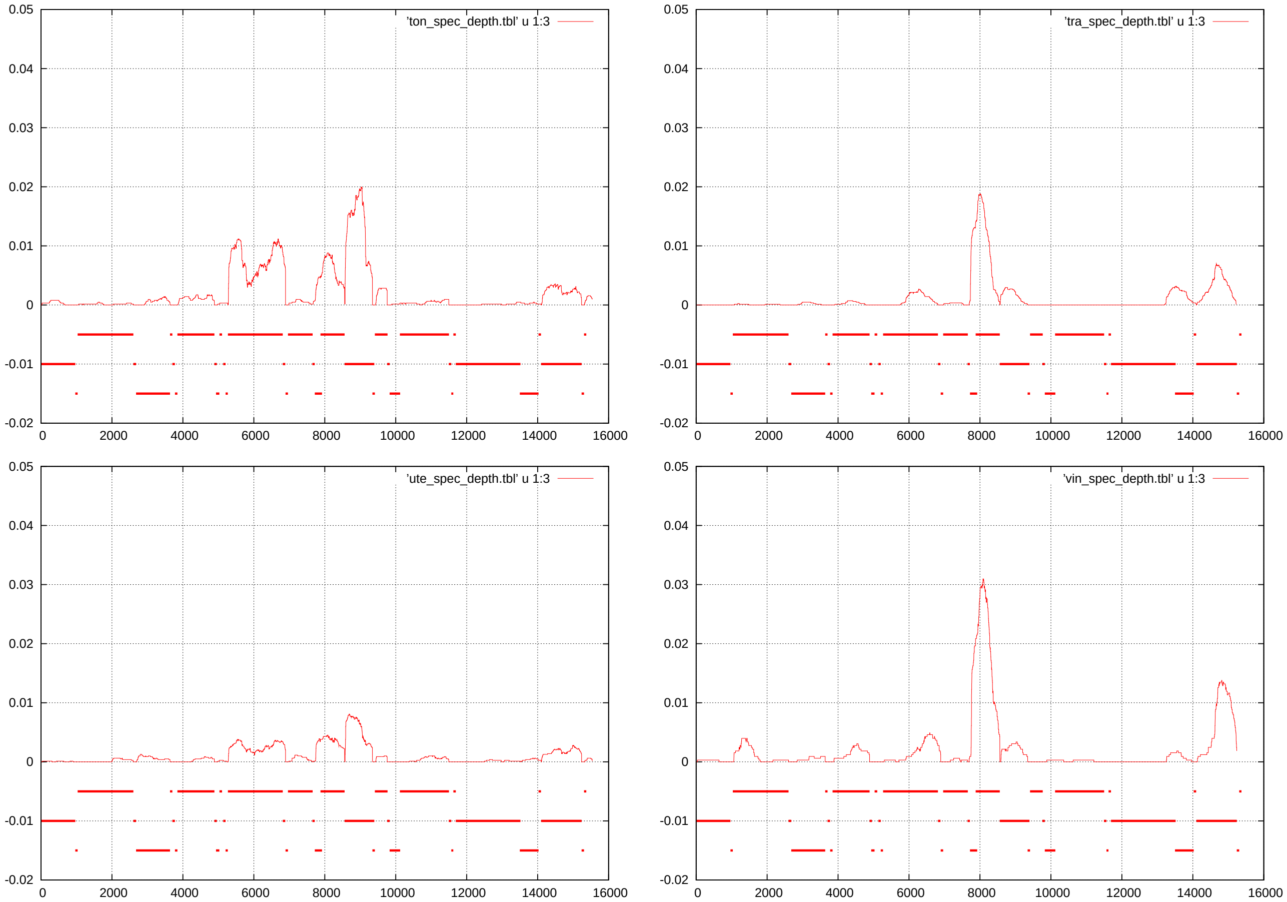

'ton_spec_depth.tbl' u 1:3
'tra_spec_depth.tbl' u 1:3
'ute_spec_depth.tbl' u 1:3
'vin_spec_depth.tbl' u 1:3
0.05
0.04
0.03
0.02
0.01
0
-0.01
-0.02
0
2000
4000
6000
8000
10000
12000
14000
16000
