# Supplementary material for: EST analysis on pig mitochondria reveal novel expression differences between developmental and adult tissues
Source: BMC Genomics. 2007 Oct 11;8:367. doi: 10.1186/1471-2164-8-367 (PMC2194790; doi:10.1186/1471-2164-8-367)
Supplement: Additional file 3 — Normalized COXI coverage from libraries. The expression profile of the COX I gene for each cDN A library. [file 1471-2164-8-367-S3.pdf]

Additional Data File 3:
EST analysis on pig mitochondria reveal novel expression differences between developmental and adult tissues

Karsten Scheibye-Knudsen[1], Susanna Cirera[1], Michael J. Gilchrist[2], Merete Fredholm[1],
Jan Gorodkin*[1]

[1]Division of Genetics and Bioinformatics, IBHV, University of Copenhagen, Grnnegrdsvej 3, DK-1870 Frederiksberg, Denmark
[2]The Wellcome Trust/Cancer Research UK Gurdon Institute, Cambridge, CB2 1QN, UK

Email: Karsten Scheibye-Knudsen - scheibye@genome.ku.dk; Susanna Cirera - scs@life.ku.dk; Michael J. Gilchrist - m.gilchrist@gurdon.cam.ac.uk; Merete Fredholm - mf@life.ku.dk; Jan Gorodkin*- gorodkin@genome.ku.dk;

*Corresponding author

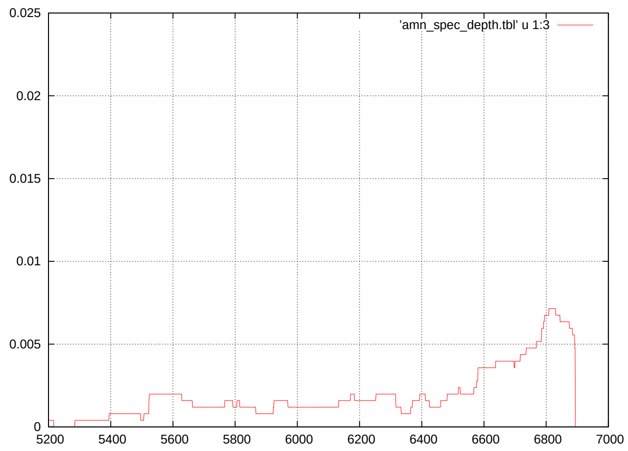
'amn_spec_depth.tbl' u 1:3
0.025
0.02
0.015
0.01
0.005
0
5200 5400 5600 5800 6000 6200 6400 6600 6800 7000

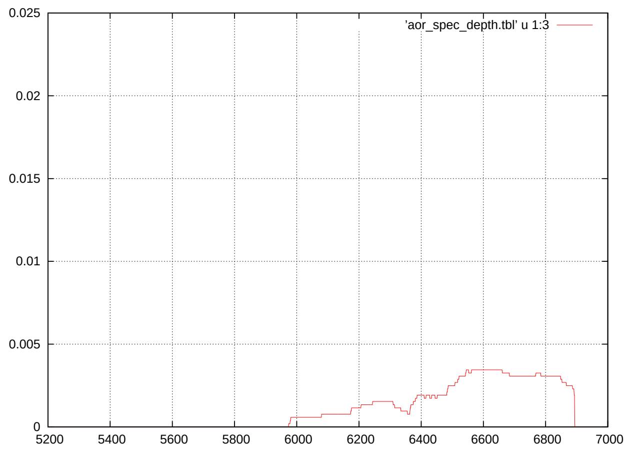
'aor_spec_depth.tbl' u 1:3
0.025
0.02
0.015
0.01
0.005
0
5200 5400 5600 5800 6000 6200 6400 6600 6800 7000

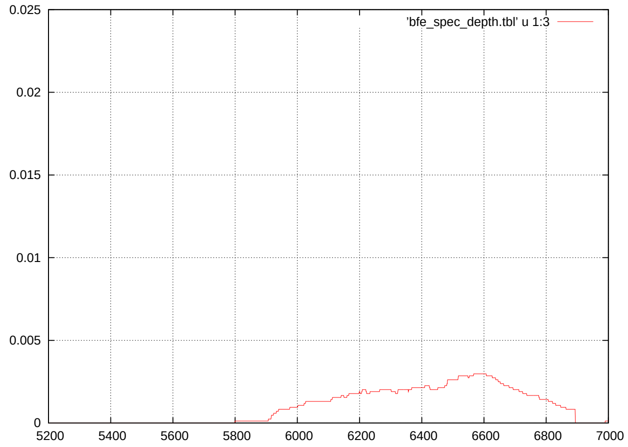
'bfe_spec_depth.tbl' u 1:3
0.025
0.02
0.015
0.01
0.005
0
5200 5400 5600 5800 6000 6200 6400 6600 6800 7000

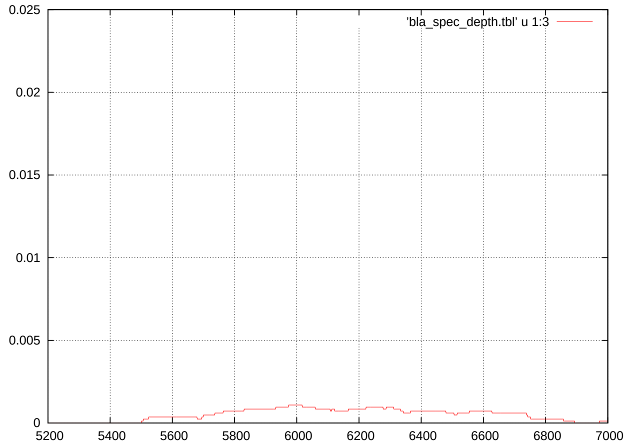
'bla_spec_depth.tbl' u 1:3
0.025
0.02
0.015
0.01
0.005
0
5200 5400 5600 5800 6000 6200 6400 6600 6800 7000

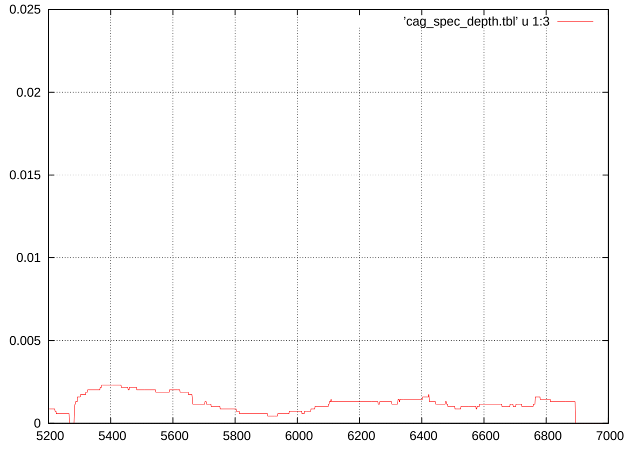
'cag_spec_depth.tbl' u 1:3
0.025
0.02
0.015
0.01
0.005
0
5200 5400 5600 5800 6000 6200 6400 6600 6800 7000

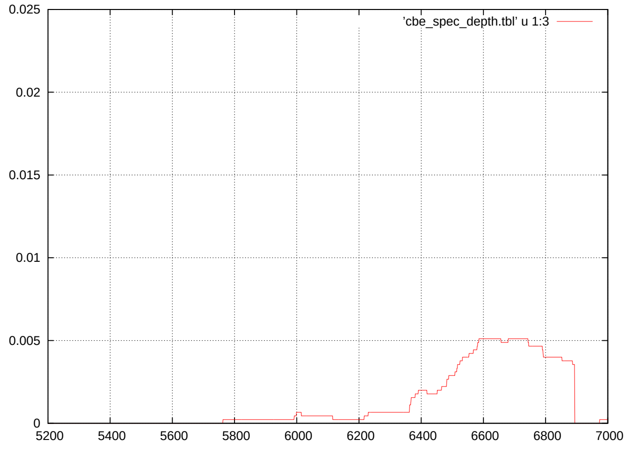
'cbe_spec_depth.tbl' u 1:3
0.025
0.02
0.015
0.01
0.005
0
5200 5400 5600 5800 6000 6200 6400 6600 6800 7000

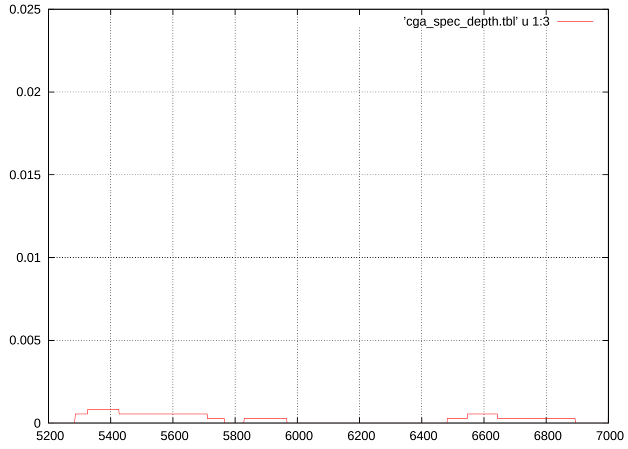
'cga_spec_depth.tbl' u 1:3
0.025
0.02
0.015
0.01
0.005
0
5200 5400 5600 5800 6000 6200 6400 6600 6800 7000

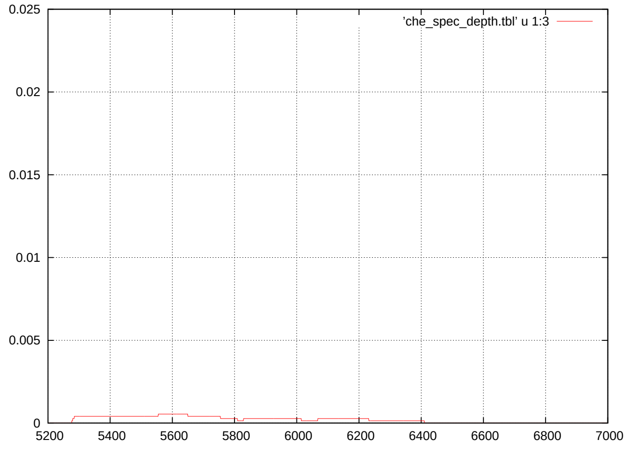
'che_spec_depth.tbl' u 1:3
0.025
0.02
0.015
0.01
0.005
0
5200 5400 5600 5800 6000 6200 6400 6600 6800 7000

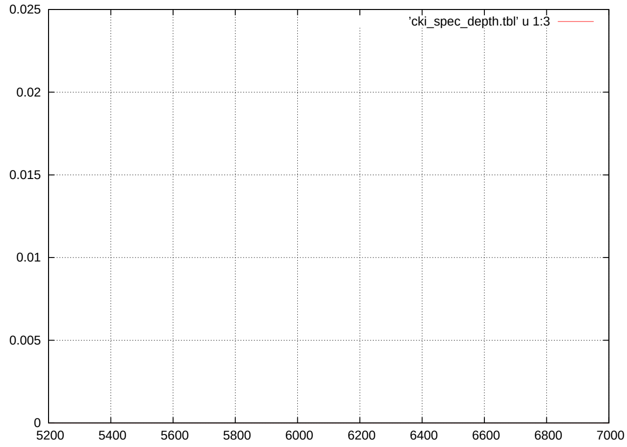
'cki_spec_depth.tbl' u 1:3
0.025
0.02
0.015
0.01
0.005
0
5200 5400 5600 5800 6000 6200 6400 6600 6800 7000

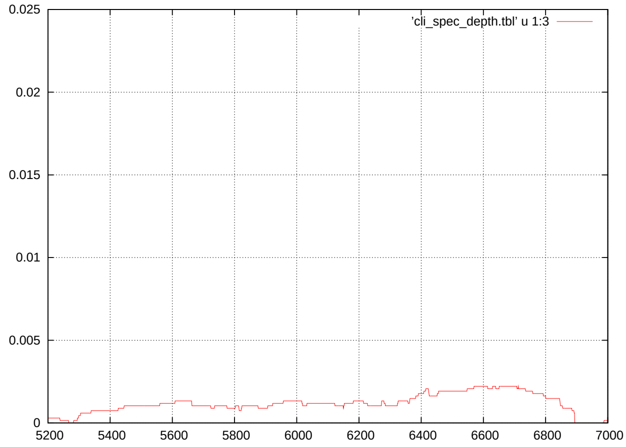
'cli_spec_depth.tbl' u 1:3
0.025
0.02
0.015
0.01
0.005
0
5200 5400 5600 5800 6000 6200 6400 6600 6800 7000

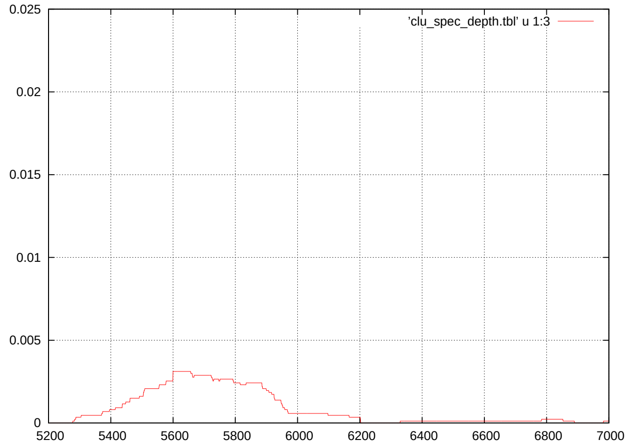
'clu_spec_depth.tbl' u 1:3
0.025
0.02
0.015
0.01
0.005
0
5200 5400 5600 5800 6000 6200 6400 6600 6800 7000

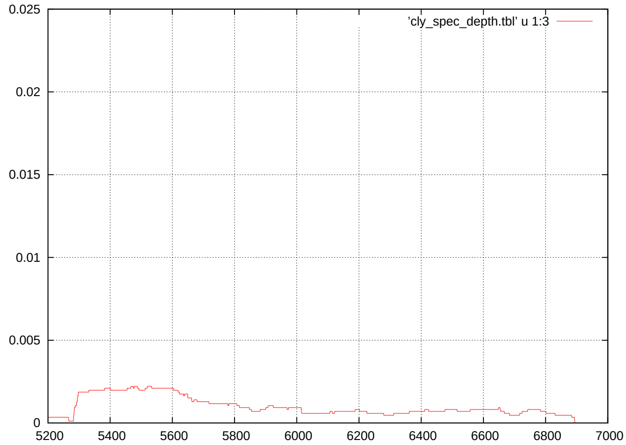
'cly_spec_depth.tbl' u 1:3
0.025
0.02
0.015
0.01
0.005
0
5200 5400 5600 5800 6000 6200 6400 6600 6800 7000

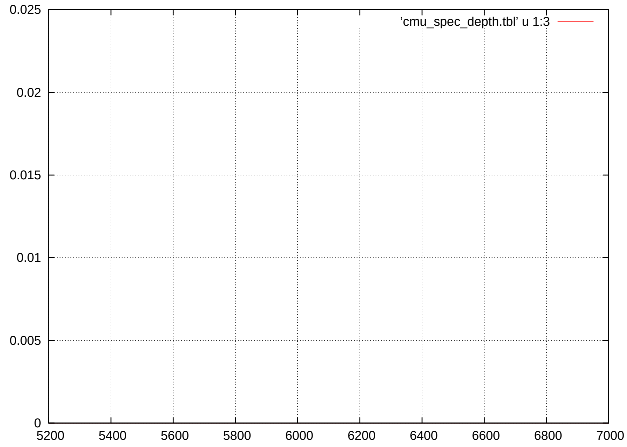
'cmu_spec_depth.tbl' u 1:3
0.025
0.02
0.015
0.01
0.005
0
5200 5400 5600 5800 6000 6200 6400 6600 6800 7000

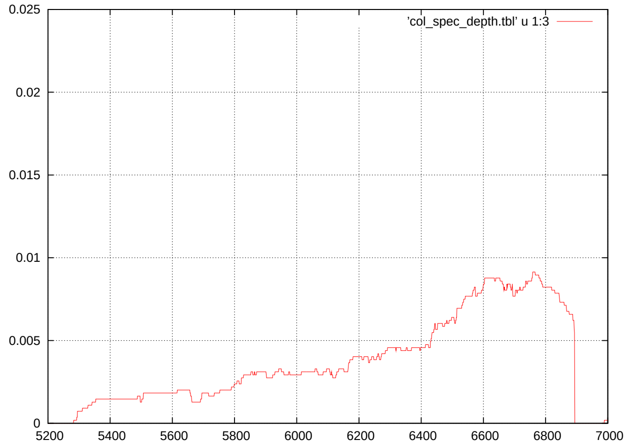
'col_spec_depth.tbl' u 1:3
0.025
0.02
0.015
0.01
0.005
0
5200 5400 5600 5800 6000 6200 6400 6600 6800 7000

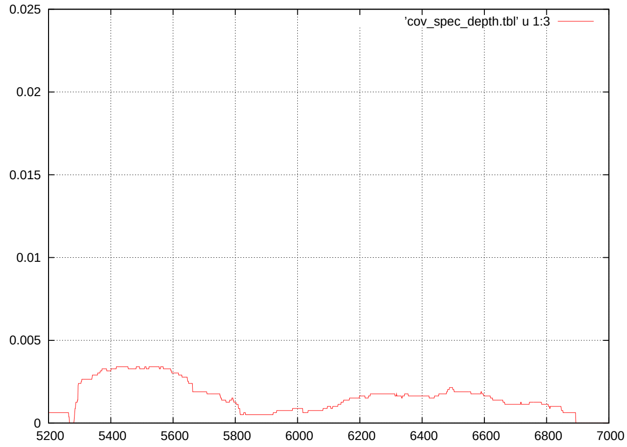
'cov_spec_depth.tbl' u 1:3
0.025
0.02
0.015
0.01
0.005
0
5200 5400 5600 5800 6000 6200 6400 6600 6800 7000

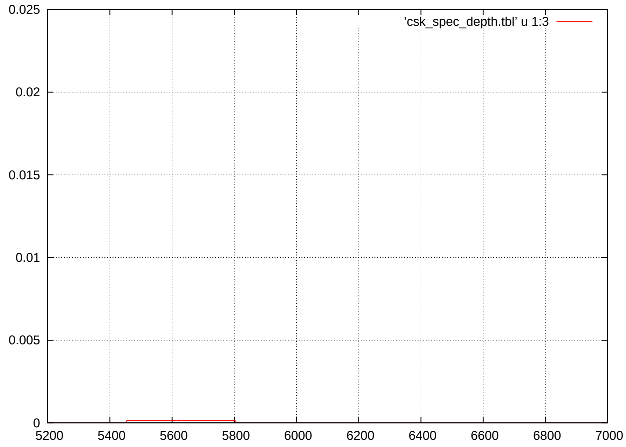
'csk_spec_depth.tbl' u 1:3
0.025
0.02
0.015
0.01
0.005
0
5200 5400 5600 5800 6000 6200 6400 6600 6800 7000

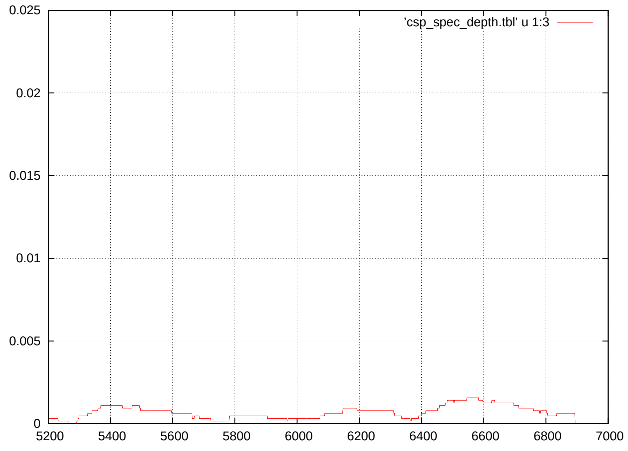
'csp_spec_depth.tbl' u 1:3
0.025
0.02
0.015
0.01
0.005
0
5200 5400 5600 5800 6000 6200 6400 6600 6800 7000

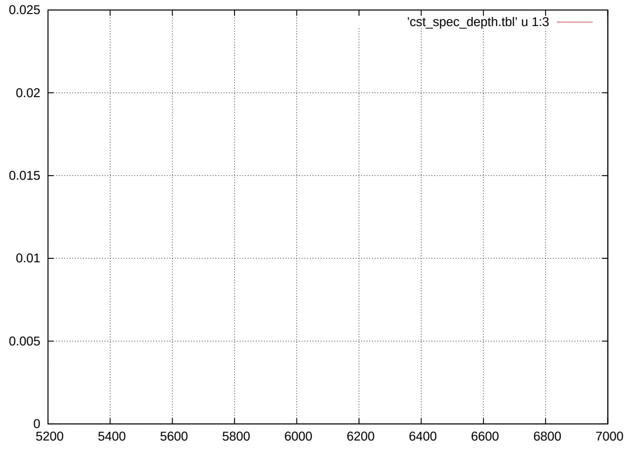
'cst_spec_depth.tbl' u 1:3
0.025
0.02
0.015
0.01
0.005
0
5200 5400 5600 5800 6000 6200 6400 6600 6800 7000

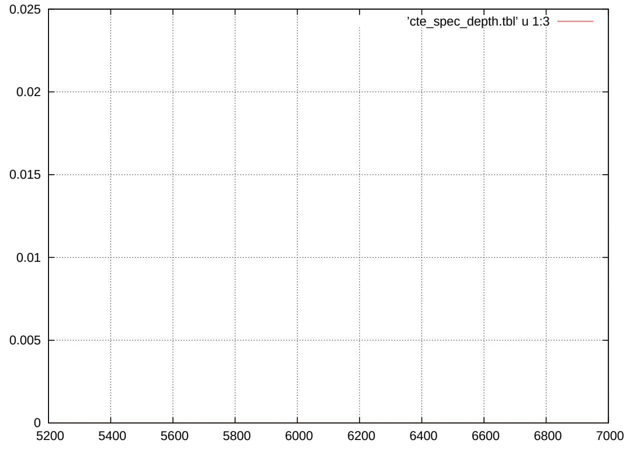
'cte_spec_depth.tbl' u 1:3
0.025
0.02
0.015
0.01
0.005
0
5200 5400 5600 5800 6000 6200 6400 6600 6800 7000

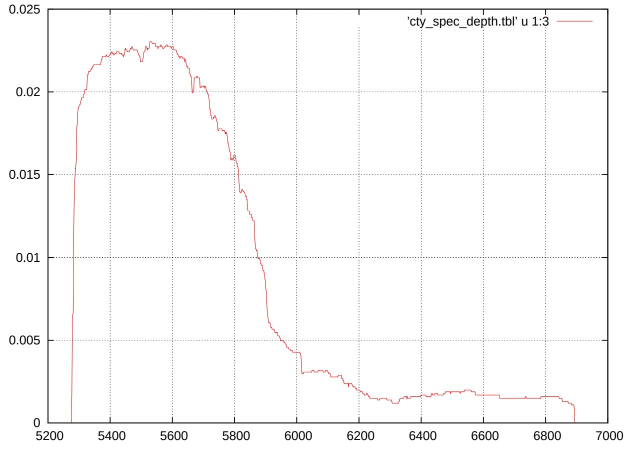
'cty_spec_depth.tbl' u 1:3
0.025
0.02
0.015
0.01
0.005
0
5200 5400 5600 5800 6000 6200 6400 6600 6800 7000

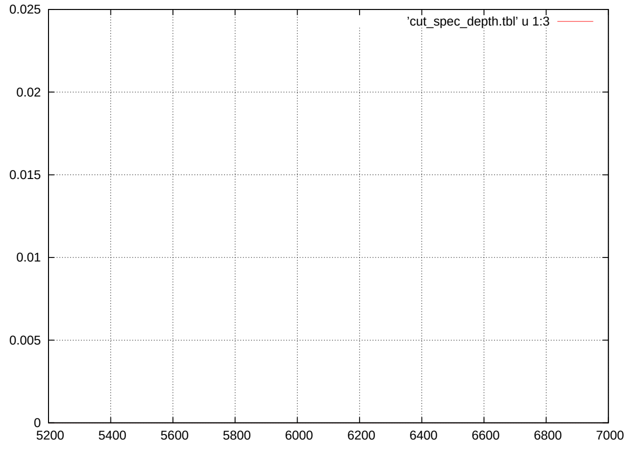
'cut_spec_depth.tbl' u 1:3
0.025
0.02
0.015
0.01
0.005
0
5200 5400 5600 5800 6000 6200 6400 6600 6800 7000

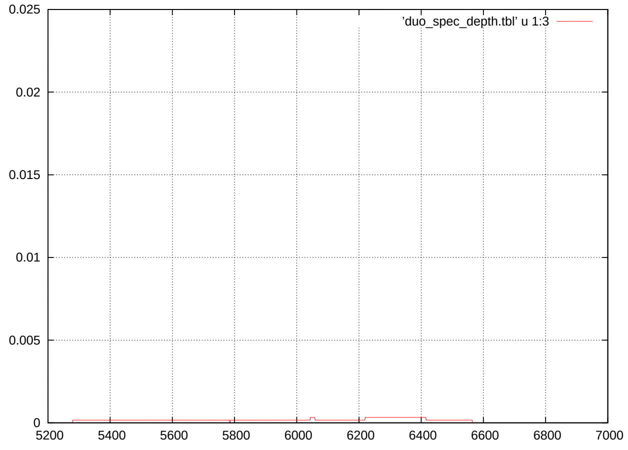
'duo_spec_depth.tbl' u 1:3
0.025
0.02
0.015
0.01
0.005
0
5200 5400 5600 5800 6000 6200 6400 6600 6800 7000

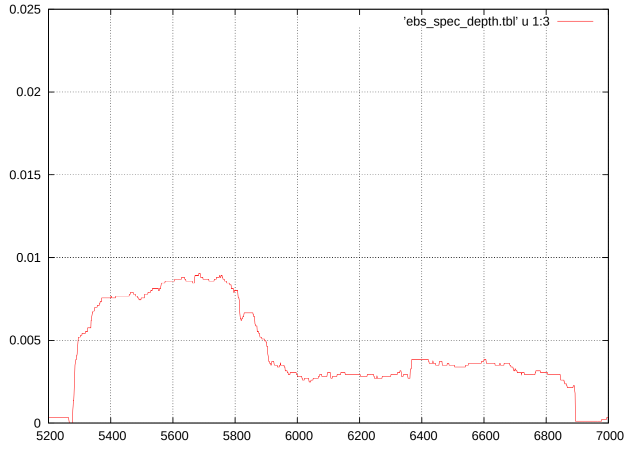
'ebs_spec_depth.tbl' u 1:3
0.025
0.02
0.015
0.01
0.005
0
5200 5400 5600 5800 6000 6200 6400 6600 6800 7000

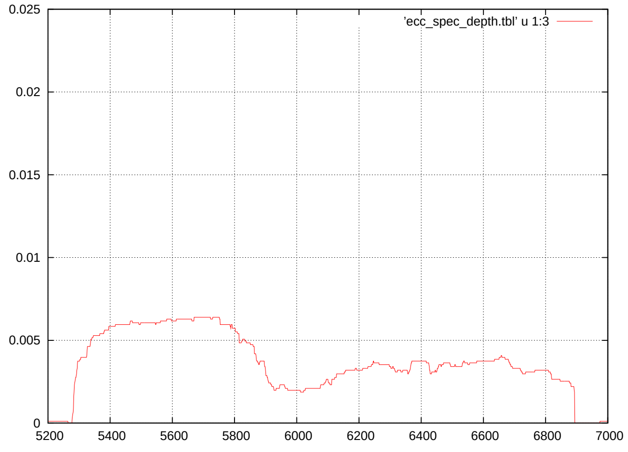
'ecc_spec_depth.tbl' u 1:3
0.025
0.02
0.015
0.01
0.005
0
5200 5400 5600 5800 6000 6200 6400 6600 6800 7000

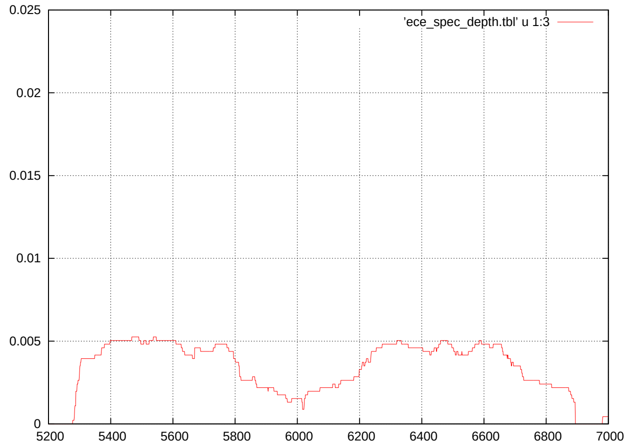
'ece_spec_depth.tbl' u 1:3
0.025
0.02
0.015
0.01
0.005
0
5200 5400 5600 5800 6000 6200 6400 6600 6800 7000

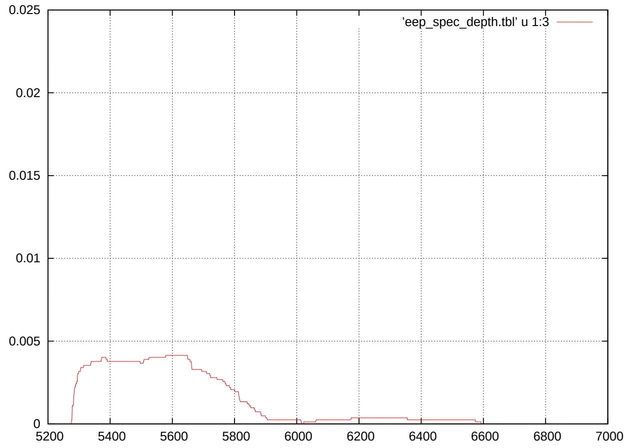
'eep_spec_depth.tbl' u 1:3
0.025
0.02
0.015
0.01
0.005
0
5200 5400 5600 5800 6000 6200 6400 6600 6800 7000

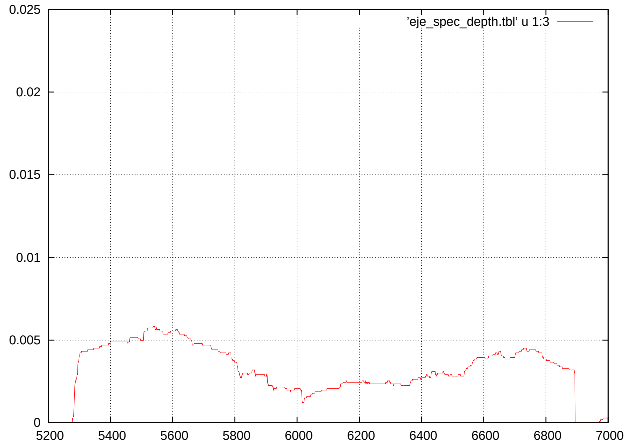
'eje_spec_depth.tbl' u 1:3
0.025
0.02
0.015
0.01
0.005
0
5200 5400 5600 5800 6000 6200 6400 6600 6800 7000

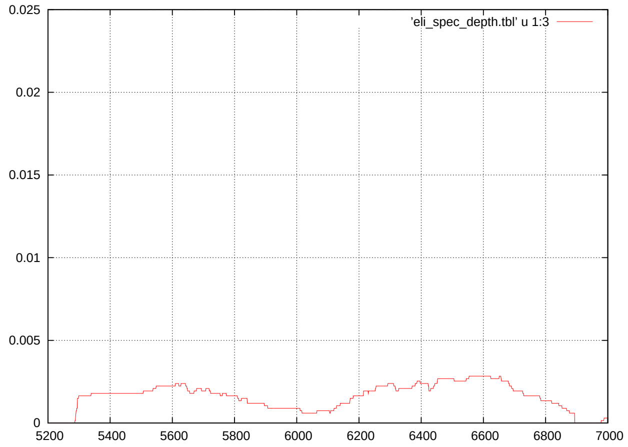
'eli_spec_depth.tbl' u 1:3
0.025
0.02
0.015
0.01
0.005
0
5200 5400 5600 5800 6000 6200 6400 6600 6800 7000

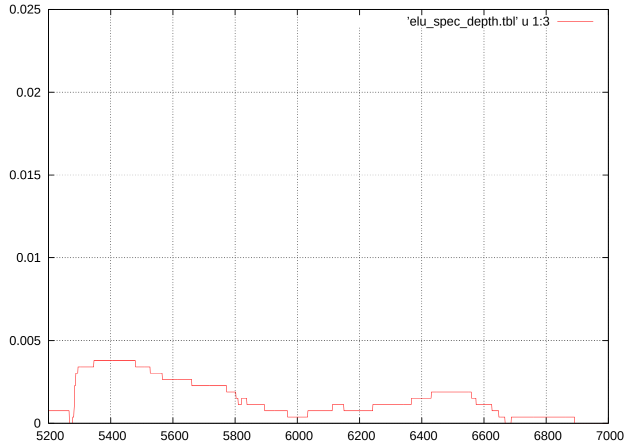
'elu_spec_depth.tbl' u 1:3
0.025
0.02
0.015
0.01
0.005
0
5200 5400 5600 5800 6000 6200 6400 6600 6800 7000

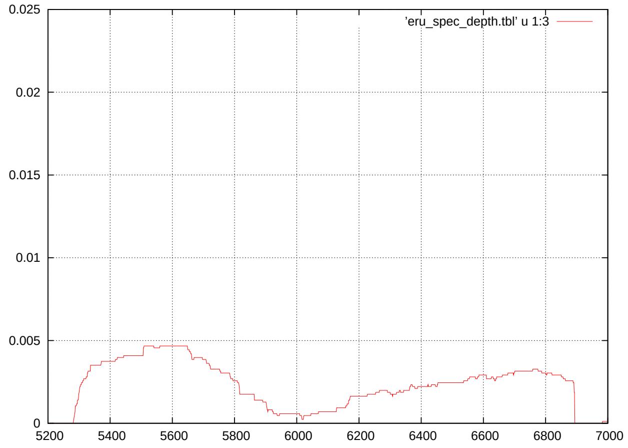
'eru_spec_depth.tbl' u 1:3
0.025
0.02
0.015
0.01
0.005
0
5200 5400 5600 5800 6000 6200 6400 6600 6800 7000

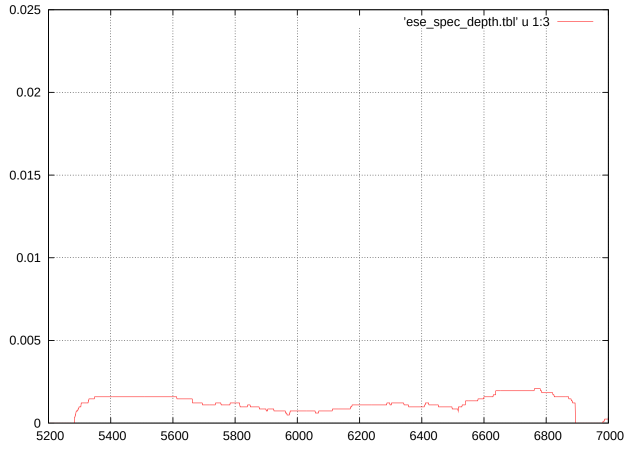
'ese_spec_depth.tbl' u 1:3
0.025
0.02
0.015
0.01
0.005
0
5200 5400 5600 5800 6000 6200 6400 6600 6800 7000

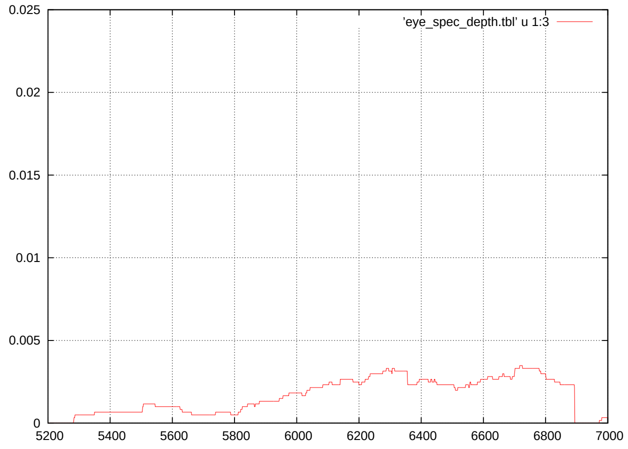
'eye_spec_depth.tbl' u 1:3
0.025
0.02
0.015
0.01
0.005
0
5200 5400 5600 5800 6000 6200 6400 6600 6800 7000

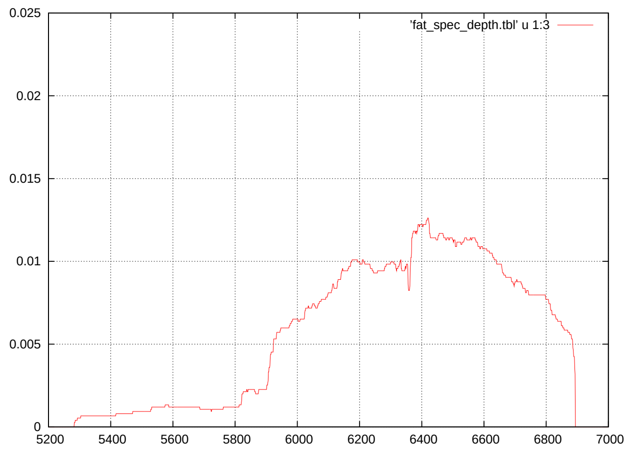
'fat_spec_depth.tbl' u 1:3
0.025
0.02
0.015
0.01
0.005
0
5200 5400 5600 5800 6000 6200 6400 6600 6800 7000

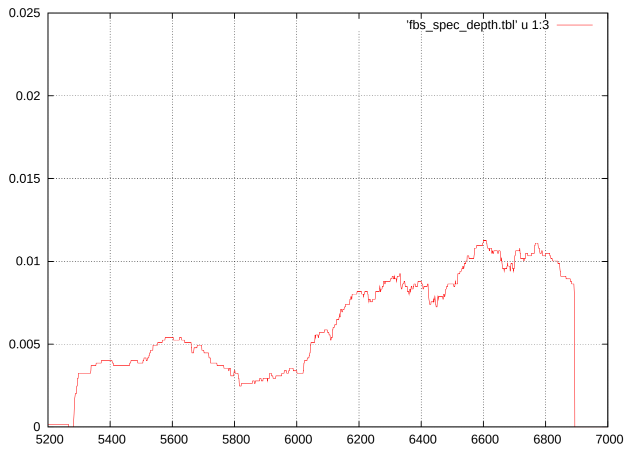
'fbs_spec_depth.tbl' u 1:3
0.025
0.02
0.015
0.01
0.005
0
5200 5400 5600 5800 6000 6200 6400 6600 6800 7000

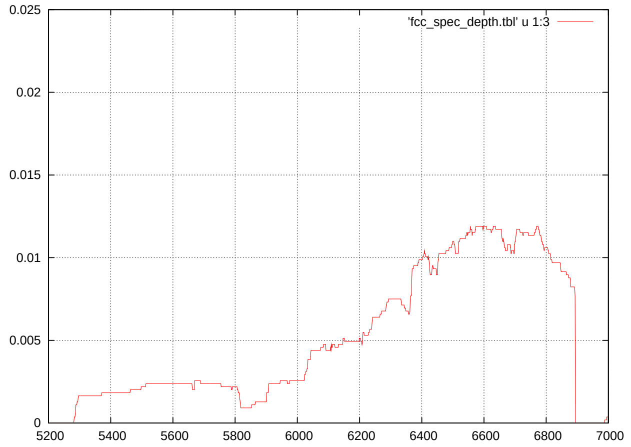
'fcc_spec_depth.tbl' u 1:3
0.025
0.02
0.015
0.01
0.005
0
5200 5400 5600 5800 6000 6200 6400 6600 6800 7000

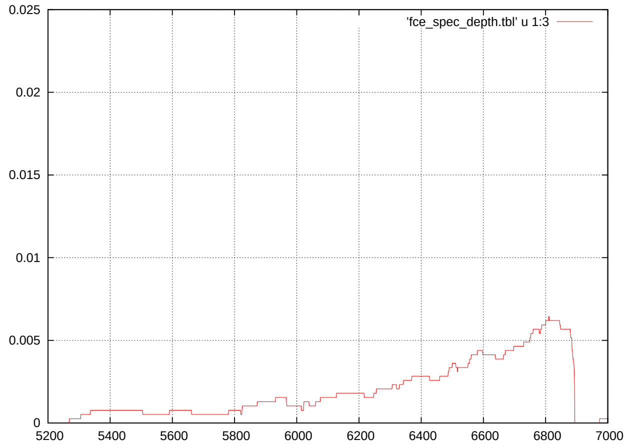
'fce_spec_depth.tbl' u 1:3
0.025
0.02
0.015
0.01
0.005
0
5200 5400 5600 5800 6000 6200 6400 6600 6800 7000

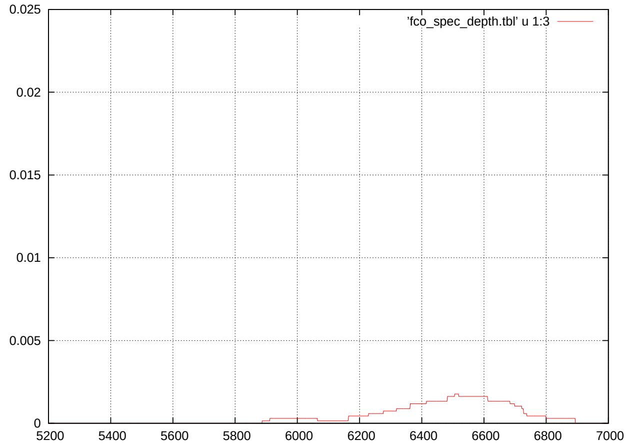
'fco_spec_depth.tbl' u 1:3
0.025
0.02
0.015
0.01
0.005
0
5200 5400 5600 5800 6000 6200 6400 6600 6800 7000

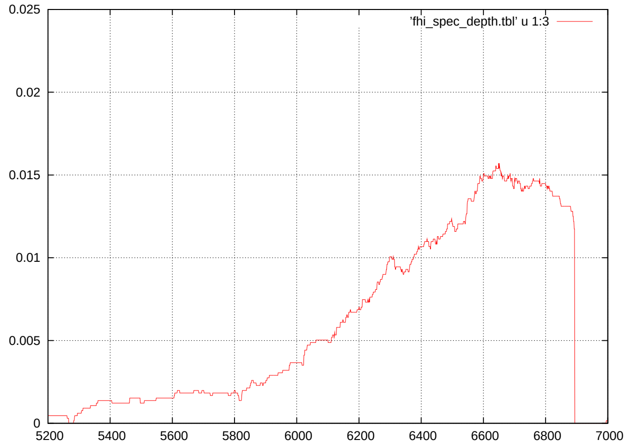
'fhi_spec_depth.tbl' u 1:3
0.025
0.02
0.015
0.01
0.005
0
5200 5400 5600 5800 6000 6200 6400 6600 6800 7000

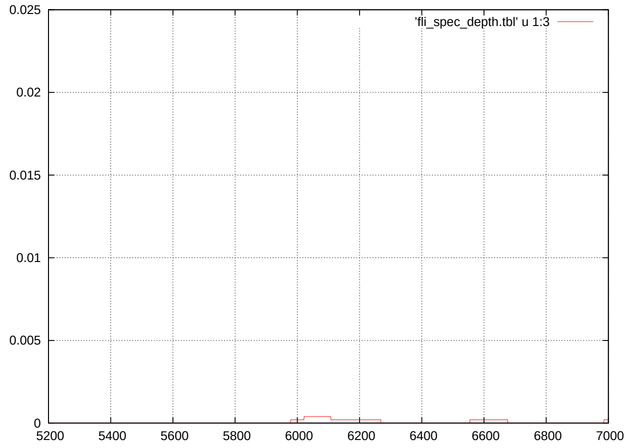
'fli_spec_depth.tbl' u 1:3
0.025
0.02
0.015
0.01
0.005
0
5200 5400 5600 5800 6000 6200 6400 6600 6800 7000

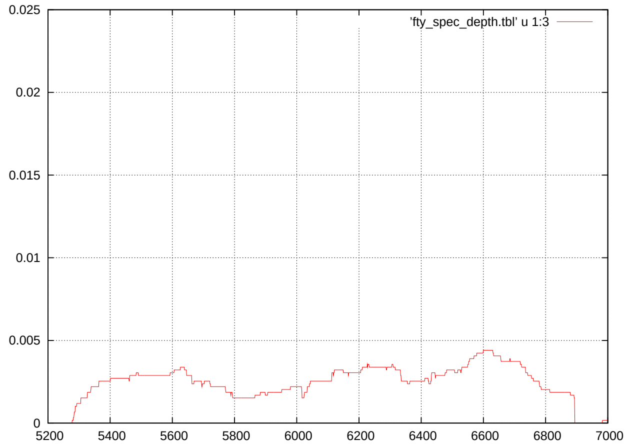
'fty_spec_depth.tbl' u 1:3
0.025
0.02
0.015
0.01
0.005
0
5200 5400 5600 5800 6000 6200 6400 6600 6800 7000

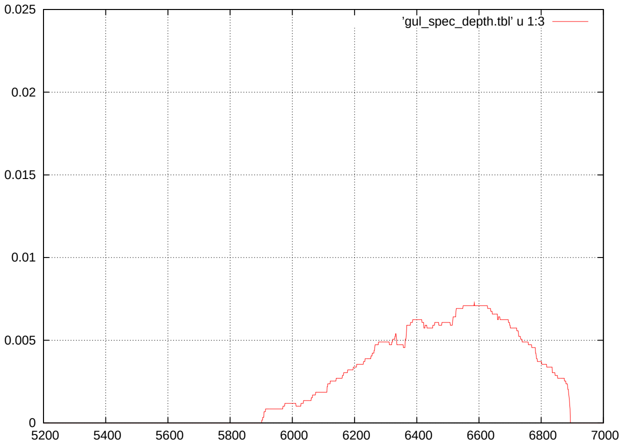
'gul_spec_depth.tbl' u 1:3
0 0.005 0.01 0.015 0.02 0.025
5200 5400 5600 5800 6000 6200 6400 6600 6800 7000

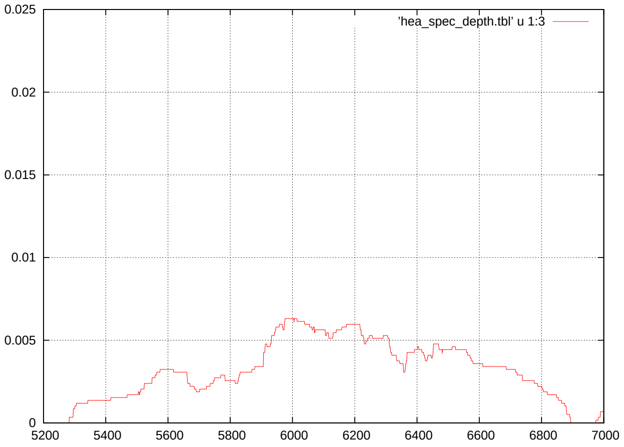
'hea_spec_depth.tbl' u 1:3
0 0.005 0.01 0.015 0.02 0.025
5200 5400 5600 5800 6000 6200 6400 6600 6800 7000

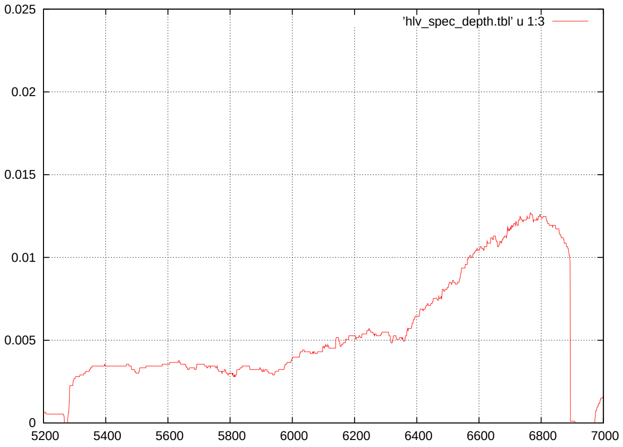
'hlv_spec_depth.tbl' u 1:3
0 0.005 0.01 0.015 0.02 0.025
5200 5400 5600 5800 6000 6200 6400 6600 6800 7000

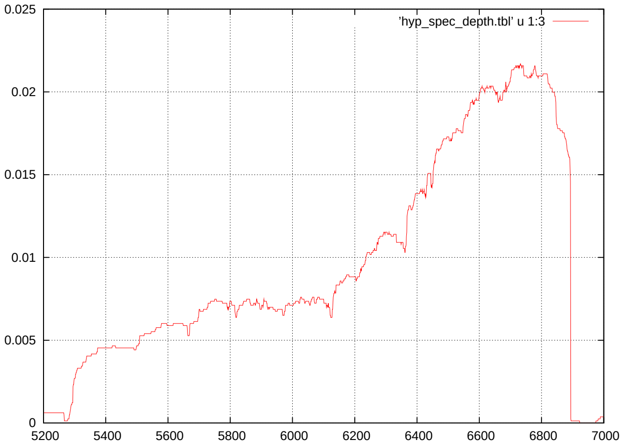
'hyp_spec_depth.tbl' u 1:3
0 0.005 0.01 0.015 0.02 0.025
5200 5400 5600 5800 6000 6200 6400 6600 6800 7000

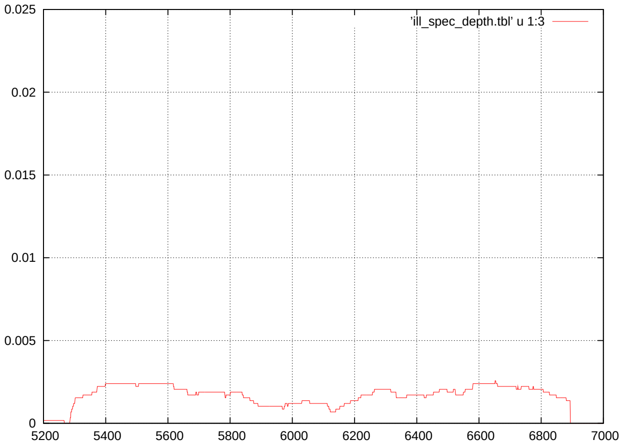
'ill_spec_depth.tbl' u 1:3
0 0.005 0.01 0.015 0.02 0.025
5200 5400 5600 5800 6000 6200 6400 6600 6800 7000

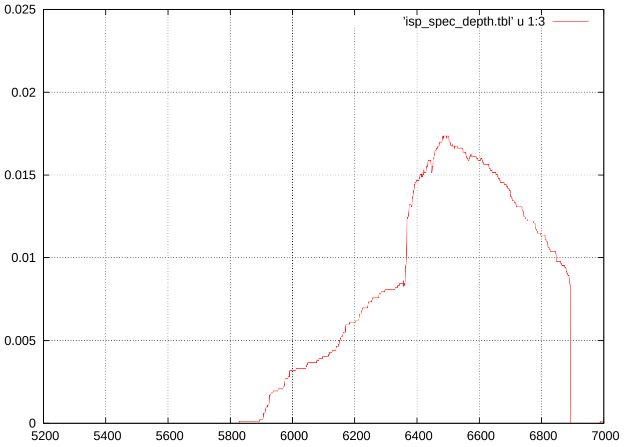
'isp_spec_depth.tbl' u 1:3
0 0.005 0.01 0.015 0.02 0.025
5200 5400 5600 5800 6000 6200 6400 6600 6800 7000

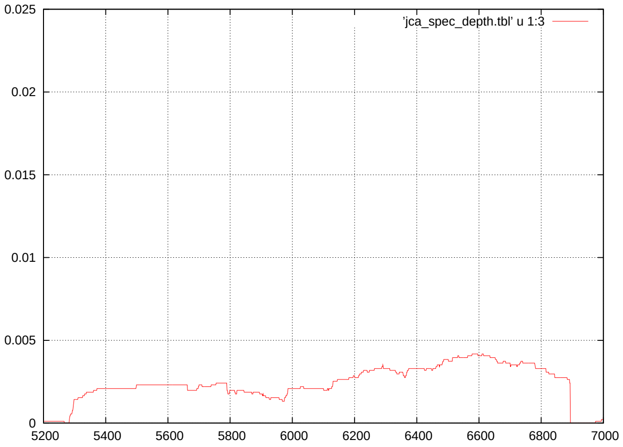
'jca_spec_depth.tbl' u 1:3
0 0.005 0.01 0.015 0.02 0.025
5200 5400 5600 5800 6000 6200 6400 6600 6800 7000

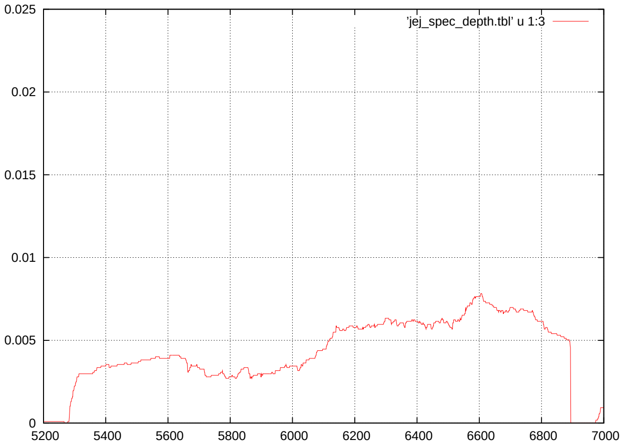
'jej_spec_depth.tbl' u 1:3
0 0.005 0.01 0.015 0.02 0.025
5200 5400 5600 5800 6000 6200 6400 6600 6800 7000

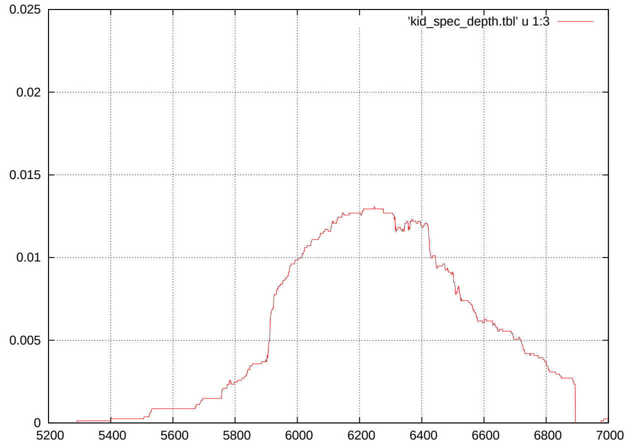
'kid_spec_depth.tbl' u 1:3
0.025
0.02
0.015
0.01
0.005
0
5200 5400 5600 5800 6000 6200 6400 6600 6800 7000

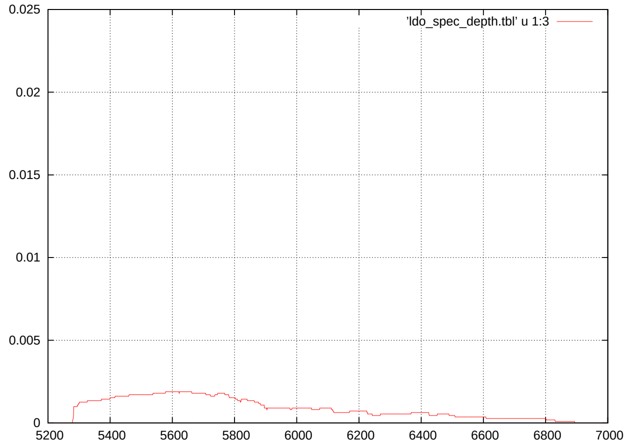
'ldo_spec_depth.tbl' u 1:3
0.025
0.02
0.015
0.01
0.005
0
5200 5400 5600 5800 6000 6200 6400 6600 6800 7000

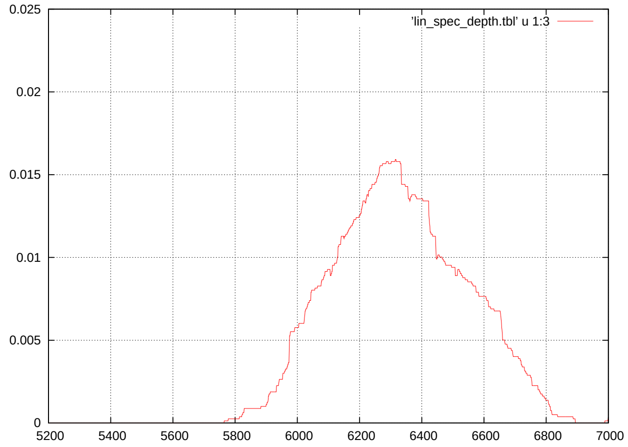
'lin_spec_depth.tbl' u 1:3
0.025
0.02
0.015
0.01
0.005
0
5200 5400 5600 5800 6000 6200 6400 6600 6800 7000

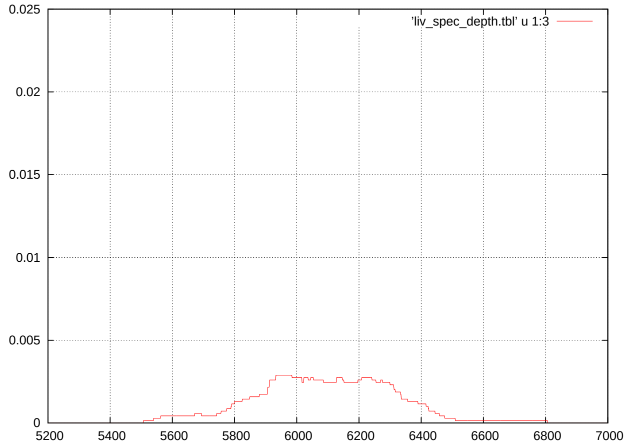
'liv_spec_depth.tbl' u 1:3
0.025
0.02
0.015
0.01
0.005
0
5200 5400 5600 5800 6000 6200 6400 6600 6800 7000

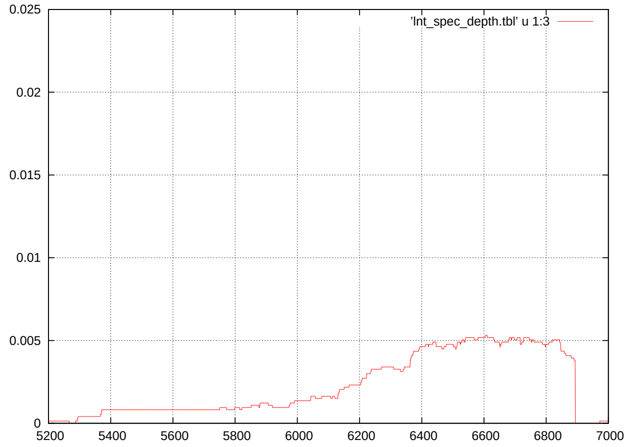
'lnt_spec_depth.tbl' u 1:3
0.025
0.02
0.015
0.01
0.005
0
5200 5400 5600 5800 6000 6200 6400 6600 6800 7000

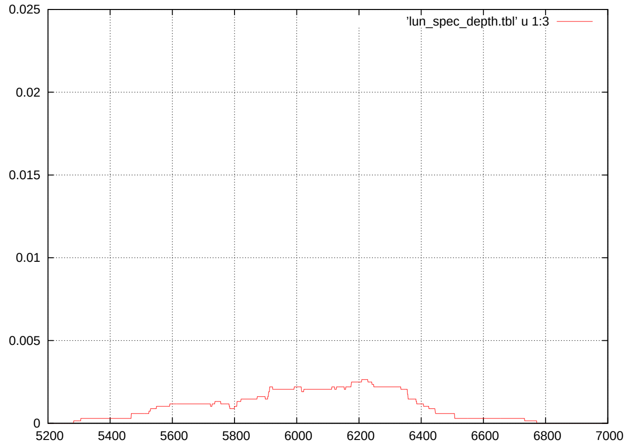
'lun_spec_depth.tbl' u 1:3
0.025
0.02
0.015
0.01
0.005
0
5200 5400 5600 5800 6000 6200 6400 6600 6800 7000

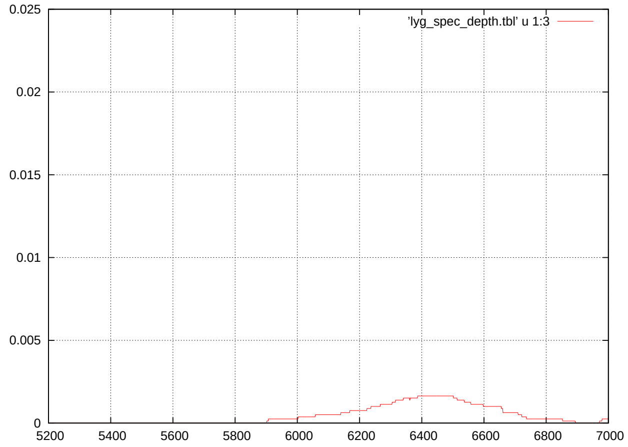
'lyg_spec_depth.tbl' u 1:3
0.025
0.02
0.015
0.01
0.005
0
5200 5400 5600 5800 6000 6200 6400 6600 6800 7000

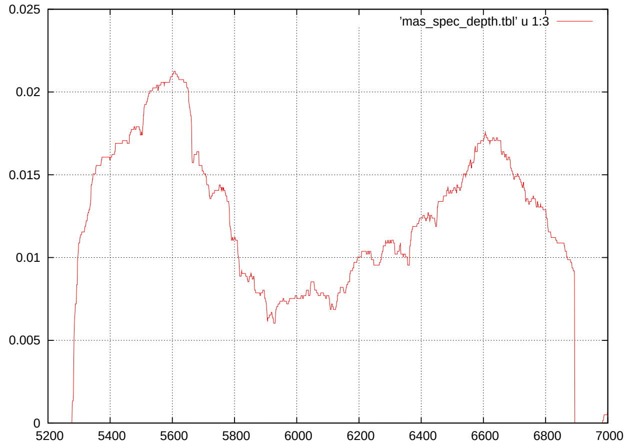
'mas_spec_depth.tbl' u 1:3
0.025
0.02
0.015
0.01
0.005
0
5200 5400 5600 5800 6000 6200 6400 6600 6800 7000

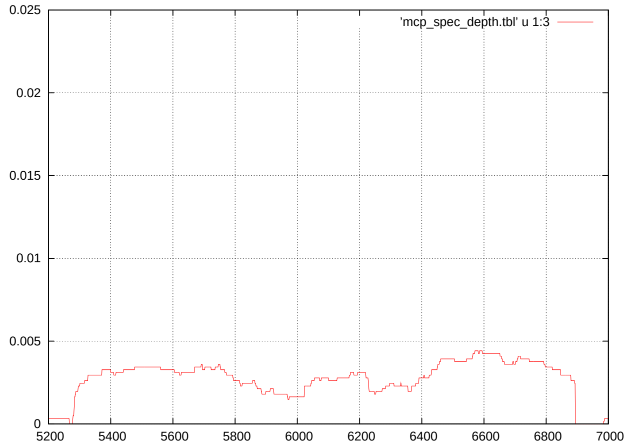
'mcp_spec_depth.tbl' u 1:3
0.025
0.02
0.015
0.01
0.005
0
5200 5400 5600 5800 6000 6200 6400 6600 6800 7000

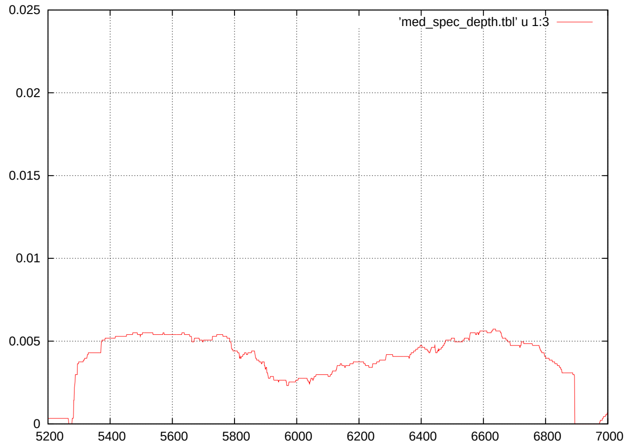
'med_spec_depth.tbl' u 1:3
0.025
0.02
0.015
0.01
0.005
0
5200 5400 5600 5800 6000 6200 6400 6600 6800 7000

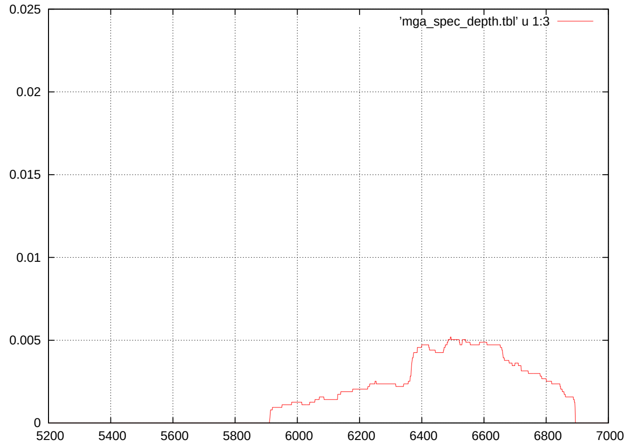
'mga_spec_depth.tbl' u 1:3
0.025
0.02
0.015
0.01
0.005
0
5200 5400 5600 5800 6000 6200 6400 6600 6800 7000

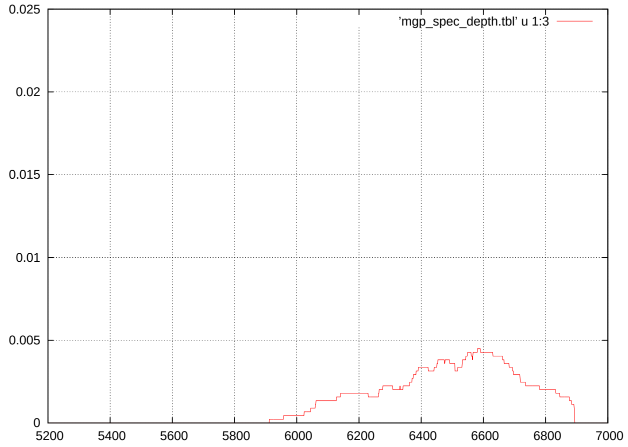
'mgp_spec_depth.tbl' u 1:3
0.025
0.02
0.015
0.01
0.005
0
5200 5400 5600 5800 6000 6200 6400 6600 6800 7000

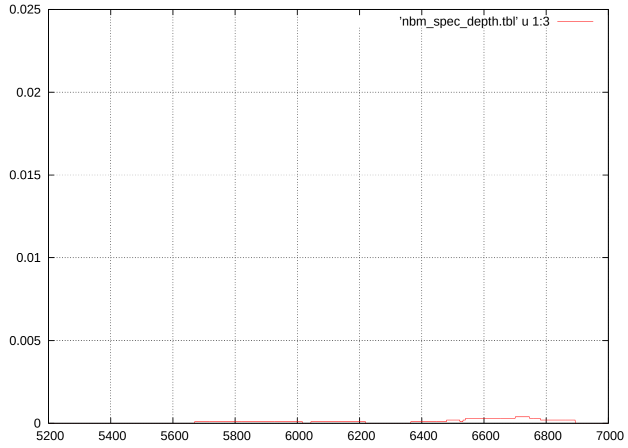
'nbm_spec_depth.tbl' u 1:3
0.025
0.02
0.015
0.01
0.005
0
5200 5400 5600 5800 6000 6200 6400 6600 6800 7000

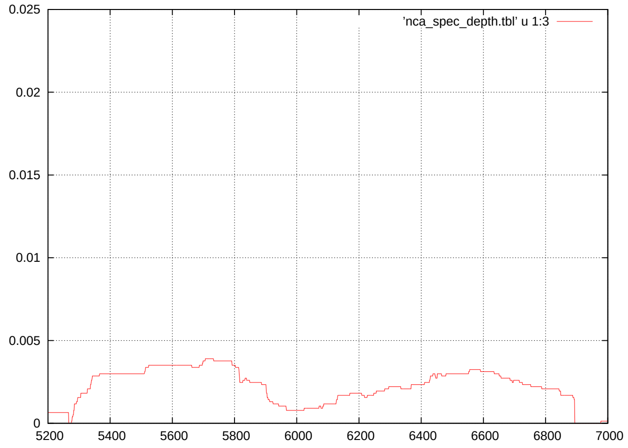
'nca_spec_depth.tbl' u 1:3
0.025
0.02
0.015
0.01
0.005
0
5200 5400 5600 5800 6000 6200 6400 6600 6800 7000

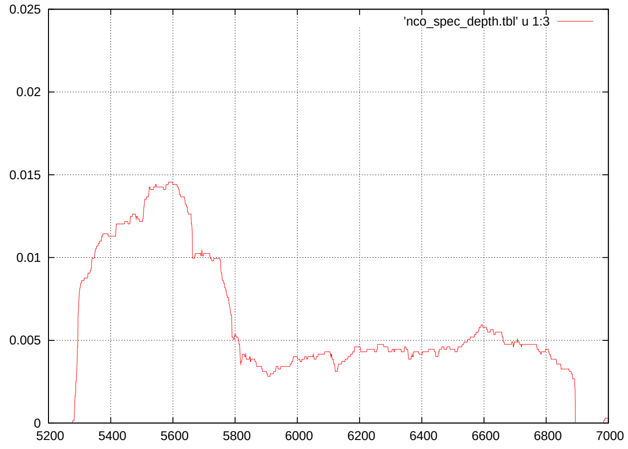
'nco_spec_depth.tbl' u 1:3
0.025
0.02
0.015
0.01
0.005
0
5200 5400 5600 5800 6000 6200 6400 6600 6800 7000

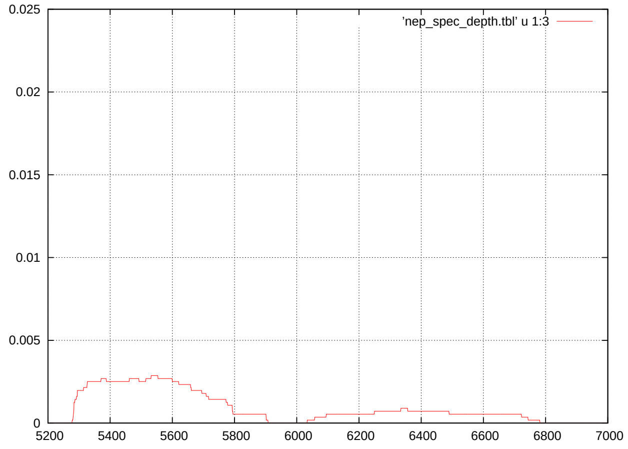
'nep_spec_depth.tbl' u 1:3
0.025
0.02
0.015
0.01
0.005
0
5200 5400 5600 5800 6000 6200 6400 6600 6800 7000

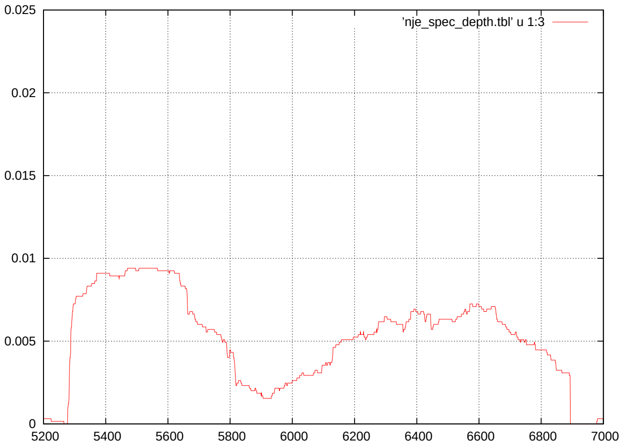
'nje_spec_depth.tbl' u 1:3
0.025
0.02
0.015
0.01
0.005
0
5200 5400 5600 5800 6000 6200 6400 6600 6800 7000

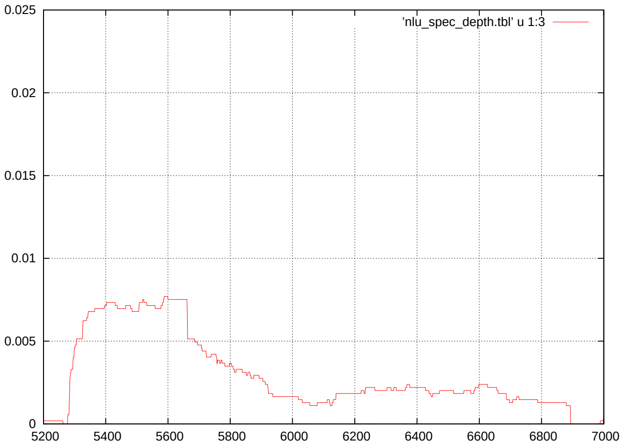
'nlu_spec_depth.tbl' u 1:3
0.025
0.02
0.015
0.01
0.005
0
5200 5400 5600 5800 6000 6200 6400 6600 6800 7000

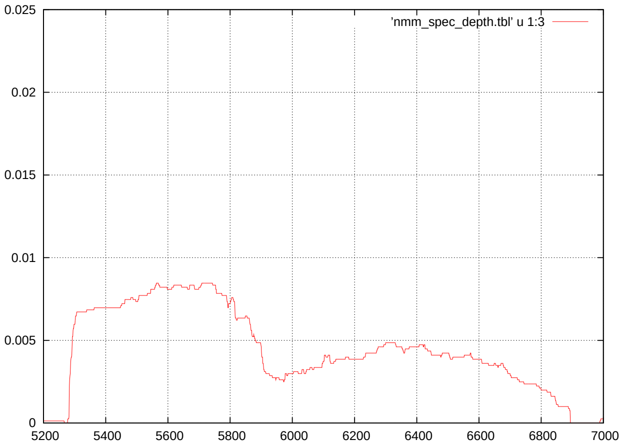
'nmm_spec_depth.tbl' u 1:3
0.025
0.02
0.015
0.01
0.005
0
5200 5400 5600 5800 6000 6200 6400 6600 6800 7000

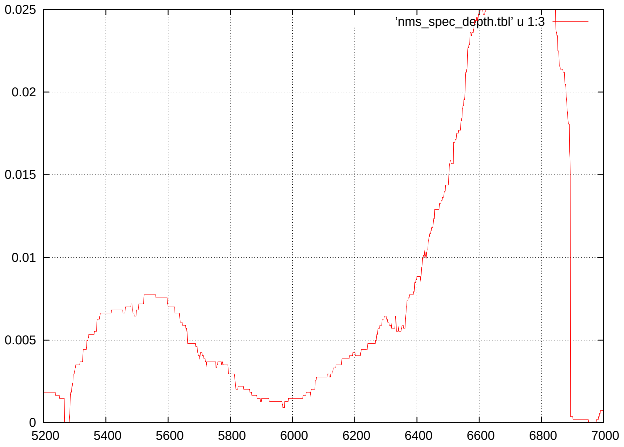
'nms_spec_depth.tbl' u 1:3
0.025
0.02
0.015
0.01
0.005
0
5200 5400 5600 5800 6000 6200 6400 6600 6800 7000

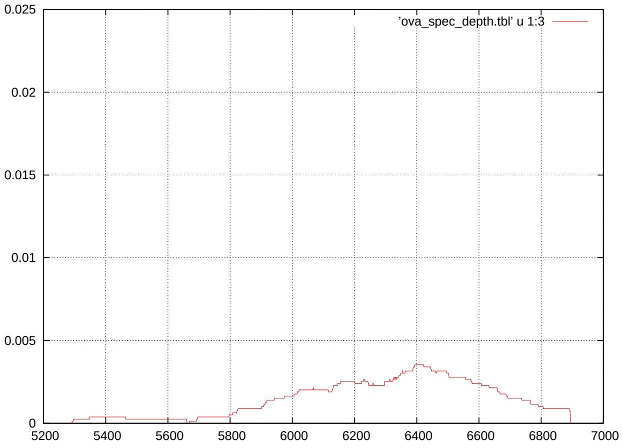
'ova_spec_depth.tbl' u 1:3
0.025
0.02
0.015
0.01
0.005
0
5200 5400 5600 5800 6000 6200 6400 6600 6800 7000

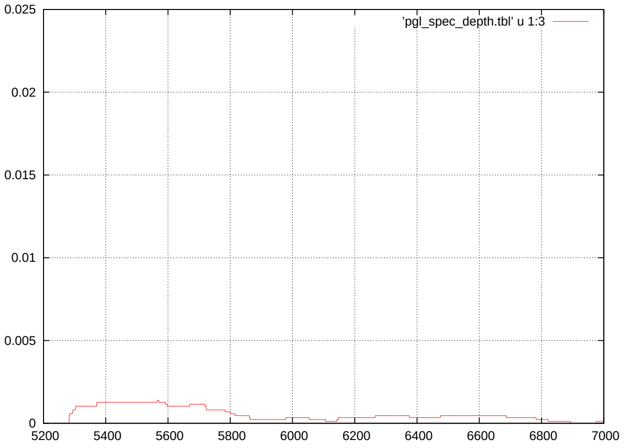
'pgl_spec_depth.tbl' u 1:3
0.025
0.02
0.015
0.01
0.005
0
5200 5400 5600 5800 6000 6200 6400 6600 6800 7000

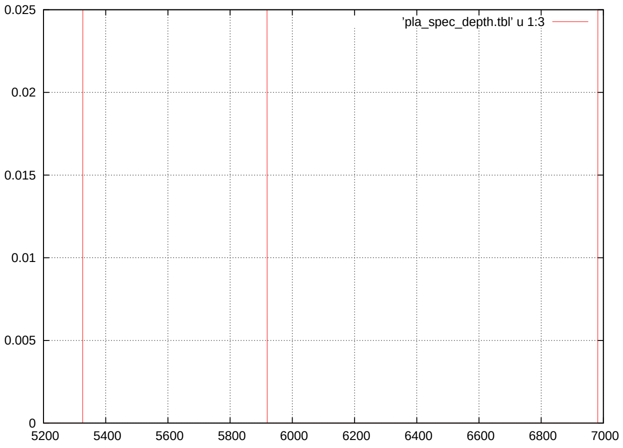
'pla_spec_depth.tbl' u 1:3
0.025
0.02
0.015
0.01
0.005
0
5200 5400 5600 5800 6000 6200 6400 6600 6800 7000

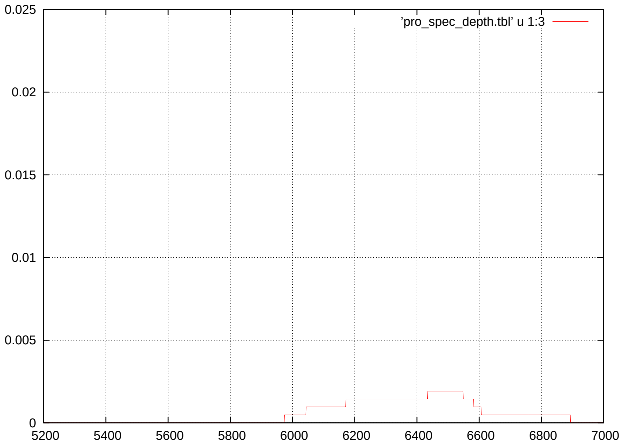
'pro_spec_depth.tbl' u 1:3
0.025
0.02
0.015
0.01
0.005
0
5200 5400 5600 5800 6000 6200 6400 6600 6800 7000

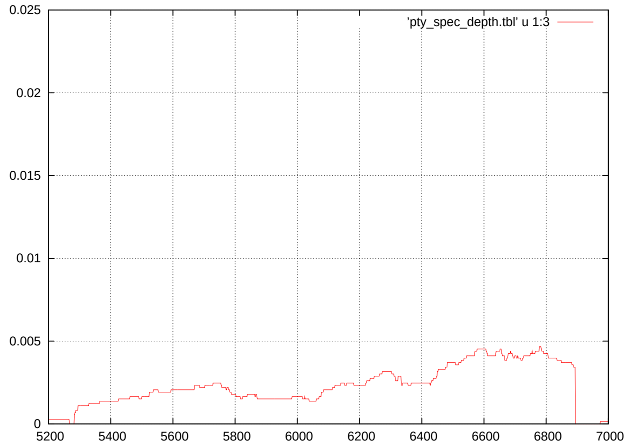
'pty_spec_depth.tbl' u 1:3
0.025
0.02
0.015
0.01
0.005
0
5200
5400
5600
5800
6000
6200
6400
6600
6800
7000

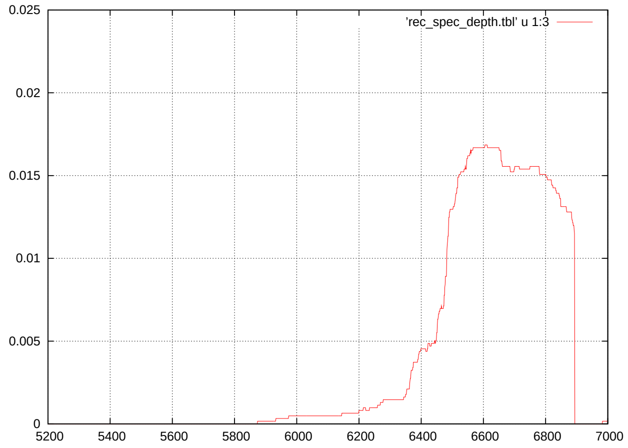
'rec_spec_depth.tbl' u 1:3
0.025
0.02
0.015
0.01
0.005
0
5200
5400
5600
5800
6000
6200
6400
6600
6800
7000

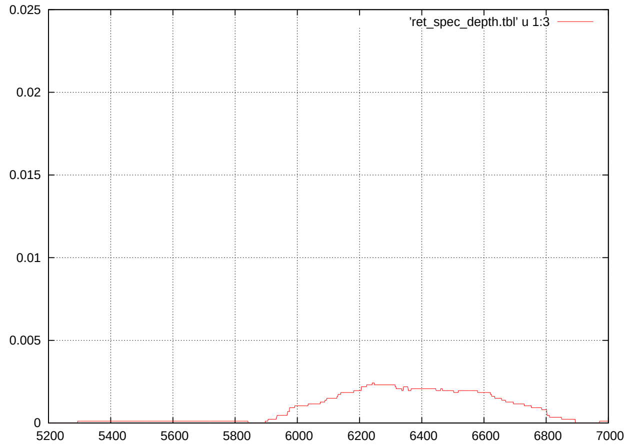
'ret_spec_depth.tbl' u 1:3
0.025
0.02
0.015
0.01
0.005
0
5200
5400
5600
5800
6000
6200
6400
6600
6800
7000

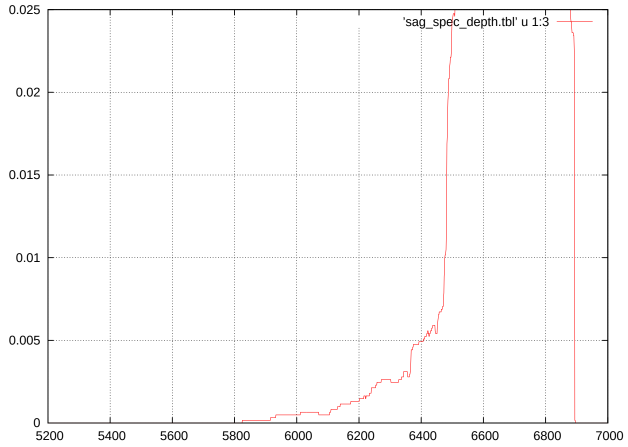
'sag_spec_depth.tbl' u 1:3
0.025
0.02
0.015
0.01
0.005
0
5200
5400
5600
5800
6000
6200
6400
6600
6800
7000

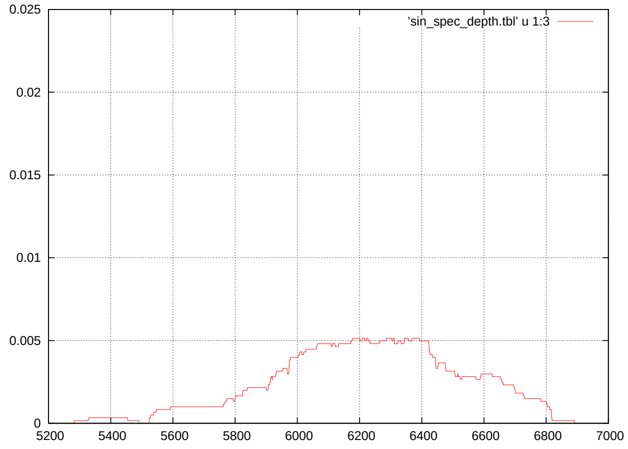
'sin_spec_depth.tbl' u 1:3
0.025
0.02
0.015
0.01
0.005
0
5200
5400
5600
5800
6000
6200
6400
6600
6800
7000

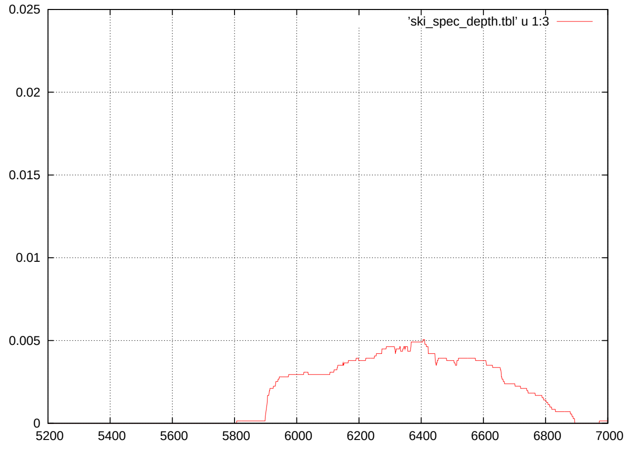
'ski_spec_depth.tbl' u 1:3
0.025
0.02
0.015
0.01
0.005
0
5200
5400
5600
5800
6000
6200
6400
6600
6800
7000

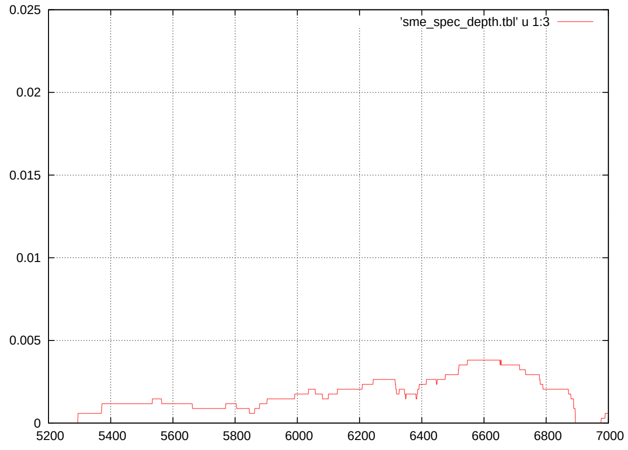
'sme_spec_depth.tbl' u 1:3
0.025
0.02
0.015
0.01
0.005
0
5200
5400
5600
5800
6000
6200
6400
6600
6800
7000

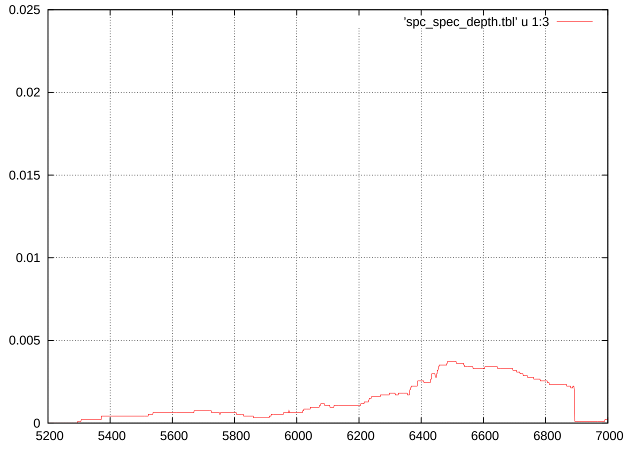
'spc_spec_depth.tbl' u 1:3
0.025
0.02
0.015
0.01
0.005
0
5200
5400
5600
5800
6000
6200
6400
6600
6800
7000

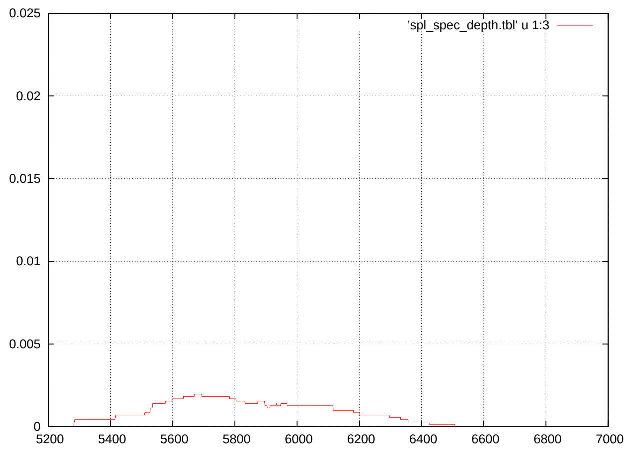
'spl_spec_depth.tbl' u 1:3
0.025
0.02
0.015
0.01
0.005
0
5200 5400 5600 5800 6000 6200 6400 6600 6800 7000

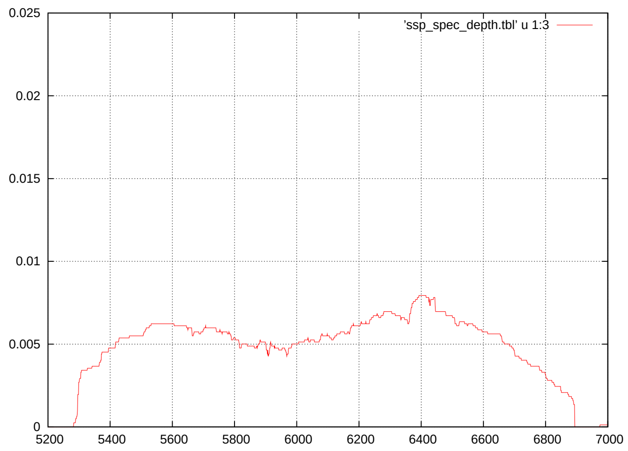
'ssp_spec_depth.tbl' u 1:3
0.025
0.02
0.015
0.01
0.005
0
5200 5400 5600 5800 6000 6200 6400 6600 6800 7000

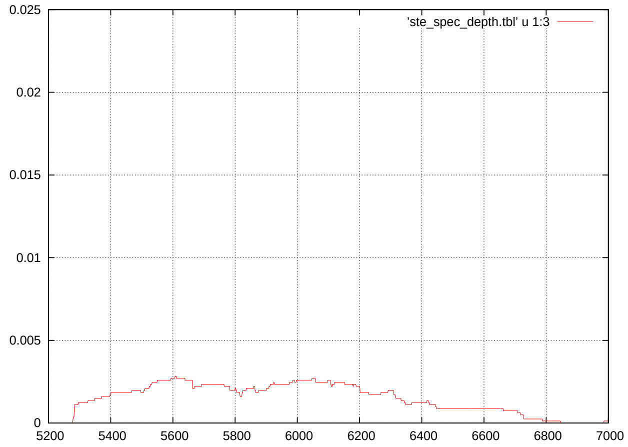
'ste_spec_depth.tbl' u 1:3
0.025
0.02
0.015
0.01
0.005
0
5200 5400 5600 5800 6000 6200 6400 6600 6800 7000

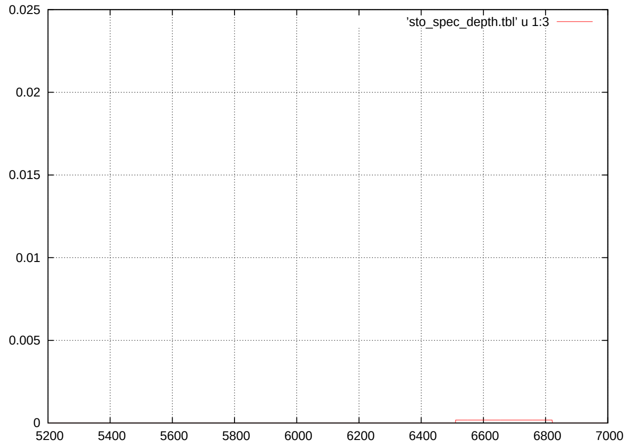
'sto_spec_depth.tbl' u 1:3
0.025
0.02
0.015
0.01
0.005
0
5200 5400 5600 5800 6000 6200 6400 6600 6800 7000

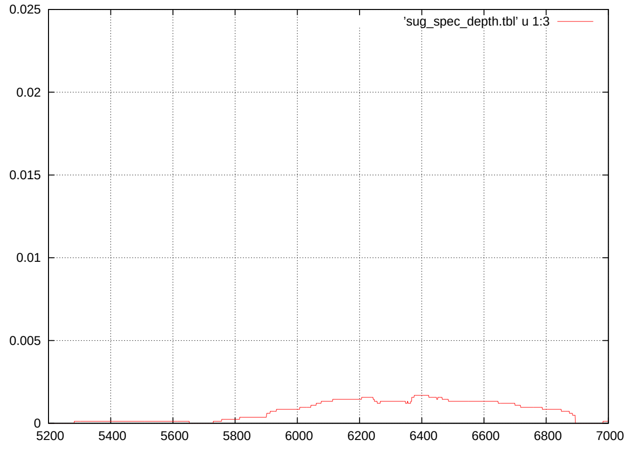
'sug_spec_depth.tbl' u 1:3
0.025
0.02
0.015
0.01
0.005
0
5200 5400 5600 5800 6000 6200 6400 6600 6800 7000

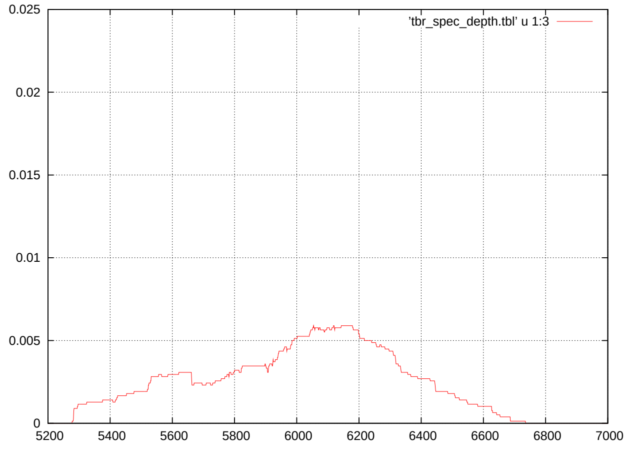
'tbr_spec_depth.tbl' u 1:3
0.025
0.02
0.015
0.01
0.005
0
5200 5400 5600 5800 6000 6200 6400 6600 6800 7000

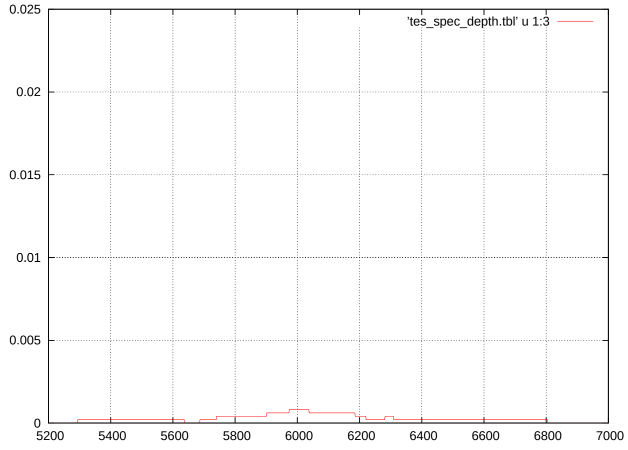
'tes_spec_depth.tbl' u 1:3
0.025
0.02
0.015
0.01
0.005
0
5200 5400 5600 5800 6000 6200 6400 6600 6800 7000

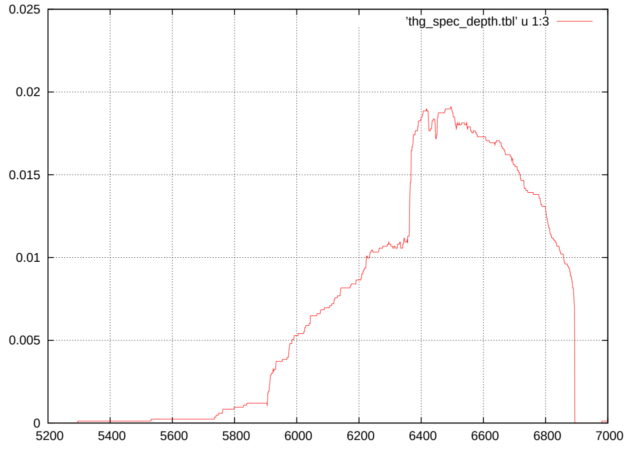
'thg_spec_depth.tbl' u 1:3
0.025
0.02
0.015
0.01
0.005
0
5200 5400 5600 5800 6000 6200 6400 6600 6800 7000

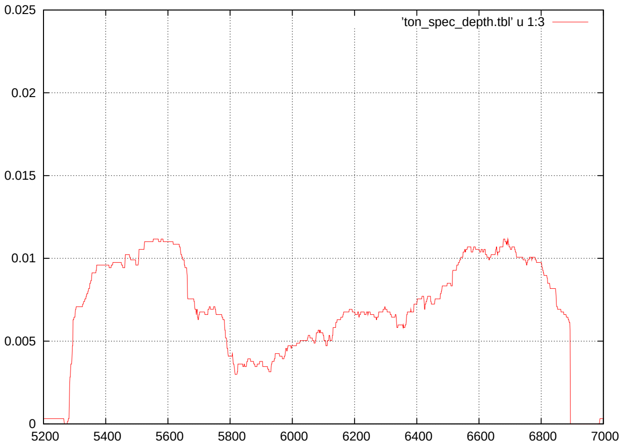

'ton_spec_depth.tbl' u 1:3
0.025
0.02
0.015
0.01
0.005
0
5200 5400 5600 5800 6000 6200 6400 6600 6800 7000

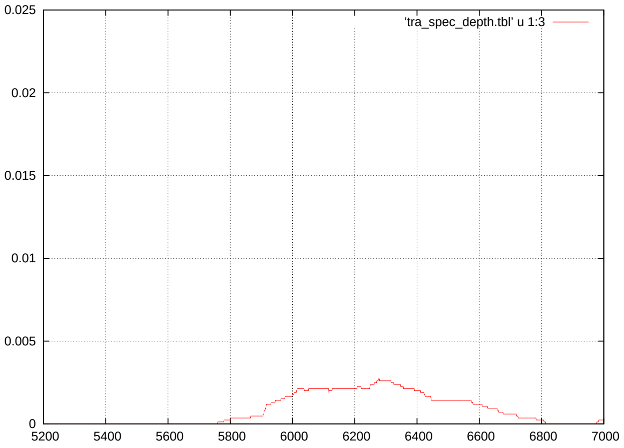

'tra_spec_depth.tbl' u 1:3
0.025
0.02
0.015
0.01
0.005
0
5200 5400 5600 5800 6000 6200 6400 6600 6800 7000

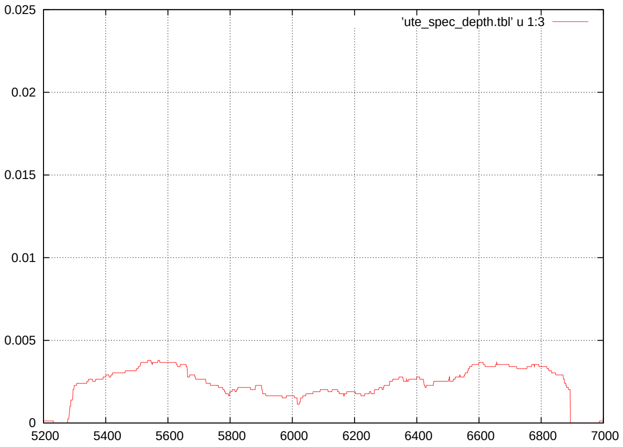

'ute_spec_depth.tbl' u 1:3
0.025
0.02
0.015
0.01
0.005
0
5200 5400 5600 5800 6000 6200 6400 6600 6800 7000

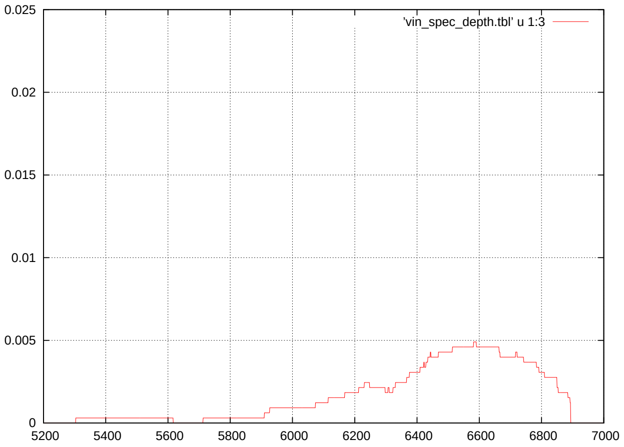

'vin_spec_depth.tbl' u 1:3
0.025
0.02
0.015
0.01
0.005
0
5200 5400 5600 5800 6000 6200 6400 6600 6800 7000
