# Supplementary material for: EST analysis on pig mitochondria reveal novel expression differences between developmental and adult tissues
Source: BMC Genomics. 2007 Oct 11;8:367. doi: 10.1186/1471-2164-8-367 (PMC2194790; doi:10.1186/1471-2164-8-367)
Supplement: Additional file 2 — COXI expression and clustering. Supplementary figures for COX I expression and cluster analysis. [file 1471-2164-8-367-S2.pdf]

Additional Data File 2: COXI expression and clustering

EST analysis on pig mitochondria reveal novel expression differences between developmental and adult tissues

Karsten Scheibye-Knudsen[1], Susanna Cirera[1], Michael J. Gilchrist[2], Merete Fredholm[1],
Jan Gorodkin*[1]

[1]Division of Genetics and Bioinformatics, IBHV, University of Copenhagen, Grnnegrdsvej 3, DK-1870 Frederiksberg, Denmark

[2]The Wellcome Trust/Cancer Research UK Gurdon Institute, Cambridge, CB2 1QN, UK

Email: Karsten Scheibye-Knudsen - scheibye@genome.ku.dk; Susanna Cirera - scs@life.ku.dk; Michael J. Gilchrist - m.gilchrist@gurdon.cam.ac.uk; Merete Fredholm - mf@life.ku.dk; Jan Gorodkin*- gorodkin@genome.ku.dk;

*Corresponding author

Supplementary Figure 1:

Figures showing the coverage of the different libraries in the COX I region (from position 5270 to 6814).The groups were divided by the the hierarchical clustering, based on coverage of the different parts of the mitochondrion. Individual libraries can be seen in additional data file 3.

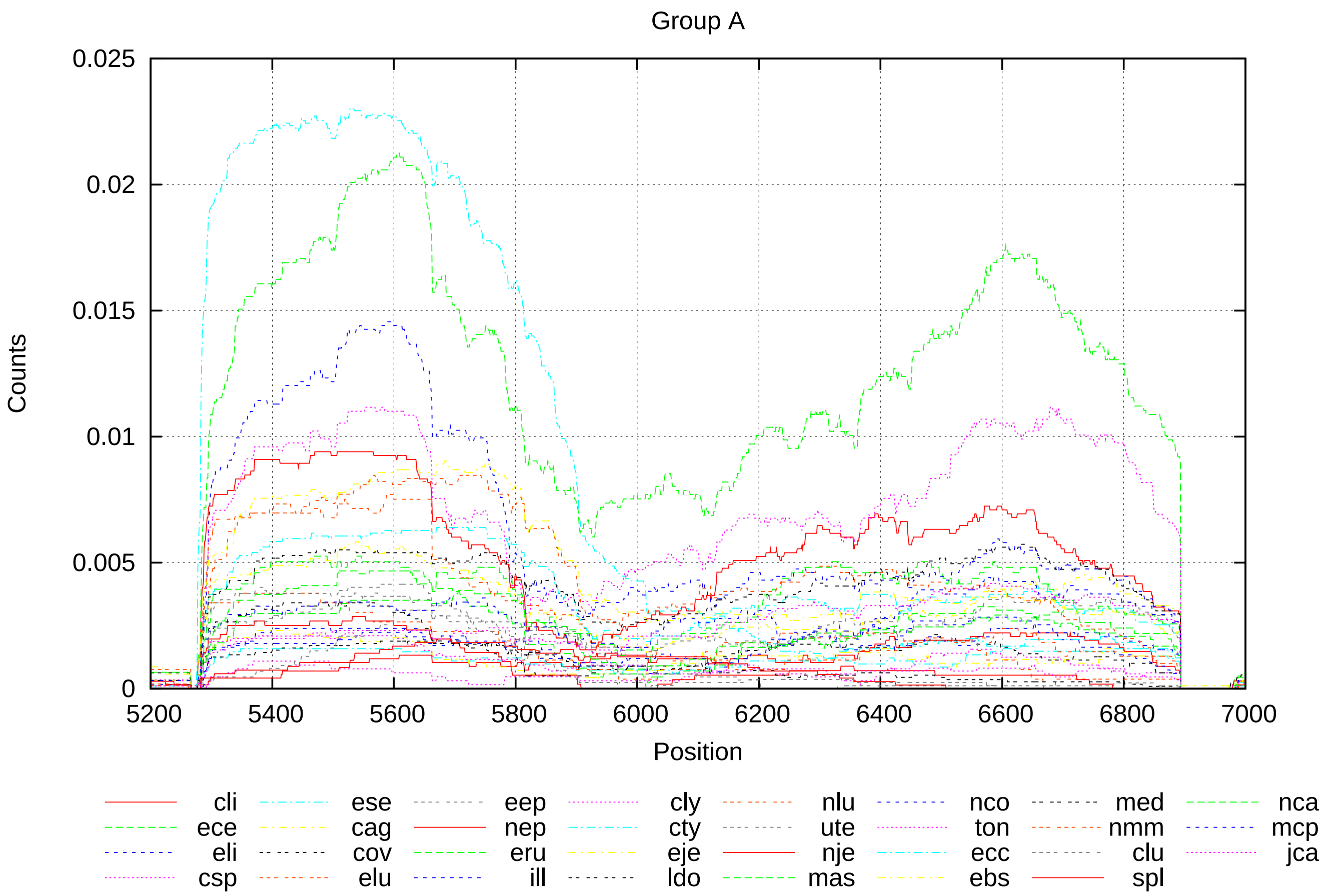
Group A
Counts
Position
0
0.005
0.01
0.015
0.02
0.025
5200
5400
5600
5800
6000
6200
6400
6600
6800
7000
cli
ece
eli
csp
ese
cag
cov
elu
eep
nep
eru
ill
cly
cty
eje
ldo
nlu
ute
nje
mas
nco
ton
ecc
ebs
med
nmm
clu
spl
nca
mcp
jca

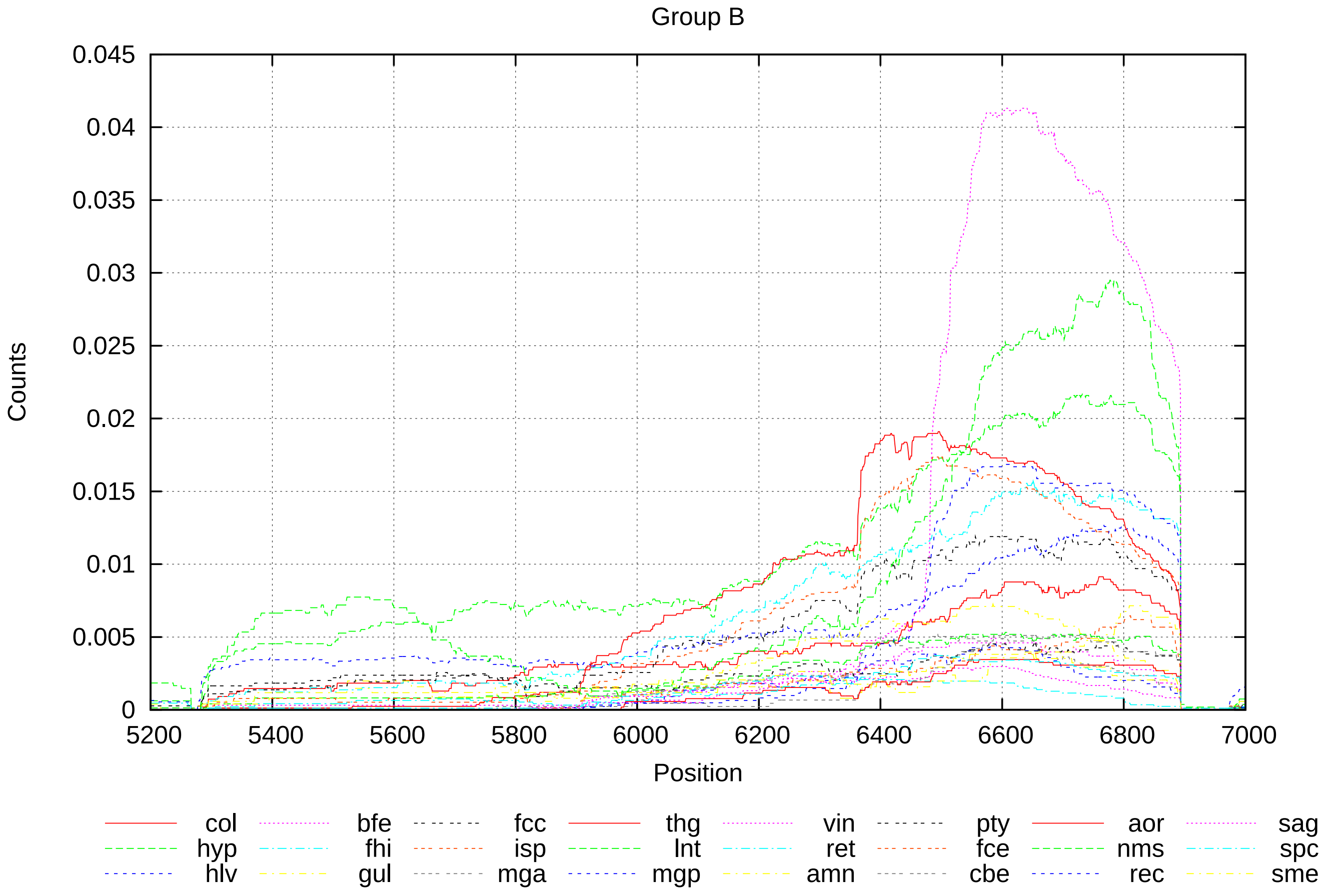
Group B
Counts
Position
0
0.005
0.01
0.015
0.02
0.025
0.03
0.035
0.04
0.045
5200
5400
5600
5800
6000
6200
6400
6600
6800
7000
col
hyp
hlv
bfe
fhi
gul
fcc
isp
mga
thg
lnt
mgp
vin
ret
amn
pty
fce
cbe
aor
nms
rec
sag
spc
sme

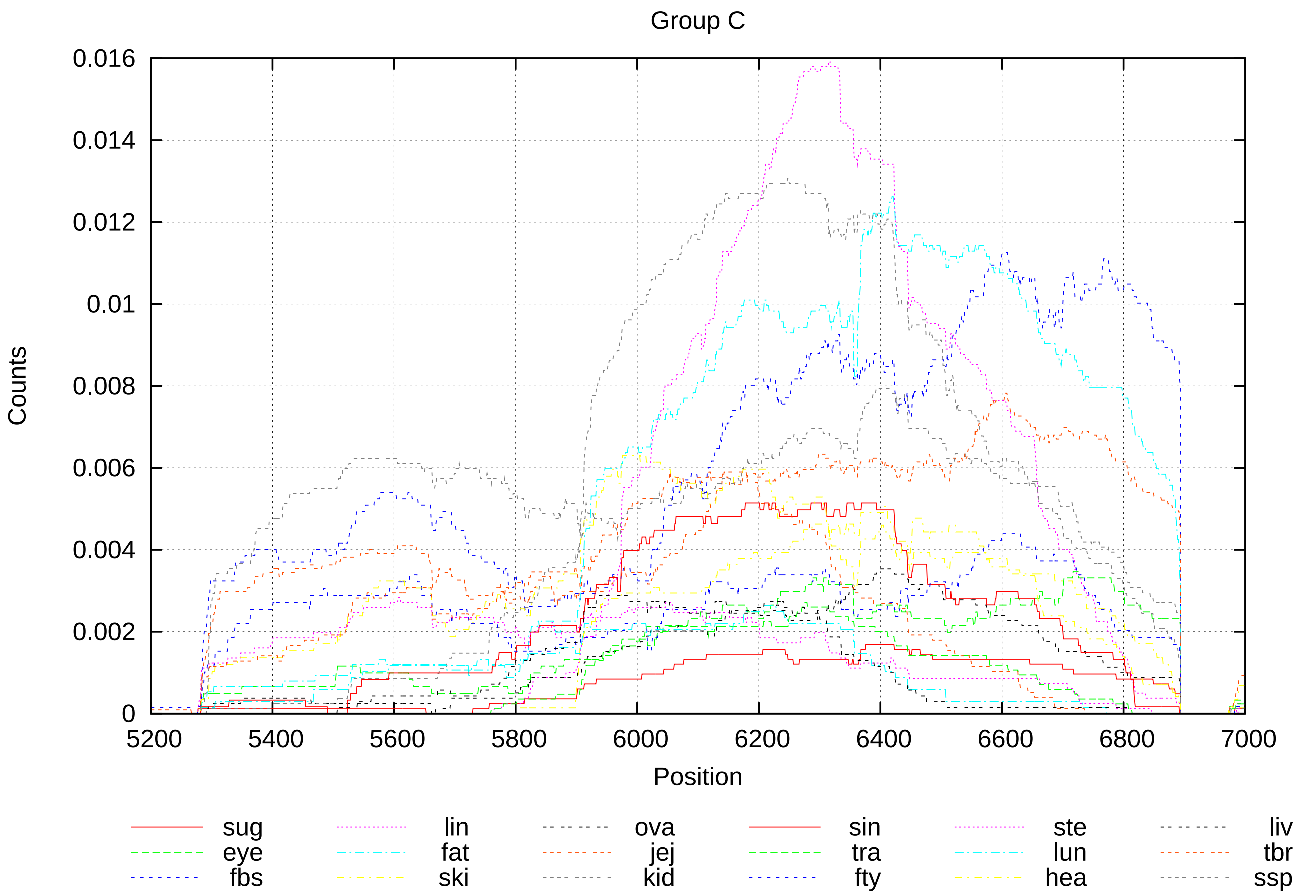
Group C
Counts
Position
0.016
0.014
0.012
0.01
0.008
0.006
0.004
0.002
0
5200
5400
5600
5800
6000
6200
6400
6600
6800
7000
sug
eye
fbs
lin
fat
ski
ova
jej
kid
sin
tra
fty
ste
lun
hea
liv
tbr
ssp

Supplementary Figure 2:

Figure showing the clustering of a nerve tissue libraries on the COX I gene, ie. a subclustering of Figure 4.

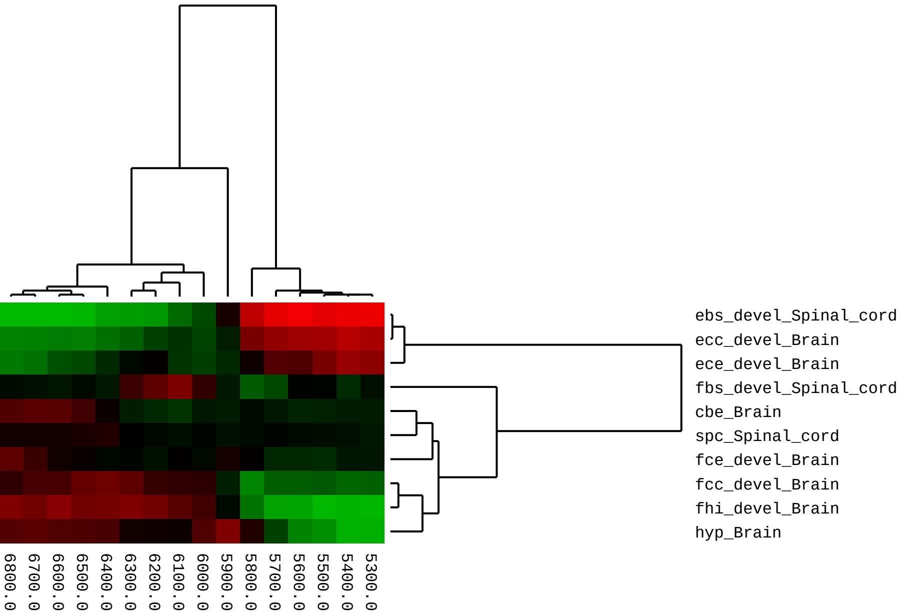
ebs_devel_Spinal_cord
ecc_devel_Brain
ece_devel_Brain
fbs_devel_Spinal_cord
cbe_Brain
spc_Spinal_cord
fce_devel_Brain
fcc_devel_Brain
fhi_devel_Brain
hyp_Brain
6800.0
6700.0
6600.0
6500.0
6400.0
6300.0
6200.0
6100.0
6000.0
5900.0
5800.0
5700.0
5600.0
5500.0
5400.0
5300.0
